# Supplementary material for: β1-Integrin Accumulates in Cystic Fibrosis Luminal Airway Epithelial Membranes and Decreases Sphingosine, Promoting Bacterial Infections
Source: Cell Host Microbe. 2017 Jun 14;21(6):707–718.e8. doi: 10.1016/j.chom.2017.05.001 (PMC5475347; doi:10.1016/j.chom.2017.05.001)
Supplement: Document S1. Figures S1–S7 and Tables S1–S4 [file mmc1.pdf]

Cell Host & Microbe, Volume *21*

Supplemental Information

β1-Integrin Accumulates in Cystic Fibrosis Luminal Airway Epithelial Membranes and Decreases Sphingosine, Promoting Bacterial Infections

Heike Grassmé, Brian Henry, Regan Ziobro, Katrin Anne Becker, Joachim Riethmüller, Aaron Gardner, Aaron P. Seitz, Joerg Steinmann, Stephan Lang, Christopher Ward, Edward H. Schuchman, Charles C. Caldwell, Markus Kamler, Michael J. Edwards, Malcolm Brodlie, and Erich Gulbins

Supplementary legends

Supplementary Table 1, related to Figure 3B: Quantification of western blot studies in Figure 3B

The western blot studies shown in Fig. 3B were quantified and normalized to actin. The ratio of the signal intensity of β1-integrin to actin is given. Shown is the mean ± SD, n=6, ANOVA, overall p-value <0.001.

Supplementary Table 2, related to 4C: Quantification of western blot studies of Figure 4C

The western blot studies shown in Fig. 4C were quantified and the ratio of the signal intensity of acid ceramidase to actin was calculated. Shown is the mean ± SD, n=4, ANOVA, overall p-value <0.001.

Supplementary Table 3, related to Figures 3 and 4 and Figure S3A: Quantification of the degradation kinetics of β1-integrin

The β1-integrin signals obtained in the western blot studies shown in Fig. S3 were quantified and normalized to actin. Given are the mean values ± SD, n=4, ANOVA, overall p-value <0.001.

Supplementary Table 4, related to Figures 3 and 4 and Fig. S3B: Quantification of the internalization kinetics of β1-integrin

Shown is the quantification of the β1-integrin signal in Fig. S3B normalized to actin. Given are the mean values ± SD, n=4, ANOVA, overall p-value <0.001.

Figure S1, related to Figure 1 and 2: Quantification and control staining

(**A**, **B**) Quantification of the specific and isotype antibody control staining of Fig. 1A is shown in Fig. S1A, of Fig. 1B in Fig. S1B. The fluorescence intensity of 20 random areas of the luminal membrane corresponding to 20 cells per sample was quantified using Image J. We analyzed lung sections from 6 mice, thus, the fluorescence intensities of a total of 120 areas (corresponding to 120 cells) were averaged in panel **A** and **B**. Samples were stained with Cy3-labelled anti-β1-integrin or isotype control rat IgG. An example for the background staining with rat IgG (isotype control for anti-β1-integrin antibodies) followed by Cy3-coupled anti-rat antibodies is displayed.

Shown is the mean ± SD, n=6, t-test. (**C**) The quantification of western blots displayed in Fig. 1C is given. Western blots were analyzed using Image J. Shown is the mean ± SD from 4 independent experiments. Actin blots served to normalize the β1-integrin signal. Significant differences were calculated using ANOVA, overall p-value<0.001. (**D, E**) To exclude a general alteration of membranes in CF samples, we stained lung sections with Cy3-Annexin V, which binds to phosphatidylserine. Shown is a typical result from 6 independent experiments. No differences between wildtype (WT) and CF samples were detected. The intensity of the Annexin V staining was quantified as above. The ratio between the fluorescence intensities of Annexin V and β1-integrin staining confirms the accumulation of β1-integrin in the luminal membrane of CF cells. Shown are typical examples and the mean ± SD, n=6, ANOVA, overall p<0.001. (**F**) Lung sections from WT and CF mice were stained with anti-β2-integrin antibodies followed by Cy3-labelled secondary antibodies. The staining is mainly in the alveoli and does not differ between WT and CF samples. Shown is a typical result from 6 independent experiments. (**G**) The figure illustrates the anatomical structure of the epithelial cell layer with the lateral membranes in white color and the luminal membranes in yellow. The panel is from Figure 1A.

(**H-J**) The quantification of the specific and isotype antibody control staining displayed in Fig. 2A is shown in Fig. S1H, of Fig. 2B in Fig. S1I, of Fig. 2D in Fig. S1J. An example for the background staining with mouse IgM followed by Cy3-coupled anti-mouse IgM antibodies (isotype antibody control for anti-ceramide and anti-sphingosine antibodies) is displayed in Fig. S1H. Shown is the mean ± SD, n=6 (H-I), n=4 (J), ANOVA, overall p-value <0.001.

Figure S2, related to Figures 2 and 3: Quantifications and the effect of internalization of the gp-linked protein Art1 on surface β1-integrin, ceramide and sphingosine

(**A-D**) The quantification of the specific and isotype antibody control staining displayed in Fig. 2E is given in Fig. S2A, of Fig. 3A and D in Fig. S2B, of Fig. 4A in Fig. S2C, of Fig. 4E in Fig. S2D. The fluorescence intensities were determined as in Fig. S1A. An example for the background staining with mouse IgG (isotype antibody control for anti-acid ceramidase antibodies) is demonstrated in Fig. S2C. Shown is the mean ± SD, n=4 (A) or n=6 (B-D), ANOVA, overall p-value <0.001.

(**E**) The trachea surface was incubated with anti-Art1 antibodies for 30 min at 37°C or

left untreated, washed and incubated with anti-Art1 antibodies or isotype controls for an additional 30 min at 4°C. The epithelial cells were then scraped from the surface of the trachea, incubated with FITC-coupled secondary antibodies and surface staining of Art1 was determined by flow cytometry. The results show that incubation with anti-Art-1 antibodies did not alter surface β1-integrin. Shown are the mean ± SD of the mean fluorescence values of 3 independent experiments. Statistical differences were analyzed by ANOVA. The decreased mean fluorescence corresponds very likely to an internalization of Art1. (**E-G**) Internalization of Art1 does not change surface expression of β1-integrin in CF cells as determined by flow cytometry, surface ceramide and surface sphingosine as determined by in situ kinase assays. Given are the mean ± SD from 3 independent experiments, ANOVA, overall p-value <0.001.

Figure S3, related to Figures 3 and 4: Kinetics of β1-integrin degradation, internalization and effects on acid ceramidase activity, surface ceramide and sphingosine

(**A and B**) Degradation kinetics of surface β1-integrin: The surface of the trachea of wildtype (WT) and CF mice was biotinylated, washed and cells were lysed after the indicated time of incubation with RGD-peptides. The samples were lysed, precipitated using Streptavidin-coupled agarose and blotted with anti-β1-Integrin antibodies (clone MB1.2) and HRP-coupled secondary antibodies (Santa Cruz Inc.). β1-integrin is rapidly degraded in WT epithelial cells, but remains stable in CF epithelial cells. Displayed is a typical result from 4 independent studies. The quantification is given in supplementary Table 3.

(**B**) Uptake kinetics of surface β1-integrin: The surface of tracheae was biotinylated, washed and the samples were incubated for 0, 30, 60 and 90 min at 37°C in the presence or absence of acid ceramidase (Ac) or binding of RGD-peptides. Surface integrin was then bound to anti-β1-Integrin antibodies clone MB1.2 for 30 min at 4°C, the samples were lysed, precipitated using protein A/G agarose and developed with HRP-coupled streptavidin. Consumption of ceramide by Ac or binding of RGD-peptides allowed rapid internalization of biotinylated surface β1-Integrin in CF cells. Shown is a typical result from 4 independent studies.

(**C, D**) Infection of tracheal epithelial cells from WT and CF mice with *P. aeruginosa* strains 762, ATCC 27853 or 1242 for 15 min results in an activation of the acid

sphingomyelinase (Asm) as determined by consumption of [^{14}C]sphingomyelin (**C**), but has almost no effect on the surface expression of β1-Integrin in CF cells determined by flow cytometry analysis after binding of FITC-coupled anti-β1-Integrin antibodies to the cells (**D**). Shown are the mean values ± SD, n=5, ANOVA, overall p-value <0.001.

(E-H) Addition of anti-β1-integrin antibodies 9EG7 as β1-integrin ligand to the intact surface of the trachea isolated from WT and CF mice results in internalization of β1-integrin (**E**), increased activity of acid ceramidase (**F**), reduction of surface ceramide (**G**) and an increase of surface sphingosine (**H**). Immobilization of the anti-β1-integrin 9EG7 antibodies to agarose beads blocking its internalization prevented reduction of ceramide and the increase of sphingosine in CF cells. Inhibition of the acid ceramidase by N-oleoylethanolamine or carmofur did not affect internalization of β1-integrin upon antibody binding (**E**), but prevented the consumption of ceramide and the generation of sphingosine after incubation with anti-β1-integrin 9EG7 antibodies. Internalization was determined by flow cytometry after staining the still intact surface with FITC-coupled anti-β1-integrin antibodies clone MB1.2 followed by isolation of single epithelial cells. Surface acid ceramidase activity was determined by measuring the consumption of [^{14}C]ceramide added to the tracheal surface, surface ceramide and sphingosine were measured by in situ kinase assays. Shown are the mean ± SD of the mean fluorescence of the flow cytometry studies (background with isotype controls antibodies was subtracted), the enzyme activities or the amounts of ceramide and sphingosine from 4 independent experiments. Statistical differences were determined by ANOVA, overall p-value <0.001.

Figure S4, related to Figure 4 and 5: Surface β1-integrin in CF epithelial cells physically interacts with ceramide and determines pulmonary *P. aeruginosa* infections

(**A**) Immunoprecipitation of β1-integrin from CF cells co-precipitated ceramide, while no ceramide was detected in the immunoprecipitates from wildtype (WT) cells or in control immunoprecipitates from CF cells. Treatment of the surface of CF trachea with acid ceramidase for 15 min at 37°C after binding of the anti-β1-integrin antibodies complexed to protein A/G agarose but prior to lysis reduced the amount of ceramide bound to β1-integrin-immunoprecipitates. In addition, incubation with anti-ceramide antibodies that have been previously shown to displace receptors such as

CD95 from ceramide-enriched membrane domains (Grassmé et al., 2000, Rotolo et al., 2012) either by simple steric competition or by a higher affinity of the antibody to ceramide, abrogated the association of ceramide with β1-integrin. Isotype control IgM antibodies (Ab) were without effect on the co-precipitation of ceramide with β1-integrin. Incubation of the surface of the trachea from WT animals with 10 μM C_{16}-ceramide resulted in co-precipitation of ceramide with β1-integrin. Ceramide was determined by ceramide kinase assays. (**B**) Incubation with anti-ceramide antibodies resulted in rapid internalization of β1-integrin as measured by flow cytometry after staining intact tracheal surfaces with FITC-coupled anti-β1-integrin antibodies. Conversely, incubation of WT trachea with 10 μM C16 ceramide resulted in surface trapping and expression of β1-integrin.

Shown are the mean ± SD of ceramide or the mean fluorescence of the stained cells (background with isotype controls antibodies was subtracted) from 4 independent experiments, ANOVA, overall p-value <0.001.

(**C**) We intranasally infected CF mice with $5×10^6$ CFU of *P. aeruginosa* strain 762. Please note that the scale bars in Figure S4C and Figure 5A are 10-fold different. These studies showed that the number of bacteria in the lung correlates linearly with the intranasally applied CFU. Inhalation of anti-β1-integrin antibodies or RGD peptides rescued each mouse strain from infection with *P. aeruginosa*. Control peptides or isotype-matched irrelevant antibodies did not alter the massive infection of these mouse strains. Mean ± SD, n=6, ANOVA, overall p<0.001.

(**D, E**) Inhalation of nebulized anti-β1-integrin antibodies (Ab) or RGD peptides 1 hr prior infection intranasal application with 10^5 (**D**) or 10^8 (**E**) CFU *P. aeruginosa* strain ATCC 27853 greatly reduced the number of bacteria that reach the lower part of the trachea (**D**) and thereby prevents pulmonary infection after (**E**). Shown are the mean ± SD of pulmonary *P. aeruginosa* CFUs, n=6. Statistical differences were determined by t-test or ANOVA, overall p-value <0.001 in all subpanels.

(**F**) Survival studies after *P. aeruginosa* infection revealed that acute infection with *P. aeruginosa* strains 762 or ATCC 27853 resulted in death of CF mice within 96 hrs, which was abrogated by inhalation of acid ceramidase, sphingosine or RGD peptides. The dose of the mucoid strains used above to obtain a (sub-)chronic infection was not high enough to induce a lethal infection. We therefore used a higher infection dose ($1x10^8$ CFU of *P. aeruginosa* 1242 and 1245). This infection resulted in death of CF mice, which was prevented by inhalation of Ac or sphingosine. The

panel shows Kaplan Meyer-survival curves of CF^{MHH} or WT mice infected with 5×10^7 CFU of *P. aeruginosa* strain 762 or 10^8 CFU of ATCC strains 27853, 1242 and 1245. $p<0.001$ for all treated or WT curves compared to corresponding untreated curves, log-rank test.

(**G**) WT mice were inhaled with 2 μg/ml monoclonal anti-sphingosine antibodies diluted in 0.9% saline or as control with saline only and infected 45 min later with $5x10^7$ CFU of *P. aeruginosa* strain 762 or $1x10^8$ CFU of strains ATCC27853 or 1242, the lungs were removed 6 hrs later, homogenized, aliquots were plated on TSA plates, grown o/n and colonies were counted. Given are the mean ± SD of pulmonary *P. aeruginosa* CFUs, n=5. Statistical differences were determined by t-test or ANOVA, overall p-value <0.001 in all subpanels.

Figure S5*, *related to Figure 5: Inhalation of sphingosine or acid ceramidase rapidly kills pulmonary mucoid and non-mucoid *P. aeruginosa*

Mice were infected with $5x10^7$ CFU *P. aeruginosa* 762 and inhaled with sphingosine (SPH) or acid ceramidase (Ac) 1 hr after infection. Lungs were removed 6 hrs (**A**) or 48 hrs (**C**) after infection. Infection with *P. aeruginosa* 1242 was performed once daily at $1x10^7$ CFUs for 5 days, mice were inhaled with sphingosine or acid ceramidase 1 hr (**B**) or 48 hrs (**C**) after the last infection and sacrificed 5 hrs (**B**) or 48 hrs after inhalation. Lungs were removed, fixed in 4% paraformaldehyde, embedded in paraffin, sections were performed and stained with FITC-labelled anti-Gr1 (Ly6C/G) and Cy3-labelled anti-*P. aeruginosa* antibodies and analyzed by confocal microscopy. The sections obtained from mice 48 hrs after treatment were only stained with FITC-labelled anti-Gr1 (Ly6C/G), since the number of bacteria in the lung at this time point is too low to be detected by staining 5 μm sections with *P. aeruginosa* antibodies. The results show that sphingosine and acid ceramidase rapidly kill the bacteria within a few hours followed by resolution of the inflammation over the next few days, while untreated CF mice show massive amounts of bacteria and GR1-positive myeloid cells 6 hrs and even 96 hrs after infection. Panel **D** shows the control staining of uninfected mice. The first time given in the headlines of the figures refers to the time of treatment after the last infection, the 2nd time to the time of the removal of the lung after infection. Data are representative for 6 experiments with very similar results.

Figure S6, related to Figure 5: Surface sphingosine kills pulmonary mucoid and non-mucoid *P. aeruginosa* at concentrations that are present in the tracheal surface fluid

(**A**) The non-mucoid *P. aeruginosa* strains 762 and ATCC 27853 and the mucoid strains 1242 and 1245 were incubated with increasing concentrations of sphingosine in a buffer, which mimics the ion composition of the airway surface liquid (Borowitz et al, 1999). Bacteria (1000 CFU) were incubated for 60 min with the indicated concentrations of sphingosine, aliquots were plated on TSB plates and CFU were counted on the next day. Shown is the mean ± SD from 4 independent experiments, ANOVA, overall p-value <0.001. (**B**) Mice were infected with $1x10^8$ CFU *P. aeruginosa* strains ATCC 27853 or 1242 or left uninfected, in addition, mice were inhaled with acid ceramidase (Ac) or sphingosine (SPH). Tracheal surface fluid was isolated, extracted and the sphingosine concentration was determined using a sphingosine kinase assay. Shown are the mean ± SD from 4 independent measurements, ANOVA, overall p-value <0.001.

(**C-E**) To test whether sphingosine regulated by β1-integrin is sufficient to serve as first line of defense in the upper airways and the bronchi killing bacteria without an inflammatory response, we infected the intact epithelial cell layer of isolated trachea from CF or WT mice with *P. aeruginosa* and determined killing of *P. aeruginosa*. Trachea from WT or CF mice were isolated and incubated with anti-sphingosine (SPH) antibodies, sphingosine (SPH), acid ceramidase (Ac), sphingosine kinase, or octylglucopyranoside (OGP) as control. Further, the trachea was fixed for 10 min with 0.25% paraformaldehyde (PFA) or permeabilized for 5 min with 0.1% Triton X-100. The tracheae were then infected with 200 CFU of *P. aeruginosa* strains 762, ATCC 27853 or 1242 for 60 min, homogenized and the samples was spotted onto TSA plates. Bacteria were counted after 24 hrs growth. The results show that WT tracheal epithelial cells kill *P. aeruginosa*, while CF epithelial cells fail. The reconstitution of sphingosine restores killing by CF epithelial cells. Neutralization of sphingosine by anti-sphingosine antibodies or phosphorylation of sphingosine by sphingosine kinase prevents killing of the bacteria by WT cells or treated CF cells. The permeabilization studies served to exclude a signalling function of sphingosine.

(**F**) In situ sphingosine kinase assays were performed to confirm that the treatment with acid ceramidase (Ac) or sphingosine (SPH) reconstituted sphingosine on the

surface of CF trachea to physiological levels. Displayed are the mean ± SD from 5 independent measurements, ANOVA, overall p-value <0.001.

Figure S7, related to Figures 6 and 7: Ectopic expression of β1-integrins causes ceramide accumulation in human airway CF cells

Ceramide is severely upregulated on the luminal pole of human CF bronchial epithelial cells (**A**), nasal epithelial cells (**B**) and nasal polyps (**C**). Normalization of ectopic β1-integrin expression or incubation of freshly isolated epithelial cells with acid ceramidase reduced ceramide levels in CF cells (**B**). Sections or cells were stained as indicated with Cy3-labelled anti-ceramide antibodies. Pre-incubations were performed with RGD-peptides, HUTS-4 anti-β1-integrin antibodies (α-Int.-Ab) or acid ceramidase (Ac). The luminal membrane is indicated by arrows. Shown are representative results from each 5 CF or 4 healthy individuals (all lung sections and freshly isolated epithelial cells) or each 4 CF or healthy persons (nasal polyps). (**D**) CF or healthy control nasal epithelial cells were counted, aliquoted and infected with *P. aeruginosa* strains 762 or ATCC 27853 at a multiplicity of infection of 1 cell : 0.5 bacteria. The samples were incubated at 37°C for 60 min, plated on LB agar plates, cultured overnight and *P. aeruginosa* colonies were counted. CF cells have a reduced capability to kill *P. aeruginosa*, which is corrected by pre-incubation with anti-β1-integrin antibodies (HUTS-4), RGD-peptides, acid ceramidase or sphingosine. (**E**) Acid ceramidase activity is reduced in primary bronchial epithelial cells from people with CF (n=4) compared to non-CF individuals (n=5).

The fluorescence intensities in the luminal membrane of all fluorescence microscopy studies were quantified using Image J and are given in arbitrary units (a.u.). Given are the mean ± SD, n = 4 or 5, p-values are given in the figures, t-test for comparison of 2 values and ANOVA and post-hoc Student´s t-tests for all pairwise comparisons applying Bonferroni correction for multiple comparisons. Overall p-value for ANOVA is <0.001 in all panels. E = epithelial cell layer.

Supplementary Table 1

Ratio β-Integrin-signal intensity/Actin signal intensity [a.u], related to Fig. 3B

WT	0.014 ± 0.004
CF^{MHH}	1.23 ± 0.092 ($p<0.001$ compared to WT)
CF^{MHH} + RGD	0.051 ± 0.032 ($p<0.001$ compared to CF^{MHH})
CF^{MHH} + ctrl. Peptide	1.23 ± 0.092 ($p<0.001$ compared to CF^{MHH} + RGD)
CF^{MHH} + anti-β1-Integrin-antibody	0.123 ± 0.022 ($p<0.001$ compared to CF^{MHH})
CF^{MHH} + ctrl. Antibody	1.23 ± 0.095 ($p<0.001$ compared to CF^{MHH} + anti-β1-Int)
WT	0.013 ± 0.004
$Cftr^{-/-}$	1.19 ± 0.059 ($p<0.001$ compared to WT)
$Cftr^{-/-}$ + RGD	0.030 ± 0.013 ($p<0.001$ compared to $Cftr^{-/-}$)
$Cftr^{-/-}$ + ctrl. Peptide	1.17 ± 0.161 ($p<0.001$ compared to $Cftr^{-/-}$ + RGD)
$Cftr^{-/-}$ + anti-β1-Integrin-antibody	0.090 ± 0.046 ($p<0.001$ compared to $Cftr^{-/-}$)
$Cftr^{-/-}$ + ctrl. Antibody	1.26 ± 0.135 ($p<0.001$ compared to $Cftr^{-/-}$ + anti-β1 Int)
unspecific	0.005 ± 0.002

Supplementary Table 2

Ratio acid ceramidase (α-subunit)-signal intensity/Actin signal intensity [a.u.], related to Fig. 4C

WT	1.47 ± 0.078
$Cftr^{-/-}$	0.027 ± 0.011 ($p<0.001$ compared to WT)
$Cftr^{-/-}$ + RGD	1.48 ± 0.112 ($p<0.001$ compared to $Cftr^{-/-}$)
$Cftr^{-/-}$ + ctrl. Peptide	0.033 ± 0.012 ($p<0.001$ compared to $Cftr^{-/-}$ + RGD)
$Cftr^{-/-}$ + anti-β1-Integrin	1.47 ± 0.127 ($p<0.001$ compared to $Cftr^{-/-}$)
$Cftr^{-/-}$ + ctrl. Antibody	0.042 ± 0.015 ($p<0.001$ compared to $Cftr^{-/-}$ + anti-β1-Integrin)

Ratio acid ceramidase (β-subunit)-signal intensity/Actin signal intensity [a.u.], related to Fig. 4C

WT	0.935 ± 0.083
$Cftr^{-/-}$	0.021 ± 0.007 ($p<0.001$ compared to WT)
$Cftr^{-/-}$ + RGD	0.840 ± 0.062 ($p<0.001$ compared to $Cftr^{-/-}$)
$Cftr^{-/-}$ + ctrl. Peptide	0.043 ± 0.021 ($p<0.001$ compared to $Cftr^{-/-}$ + RGD)
$Cftr^{-/-}$ + anti-β1-Integrin	0.755 ± 0.037 ($p<0.001$ compared to $Cftr^{-/-}$)
$Cftr^{-/-}$ + ctrl. Antibody	0.030 ± 0.001 ($p<0.001$ compared to $Cftr^{-/-}$ + anti-β1-Integrin)

Supplementary Table 3

Ratio β-Integrin-signal intensity/Actin signal intensity [a.u], related to Figures 3 and 4 and Figure S3A

WT-0 min	0.65 ± 0.072
WT-60 min	0.09 ± 0.026 ($p<0.001$ compared to WT-0 min)
WT-90 min	0.06 ± 0.001 ($p<0.001$ compared to WT-0 min)
$Cftr^{-/-}$-0 min	1.08 ± 0.1 ($p<0.001$ compared to WT-0 min)
$Cftr^{-/-}$-60 min	0.85 ± 0.06 ($p<0.001$ compared to WT-60 min)
$Cftr^{-/-}$-90 min	0.75 ± 0.07 ($p<0.001$ compared to WT-90 min)
$Cftr^{-/-}$ + RGD-0 min	0.96 ± 0.04 ($p<0.001$ compared to WT-0 min)
$Cftr^{-/-}$ + RGD-60 min	0.39 ± 0.05 ($p<0.001$ compared to $Cftr^{-/-}$-60 min; $p<0.0064$ compared to WT-60 min)
$Cftr^{-/-}$ + RGD-90 min	0.18 ± 0.05 ($p<0.001$ compared to $Cftr^{-/-}$-60 min; $p<0.001$ compared to WT-90 min)

Supplementary Table 4

Ratio β-Integrin-signal intensity/Actin signal intensity [a.u], related to Figures 3 and 4 and Figure S3B

$Cftr^{-/-}$-0 min	0.49 ± 0.030
$Cftr^{-/-}$-30 min	0.48 ± 0.040
$Cftr^{-/-}$-60 min	0.45 ± 0.027
$Cftr^{-/-}$-90 min	0.36 ± 0.031 ($p<0.001$ compared to $Cftr^{-/-}$-0 min)
CF^{MHH}-0 min	0.48 ± 0.022
CF^{MHH}-90 min	0.47 ± 0.017
$Cftr^{-/-}$ + RGD-30 min	0.26 ± 0.013 ($p<0.001$ compared to $Cftr^{-/-}$-30 min)
$Cftr^{-/-}$ + RGD-60 min	0.20 ± 0.017 ($p<0.001$ compared to $Cftr^{-/-}$-60 min)
$Cftr^{-/-}$ + RGD-90 min	0.06 ± 0.018 ($p<0.001$ compared to $Cftr^{-/-}$-90 min)
$Cftr^{-/-}$ + Ac-30 min	0.26 ± 0.001 ($p<001$ compared to $Cftr^{-/-}$-30 min)
$Cftr^{-/-}$ + Ac-60 min	0.16 ± 0.016 ($p<0.001$ compared to $Cftr^{-/-}$-60 min)
$Cftr^{-/-}$ + Ac-90 min	0.03 ± 0.008 ($p<0.001$ compared to $Cftr^{-/-}$-90 min)

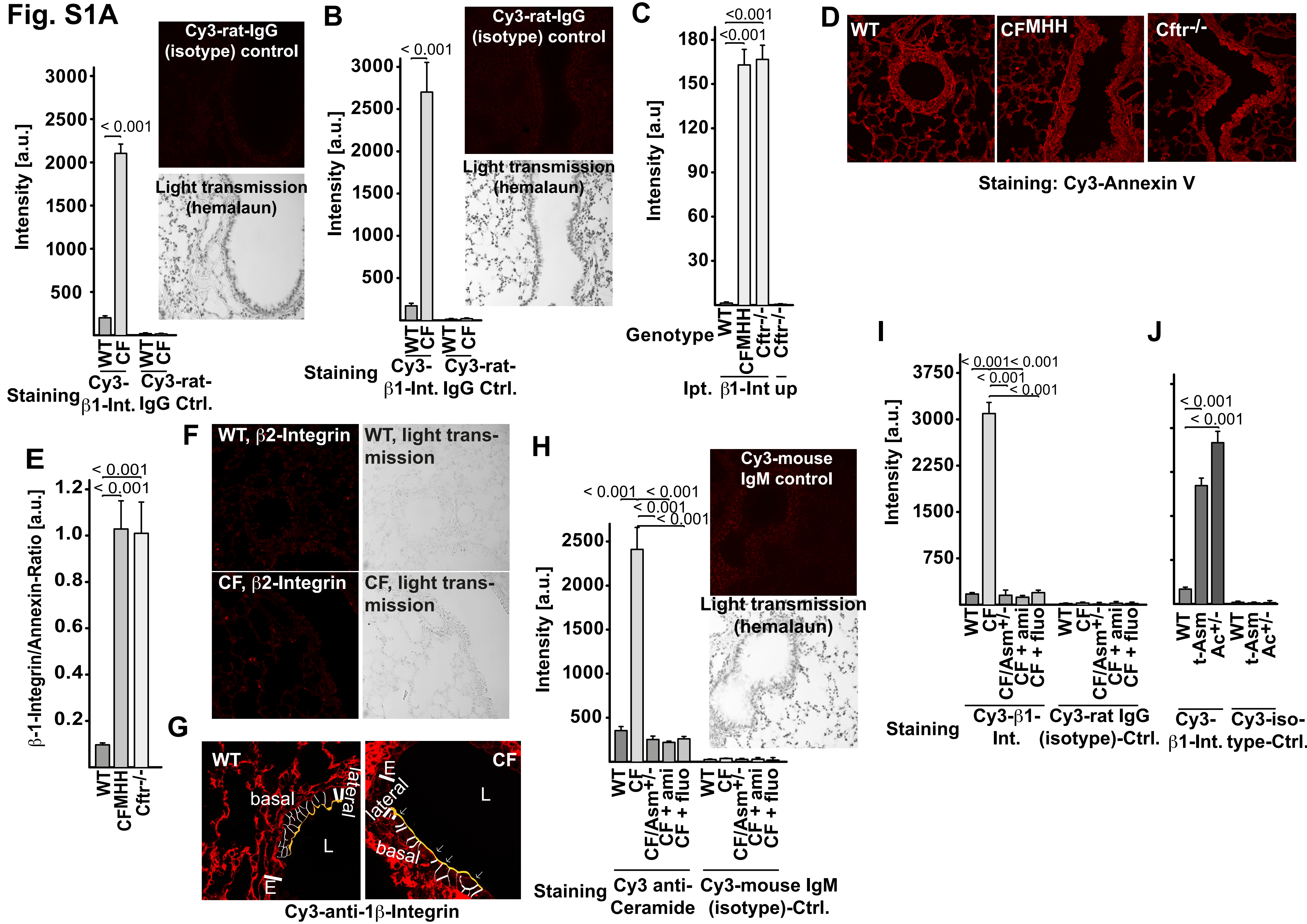

Fig. S1A
Cy3-rat-IgG (isotype) control
Light transmission (hemalaun)
Intensity [a.u.]
< 0.001
WT CF WT CF
Staining Cy3-β1-Int. Cy3-rat-IgG Ctrl.
B
Cy3-rat-IgG (isotype) control
Light transmission (hemalaun)
C
<0.001
<0.001
Genotype WT CFMHH Cftr-/- Cftr-/-
Ipt. β1-Int up
D
WT
CFMHH
Cftr-/-
Staining: Cy3-Annexin V
E
β-1-Integrin/Annexin-Ratio [a.u.]
WT CFMHH Cftr-/-
F
WT, β2-Integrin
WT, light transmission
CF, β2-Integrin
CF, light transmission
G
WT
basal
lateral
E
L
CF
Cy3-anti-1β-Integrin
H
Cy3-mouse IgM control
Light transmission (hemalaun)
WT CF CF/Asm+/- CF + ami CF + fluo
Staining Cy3 anti-Ceramide Cy3-mouse IgM (isotype)-Ctrl.
I
Staining Cy3-β1-Int. Cy3-rat IgG (isotype)-Ctrl.
J
WT t-Asm Ac+/-
Cy3-β1-Int. Cy3-isotype-Ctrl.

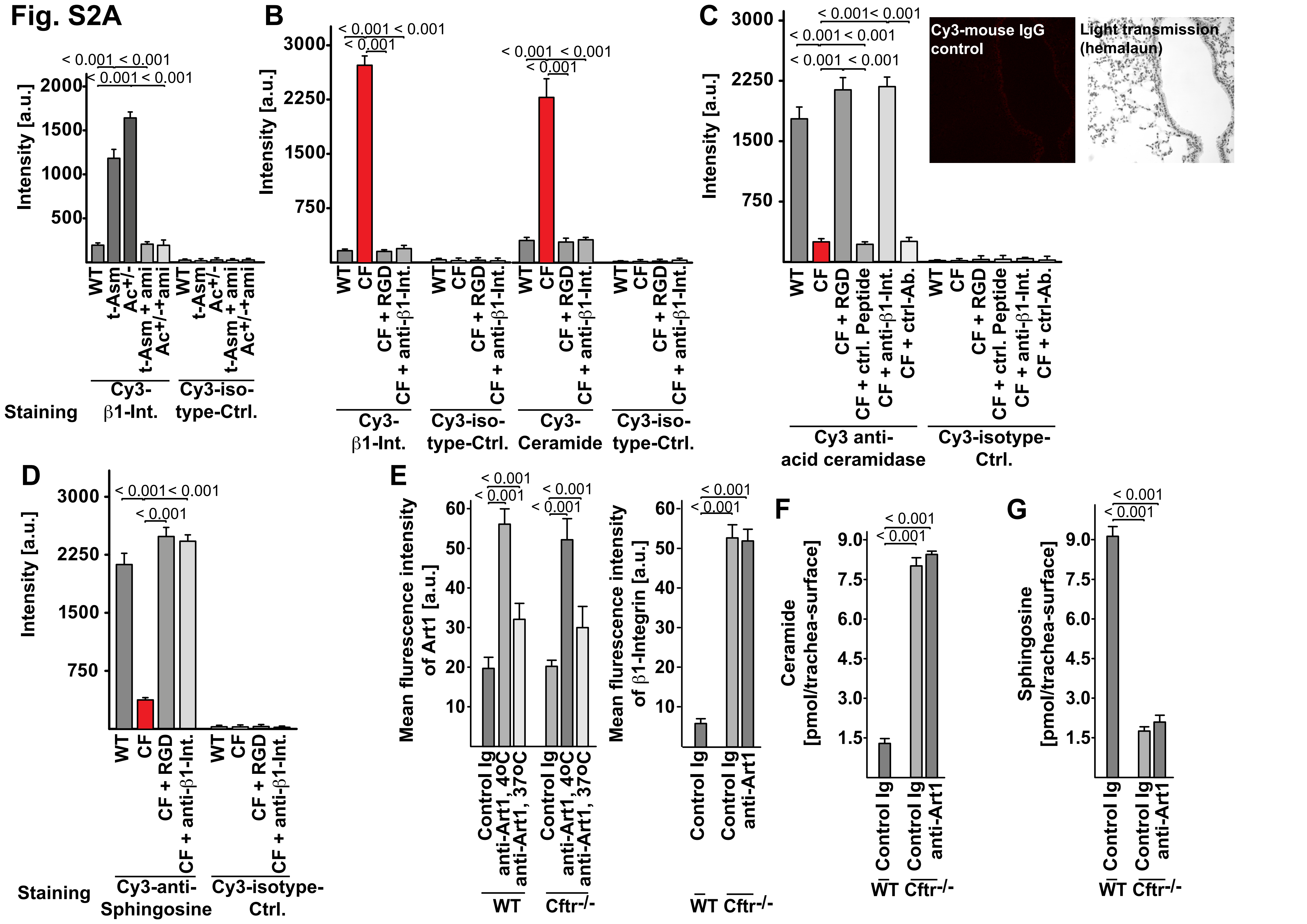

Fig. S2A
B
C
D
E
F
G
Intensity [a.u.]
< 0.001 < 0.001
< 0.001 < 0.001
WT
t-Asm
Ac+/-
t-Asm + ami
Ac+/-+ami
Staining
Cy3-β1-Int.
Cy3-iso-type-Ctrl.
CF
CF + RGD
CF + anti-β1-Int.
Cy3-Ceramide
Cy3-mouse IgG control
Light transmission (hemalaun)
CF + ctrl. Peptide
CF + ctrl-Ab.
Cy3 anti-acid ceramidase
Cy3-isotype-Ctrl.
Cy3-anti-Sphingosine
Mean flurescence intensity of Art1 [a.u.]
Mean flurescence intensity of β1-Integrin [a.u.]
Control Ig
anti-Art1, 4oC
anti-Art1, 37oC
anti-Art1
WT
Cftr-/-
Ceramide [pmol/trachea-surface]
Sphingosine [pmol/trachea-surface]

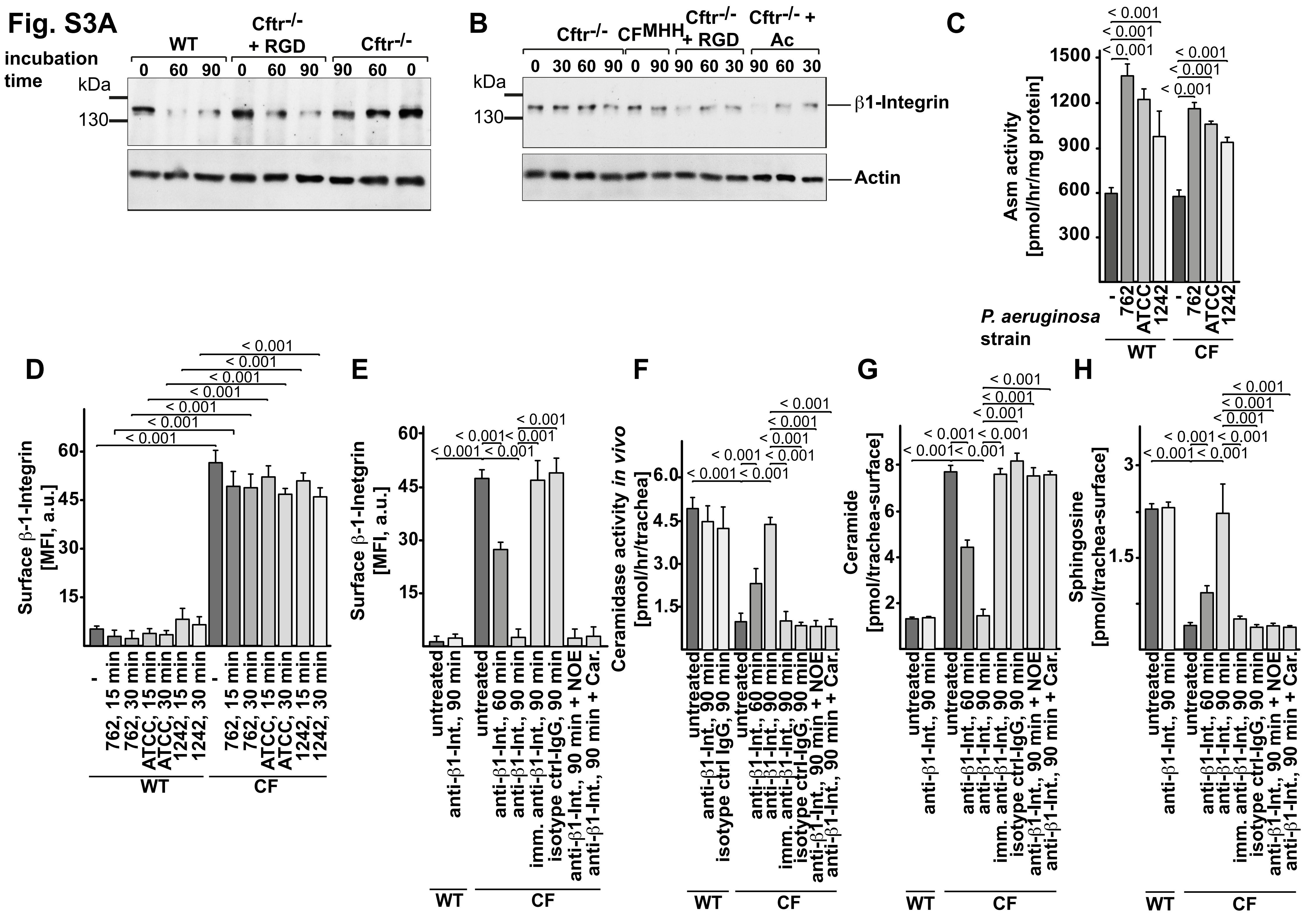
Fig. S3A
WT
Cftr-/- + RGD
Cftr-/-
incubation time
0 60 90 0 60 90 90 60 0
kDa
130
B
Cftr-/-
CFMHH
Cftr-/- + RGD
Cftr-/- + Ac
0 30 60 90 0 90 90 60 30 90 60 30
β1-Integrin
Actin
C
Asm activity [pmol/hr/mg protein]
P. aeruginosa strain
- 762 ATCC 1242
WT CF
< 0.001
D
Surface β-1-Integrin [MFI, a.u.]
762, 15 min
762, 30 min
ATCC, 15 min
ATCC, 30 min
1242, 15 min
1242, 30 min
E
Surface β-1-Inetgrin [MFI, a.u.]
untreated
anti-β1-Int., 90 min
anti-β1-Int., 60 min
imm. anti-β1-Int., 90 min
isotype ctrl-IgG, 90 min
anti-β1-Int., 90 min + NOE
anti-β1-Int., 90 min + Car.
F
Ceramidase activity in vivo [pmol/hr/trachea]
isotype ctrl IgG, 90 min
G
Ceramide [pmol/trachea-surface]
H
Sphingosine [pmol/trachea-surface]

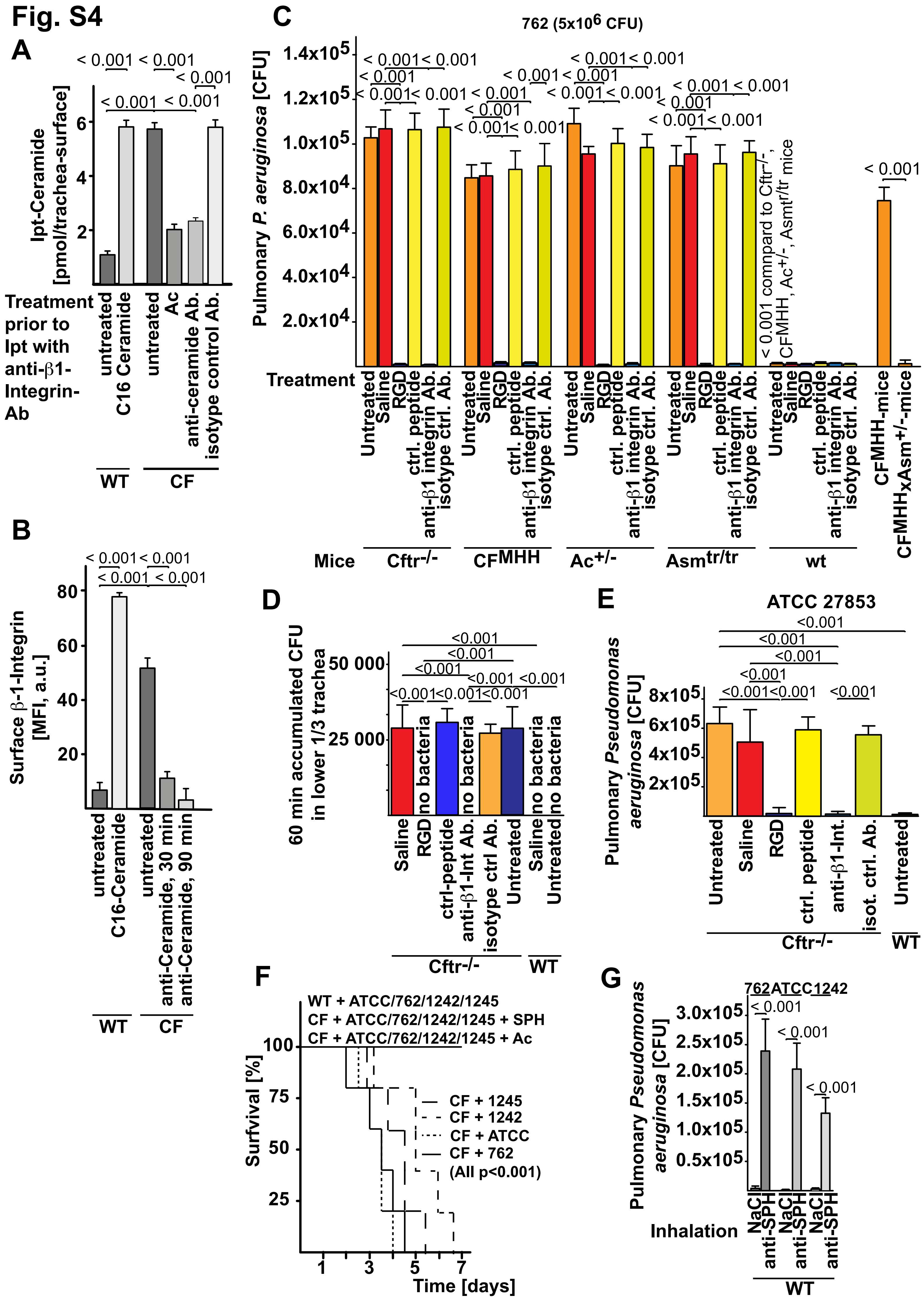

Fig. S4
A
Ipt-Ceramide [pmol/trachea-surface]
Treatment prior to Ipt with anti-β1-Integrin-Ab
untreated
C16 Ceramide
untreated
Ac
anti-ceramide Ab.
isotype control Ab.
WT
CF
< 0.001
B
Surface β-1-Integrin [MFI, a.u.]
untreated
C16-Ceramide
untreated
anti-Ceramide, 30 min
anti-Ceramide, 90 min
WT
CF
C
762 (5x10^6 CFU)
Pulmonary P. aeruginosa [CFU]
Treatment
Untreated
Saline
RGD
ctrl. peptide
anti-β1 integrin Ab.
isotype ctrl. Ab.
Mice
Cftr-/-
CFMHH
Ac+/-
Asmtr/tr
wt
CFMHH-mice
CFMHHxAsm+/--mice
< 0.001 compard to Cftr-/-, CFMHH, Ac+/-, Asmtr/tr mice
D
60 min accumulated CFU in lower 1/3 trachea
Saline
RGD
ctrl-peptide
anti-β1-Int Ab.
isotype ctrl Ab.
Untreated
no bacteria
Cftr-/-
WT
E
ATCC 27853
Pulmonary Pseudomonas aeruginosa [CFU]
Untreated
Saline
RGD
ctrl. peptide
anti-β1-Int.
isot. ctrl. Ab.
Cftr-/-
WT
F
WT + ATCC/762/1242/1245
CF + ATCC/762/1242/1245 + SPH
CF + ATCC/762/1242/1245 + Ac
Survival [%]
Time [days]
CF + 1245
CF + 1242
CF + ATCC
CF + 762
(All p<0.001)
G
762 ATCC 1242
Pulmonary Pseudomonas aeruginosa [CFU]
Inhalation
NaCl
anti-SPH
WT

Fig. S5

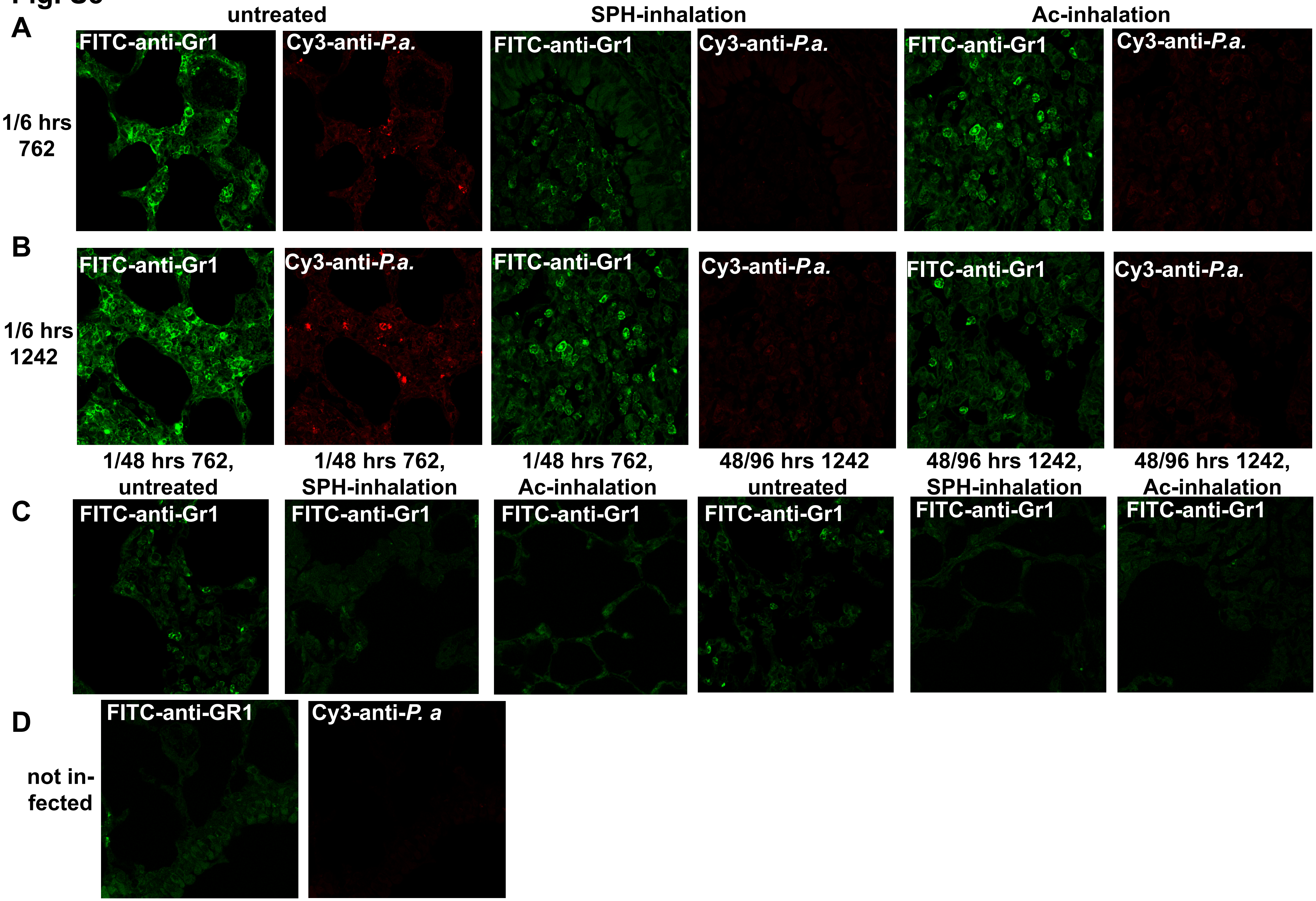

A
untreated
SPH-inhalation
Ac-inhalation
1/6 hrs
762
FITC-anti-Gr1
Cy3-anti-P.a.
B
1/6 hrs
1242
1/48 hrs 762, untreated
1/48 hrs 762, SPH-inhalation
1/48 hrs 762, Ac-inhalation
48/96 hrs 1242 untreated
48/96 hrs 1242, SPH-inhalation
48/96 hrs 1242, Ac-inhalation
C
D
not in-fected
FITC-anti-GR1
Cy3-anti-P. a

Fig. S6

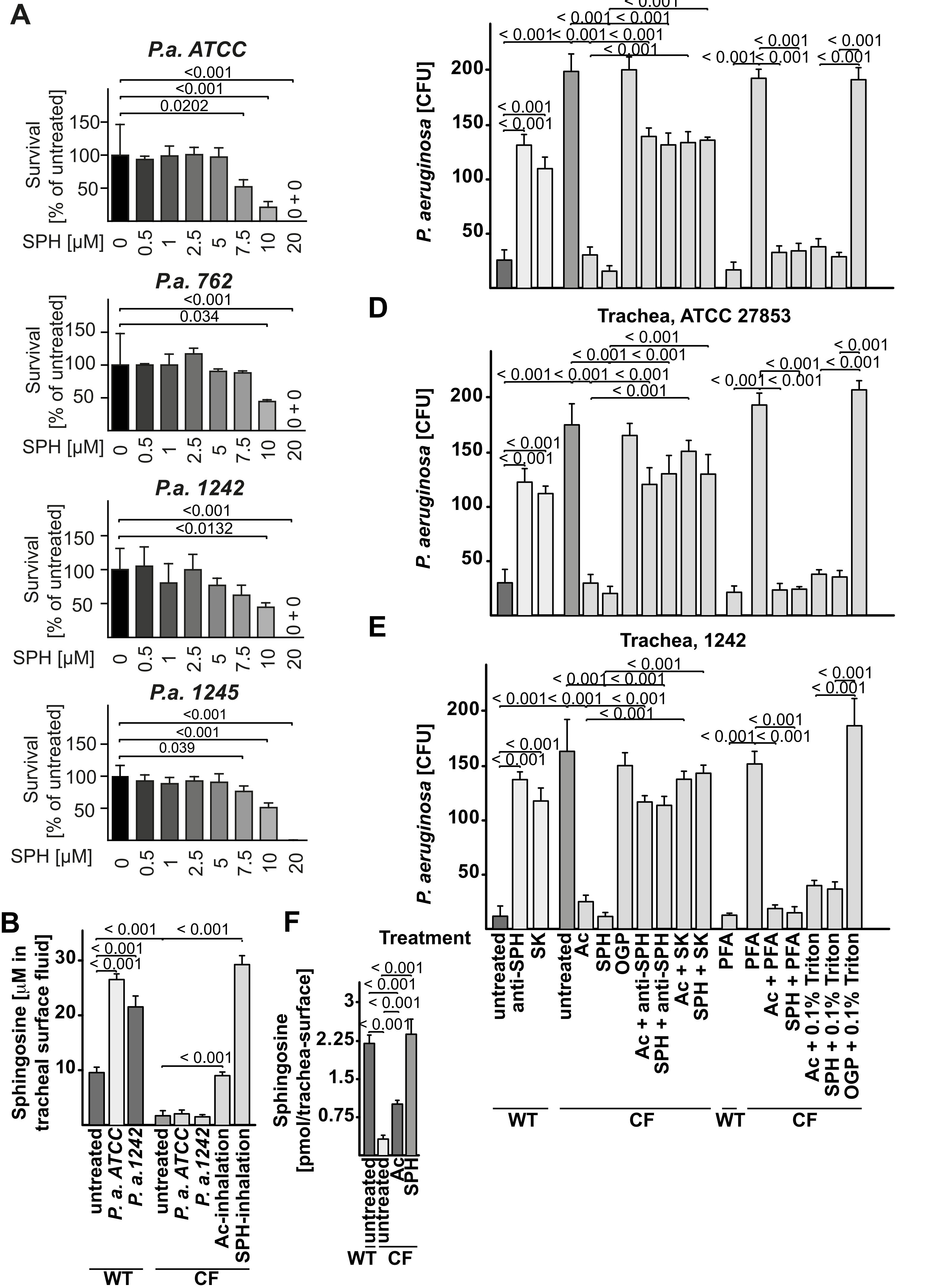
A
P.a. ATCC
Survival [% of untreated]
SPH [µM]
0 0.5 1 2.5 5 7.5 10 20
<0.001
<0.001
0.0202
0 + 0
P.a. 762
<0.001
0.034
P.a. 1242
<0.001
<0.0132
P.a. 1245
<0.001
<0.001
0.039
B
Sphingosine [µM in tracheal surface fluid]
untreated
P. a. ATCC
P. a.1242
untreated
P. a. ATCC
P. a.1242
Ac-inhalation
SPH-inhalation
WT
CF
< 0.001
C
Trachea, 762
P. aeruginosa [CFU]
D
Trachea, ATCC 27853
E
Trachea, 1242
Treatment
untreated
anti-SPH
SK
untreated
Ac
SPH
OGP
Ac + anti-SPH
SPH + anti-SPH
Ac + SK
SPH + SK
PFA
PFA
Ac + PFA
SPH + PFA
Ac + 0.1% Triton
SPH + 0.1% Triton
OGP + 0.1% Triton
WT
CF
WT
CF
F
Sphingosine [pmol/trachea-surface]
untreated
untreated
Ac
SPH
WT
CF
< 0.001

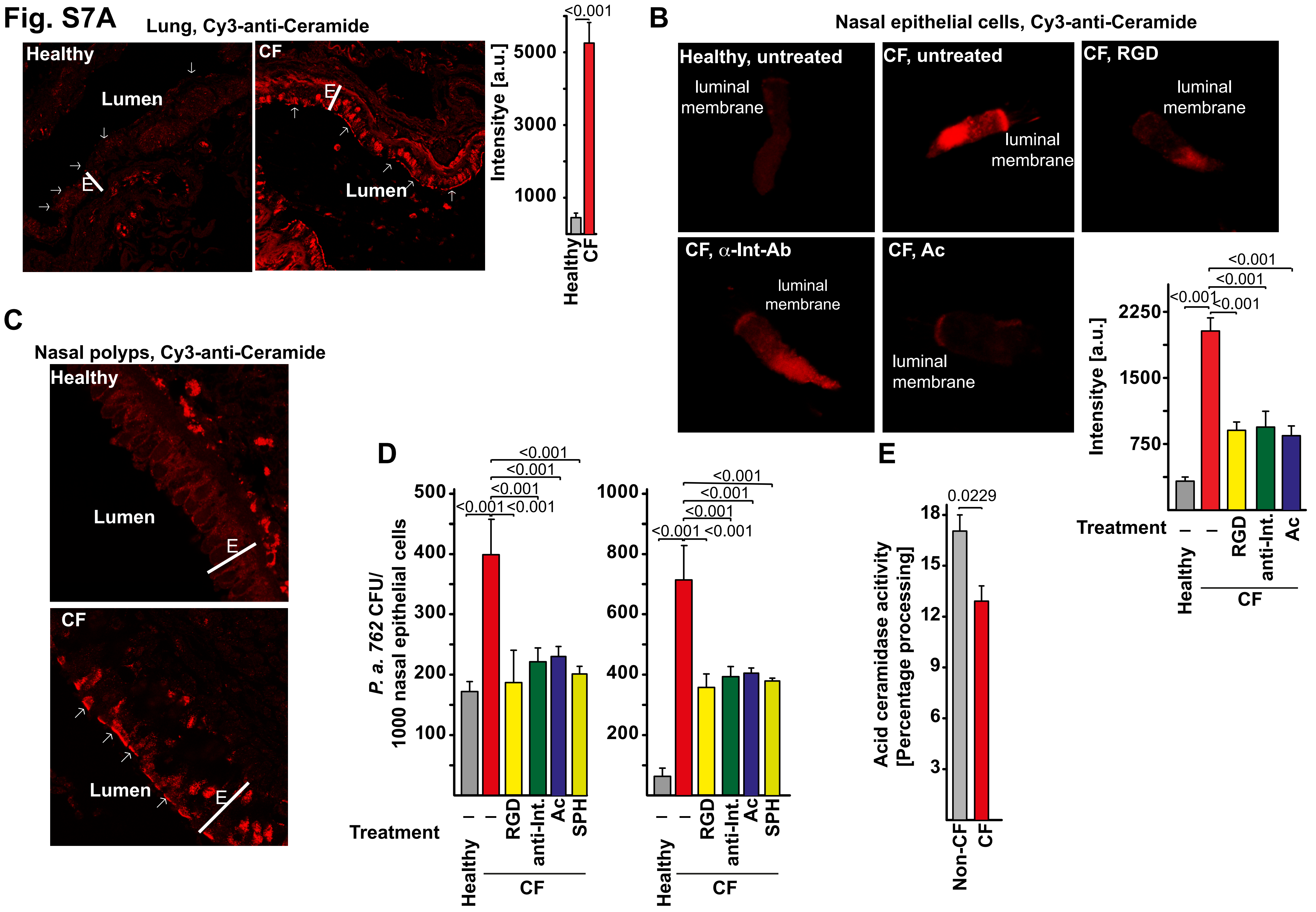

Fig. S7A
Lung, Cy3-anti-Ceramide
Healthy
Lumen
E
CF
Lumen
<0.001
Intensitye [a.u.]
5000
3000
1000
Healthy
CF
B
Nasal epithelial cells, Cy3-anti-Ceramide
Healthy, untreated
luminal membrane
CF, untreated
luminal membrane
CF, RGD
luminal membrane
CF, α-Int-Ab
luminal membrane
CF, Ac
luminal membrane
<0.001
<0.001
<0.001
<0.001
Intensitye [a.u.]
2250
1500
750
Treatment
Healthy
RGD
anti-Int.
Ac
CF
C
Nasal polyps, Cy3-anti-Ceramide
Healthy
Lumen
E
CF
Lumen
E
D
<0.001
<0.001
<0.001
<0.001 <0.001
500
400
300
200
100
P. a. 762 CFU/ 1000 nasal epithelial cells
Treatment
Healthy
RGD
anti-Int.
Ac
SPH
CF
<0.001
<0.001
<0.001
<0.001 <0.001
1000
800
600
400
200
Healthy
RGD
anti-Int.
Ac
SPH
CF
E
0.0229
18
15
12
9
6
3
Acid ceramidase acitivity [Percentage processing]
Non-CF
CF
