# Supplementary material for: β1-Integrin Accumulates in Cystic Fibrosis Luminal Airway Epithelial Membranes and Decreases Sphingosine, Promoting Bacterial Infections
Source: Cell Host Microbe. 2017 Jun 14;21(6):707–718.e8. doi: 10.1016/j.chom.2017.05.001 (PMC5475347; doi:10.1016/j.chom.2017.05.001)
Supplement: Document S2. Article plus Supplemental Information [file mmc2.pdf]

Article

Cell Host & Microbe

β1-Integrin Accumulates in Cystic Fibrosis Luminal Airway Epithelial Membranes and Decreases Sphingosine, Promoting Bacterial Infections

Graphical Abstract

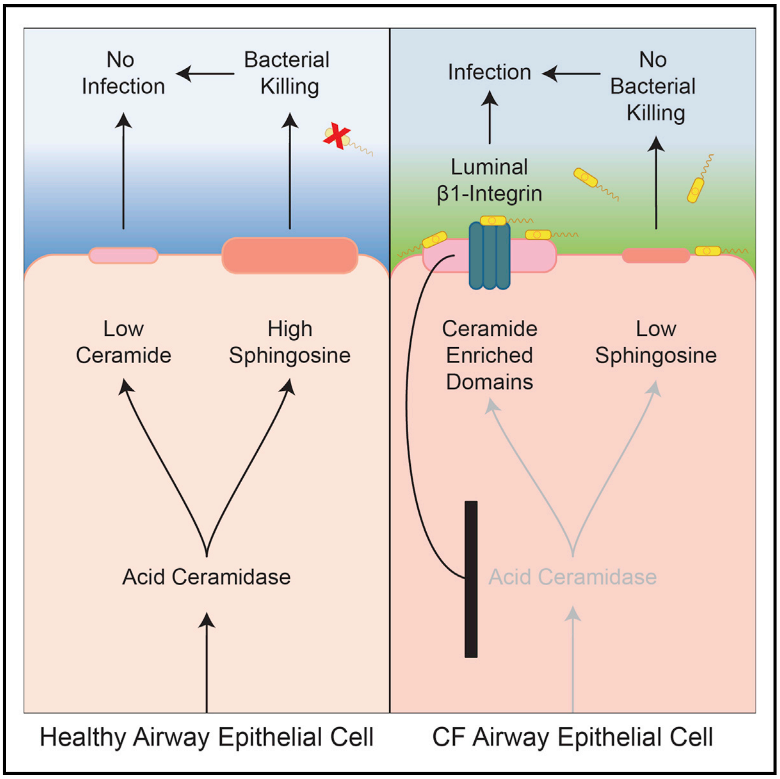

Highlights

- Cystic fibrosis (CF) airway cells ectopically express luminal membrane β1-integrins (β1)
- β1 decreases acid ceramidase (Ac), increasing ceramide and additional β1 accumulation
- Low Ac activity reduces sphingosine (Sp) levels, thereby impairing bacterial clearance
- Normalizing β1, ceramide, or Sp prevents bacterial infection in CF cells and mice

Authors

Heike Grassmé, Brian Henry, Regan Ziobro, ..., Michael J. Edwards, Malcolm Brodlie, Erich Gulbins

Correspondence

erich.gulbins@uni-due.de

In Brief

Bacterial pneumonia is a major clinical problem in cystic fibrosis (CF) patients. Grassmé et al. show that luminal β1-integrin accumulation, acid ceramidase downregulation, increased ceramide, and the resulting sphingosine depletion in CF epithelial membranes allows pulmonary *P. aeruginosa* infections. Normalizing β1-integrin prevents bacterial infection in CF cells and mice.

CrossMark

Grassmé et al., 2017, Cell Host & Microbe *21*, 707–718
June 14, 2017 © 2017 The Authors. Published by Elsevier Inc.
http://dx.doi.org/10.1016/j.chom.2017.05.001

CellPress

CellPress

β1-Integrin Accumulates in Cystic Fibrosis Luminal Airway Epithelial Membranes and Decreases Sphingosine, Promoting Bacterial Infections

Heike Grassmé,[1,9] Brian Henry,[1,2,9] Regan Ziobro,[1,9] Katrin Anne Becker,[1,9] Joachim Riethmüller,[3,9] Aaron Gardner,[4] Aaron P. Seitz,[2] Joerg Steinmann,[5] Stephan Lang,[6] Christopher Ward,[4] Edward H. Schuchman,[7] Charles C. Caldwell,[2] Markus Kamler,[8] Michael J. Edwards,[2] Malcolm Brodlie,[4] and Erich Gulbins[1,2,10,*]

[1]Department of Molecular Biology, University of Duisburg-Essen, Hufelandstrasse 55, 45122 Essen, Germany
[2]Department of Surgery, University of Cincinnati, 231 Albert Sabin Way, ML 0558, Cincinnati, Ohio 45229, USA
[3]Center for Pediatric Clinical Studies, Children's Clinic, University of Tuebingen, Hoppe-Seyler-Strasse 1, 72076 Tübingen, Germany
[4]Institute of Cellular Medicine, Newcastle University, c/o Level 3, Clinical Resource Building, Great North Children's Hospital, Queen Victoria Road, Newcastle upon Tyne, NE1 4LP, UK
[5]Department of Medical Microbiology, University of Duisburg-Essen, Hufelandstrasse 55, 45122 Essen, Germany
[6]Department of Otorhinolaryngology, University of Duisburg-Essen, Hufelandstrasse 55, 45122 Essen, Germany
[7]Department of Genetics & Genomic Sciences, Icahn School of Medicine at Mount Sinai, 1425 Madison Avenue, New York, NY 10029, USA
[8]West German Heart and Vascular Center Essen, University of Duisburg-Essen, Hufelandstrasse 55, 45122 Essen, Germany
[9]These authors contributed equally
[10]Lead Contact
*Correspondence: erich.gulbins@uni-due.de
http://dx.doi.org/10.1016/j.chom.2017.05.001

SUMMARY

Chronic pulmonary colonization with bacterial pathogens, particularly *Pseudomonas aeruginosa,* is the primary cause of morbidity and mortality in patients with cystic fibrosis (CF). We observed that β1-integrins accumulate on the luminal membrane of upper-airway epithelial cells from mice and humans with CF. β1-integrin accumulation is due to increased ceramide and the formation of ceramide platforms that trap β1-integrins on the luminal pole of bronchial epithelial cells. β1-integrins downregulate acid ceramidase expression, resulting in further accumulation of ceramide and consequent reduction of surface sphingosine, a lipid that kills bacteria. Interrupting this vicious cycle by triggering surface β1-integrin internalization via anti-β1-integrin antibodies or the RGD peptide ligand—or by genetic or pharmacological correction of ceramide levels—normalizes β1-integrin distribution and sphingosine levels in CF epithelial cells and prevents *P. aeruginosa* infection in CF mice. These findings suggest a therapeutic avenue to ameliorate CF-associated bacterial infections.

INTRODUCTION

Cystic fibrosis (CF) is the most common autosomal recessive disorder in the European Union (EU) and the United States (USA), affecting one of every 2,500 children born in Western countries. It is caused by mutations of the CF transmembrane conductance regulator gene (human: *CFTR*, mouse: *Cftr*). Although genetic mutations in *CFTR* lead to several respiratory, reproductive, and gastrointestinal complications, the primary cause of morbidity and mortality for these patients is the destructive effects of chronic pulmonary colonization with bacterial pathogens—in particular, *Pseudomonas aeruginosa*. Approximately 80% of CF patients will host *P. aeruginosa* by the age of 25 (CF foundation, 2010). In addition to their increased susceptibility to *P. aeruginosa*, CF lungs are characterized by chronic inflammation and progressive fibrosis (Elborn, 2016). At present, the molecular mechanisms that mediate all three hallmarks of the disease—i.e., infection, inflammation, and fibrosis—require further definition.

Because CFTR exhibits chloride-channel activity, it has been speculated that water absorption by the mucus present on the epithelial cells of the respiratory tract may be altered and that this alteration may result in reductions in mucociliary clearance, a reduced function of defensins, and the ability to eliminate *P. aeruginosa* (Matsui et al., 1998). The increased viscosity of the mucus may also affect the ability of neutrophils to migrate to and kill bacteria in the respiratory tract (Matsui et al., 2005). However, although in vitro experiments suggested the concept of reduced mucociliary clearance in CF, it was difficult to prove in vivo (Locke et al., 2016). Recent studies have suggested that one of the leading causes of bacterial infections in CF patients is an imbalance between pro-inflammatory and anti-inflammatory cytokines in the airways (for review, see Elborn, 2016). At present, the mechanisms leading to inflammation in CF are unknown.

We recently reported that, in vitro and in vivo, the lipid sphingosine efficiently kills many bacterial species, including *P. aeruginosa*, *Staphylococcus aureus* (even MRSA), and *Acinetobacter baumanii* (Pewzner-Jung et al., 2014; Tavakoli Tabazavareh et al., 2016). We found that sphingosine is abundantly expressed on the luminal surface of human nasal epithelial cells obtained from healthy persons and from the trachea and

CrossMark

© 2017 The Authors. Published by Elsevier Inc.
This is an open access article under the CC BY license (http://creativecommons.org/licenses/by/4.0/).

conducting bronchi of wild-type (WT) mice, whereas it is almost undetectable on the surface of nasal epithelial cells from individuals with CF and on tracheal and bronchial cells from CF mice. Inhalation of sphingosine by CF mice eliminated existing *P. aeruginosa* infections and prevented new *P. aeruginosa* or *S. aureus* infections in these mice (Pewzner-Jung et al., 2014; Tavakoli Tabazavareh et al., 2016), a finding demonstrating that sphingosine plays a key role in the innate and immediate defense of the upper respiratory tract. Why sphingosine levels are lower in CF epithelial cells than in healthy cells is presently unknown.

In contrast, ceramide levels have been shown to be higher in CF epithelial cells, and pharmacologic or genetic normalization of ceramide prevents *P. aeruginosa* infection in CF mice (Teichgräber et al., 2008; Zhang et al., 2010; Becker et al., 2010; Brodlie et al., 2010a; Ulrich et al., 2010; Bodas et al., 2011). Ceramide molecules form small domains in the plasma membrane; these domains are resolute lipid platforms in an otherwise dynamic membrane environment and serve to sequester proteins such as cell-surface molecules (Grassmé et al., 2001, 2002; Nurminen et al., 2002).

Therefore, we investigated whether ceramide-enriched membrane domains in CF cells mediate an ectopic expression and function of proteins in CF cells and whether these proteins regulate ceramide levels in a vicious cycle, simultaneously controlling the surface levels of sphingosine in CF epithelia and thereby also determining infection susceptibility of CF mice and patients.

Here we report that β1-integrins are ectopically expressed on the luminal pole of CF bronchial, tracheal, and nasal epithelial cells of individuals and mice with CF but are absent from such cells in healthy individuals and WT mice. The trapping of β1-integrins in the luminal membrane of CF bronchial, tracheal, and nasal epithelial cells is mediated by the accumulation of ceramide in CF cells. Ectopic β1-integrins in the luminal membrane downregulate the expression of acid ceramidase (Ac) in human and murine CF airway epithelial cells and thereby mediate a further accumulation of ceramide and a concomitant depletion of sphingosine. The vicious cycle between ceramide and β1-integrin can be blocked by the inhalation of β1-integrin ligands, which force internalization of β1-integrin and thereby normalize its surface expression, or by the reduction of ceramide levels. Blocking this vicious cycle normalizes sphingosine levels and prevents *P. aeruginosa* infection of airway epithelial cells from individuals with CF or pneumonia of CF mice, respectively.

RESULTS

β1-Integrins Are Ectopically Expressed on the Luminal Pole of Cystic Fibrosis Cells

Previous studies of the cellular distribution of β1-integrins in epithelial cells demonstrated that, after synthesis, β1-integrins are transported to the basolateral and luminal membranes (Gut et al., 1998). Although they are stably integrated into the basolateral membrane, these integrins are rapidly internalized from the luminal membrane and degraded, resulting in the absence of β1-integrins from the luminal membrane (Gut et al., 1998). Our confocal microscopy studies using the monoclonal anti-β1-integrin MB1.2 antibody on paraffin-embedded sections from the lungs of WT mice confirm the absence of β1-integrins on the luminal surface of WT bronchial epithelial cells (Figures 1A and S1A). In contrast, we observed a marked luminal presentation of β1-integrins on bronchial epithelial cells in CF^{MHH} (Figures 1A and S1A) or *Cftr*$^{-/-}$ mice (not shown). Here, and in all studies below, we used two CF mouse strains—i.e., CF^{MHH} mice, which have a residual activity of Cftr, and *Cftr*$^{-/-}$ mice, which lack Cftr in the lung but express a CFTR transgene in the gut under control of the fatty-acid-binding protein promoter. Both strains can be fed with a normal diet and do not show any developmental retardation. In our studies, we did not notice any differences between the two strains as detailed below.

Ectopic luminal expression of β1-integrins on bronchial epithelial cells in CF lungs in vivo was confirmed by intratracheal injection of the anti-β1-integrin antibody 9EG7 into the lungs of Cftr$^{-/-}$ mice, followed by lavage, in situ fixation, and staining with Cy3-coupled anti-rat antibodies, which bind to the anti-β1-integrin antibodies already present in the airways (Figures 1B and S1B). Similar results were obtained with CF^{MHH} mice. Because the 9EG7 monoclonal antibody binds to the active conformation of β1-integrin (Lenter et al., 1993), these findings suggest that the ectopically expressed β1-integrin is active either in the extended closed or open state. We also confirmed surface expression of β1-integrin by immunoprecipitating surface β1-integrin from the intact surface of CF tracheae with anti-β1-integrin antibodies (clone MB1.2) that detect all conformations of β1-integrin (Figures 1C and S1C).

Control stainings with rat isotype control antibodies to the β1-integrin antibodies (Figures S1A and S1B) or just with Cy3-coupled anti-rat antibodies (not shown) confirmed the specificity of the stainings. Quantification of four independent immunoprecipitation experiments confirms the fluorescence microscopy data (Figure S1C). Further controls stained with Cy3-coupled Annexin V to detect phosphatidylserine in the membrane revealed no difference in the intensity of the stainings between CF and WT mice (Figure S1D). The ratio of the fluorescence intensities of surface β1-integrin and Cy3 annexin in CF and WT airways (Figure S1E) confirms the notion of accumulation of β1-integrin in the luminal membrane of bronchial and tracheal epithelial cells of CF mice. Further, we stained WT and CF lung sections with anti-β2-integrin antibodies and did not detect a difference between WT and CF airways (Figure S1F). The anatomical structures are displayed in Figure S1G. In summary, these experiments demonstrate that β1-integrins are ectopically expressed on the luminal membrane of tracheal and bronchial epithelial cells from CF mice in vivo.

Ceramide Mediates Ectopic Expression of β1-Integrins in Cystic Fibrosis Cells

Next, we investigated whether ceramide mediates the ectopic expression of β1-integrins and whether normalization of ceramide concentrations in CF mice alters the surface expression of β1-integrins. We have previously shown that ceramide clusters and activates β1-integrins in B16F10 melanoma cells (Carpinteiro et al., 2015). We confirmed previous findings (Teichgräber et al., 2008) of high ceramide expression in bronchi of *Cftr*$^{-/-}$ mice (Figures 2A and S1H) or CF^{MHH} mice (not shown). Reducing acid sphingomyelinase (Asm) activity by treating *Cftr*$^{-/-}$ mice with amitriptyline or fluoxetine, which are

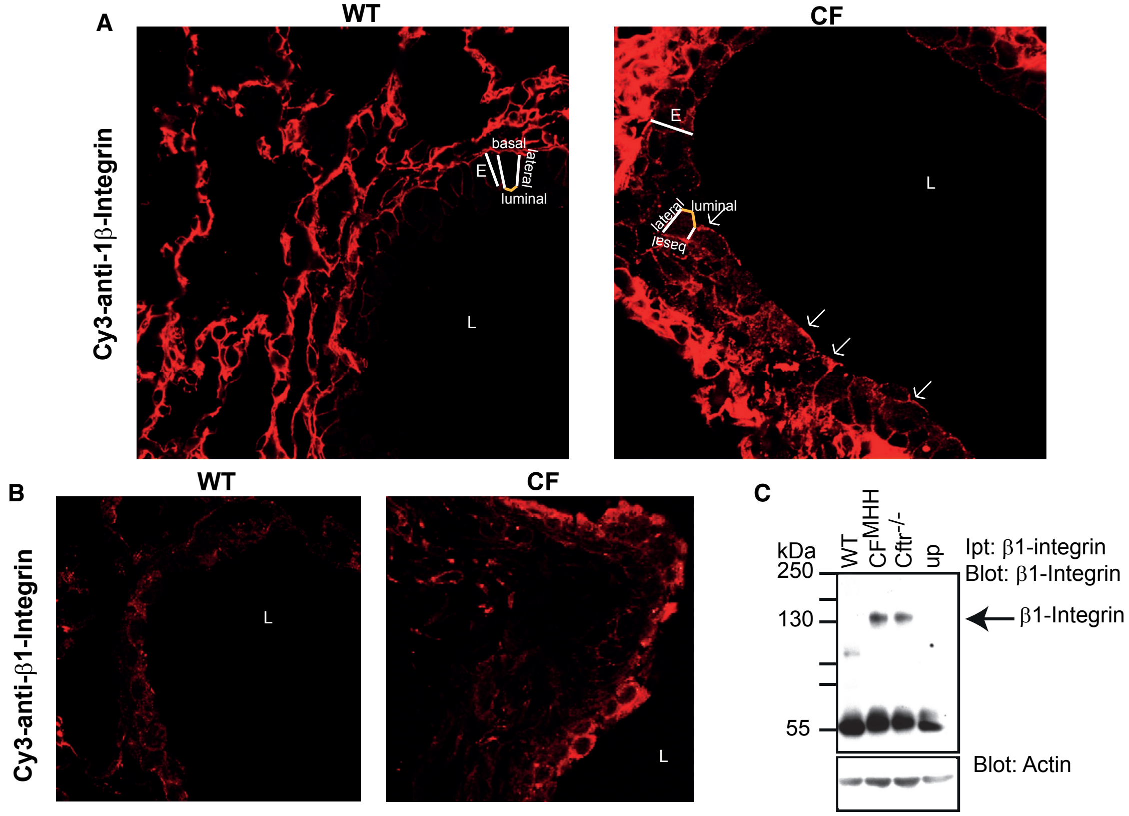

Figure 1. Cystic Fibrosis Bronchial Epithelial Cells Ectopically Express Luminal β1-Integrin

(A) Murine cystic fibrosis (CF) bronchial epithelial cells ectopically present β1-integrin on their luminal pole, whereas β1-integrin is absent from wild-type (WT) bronchi. Luminal integrins are indicated by arrows; E, bronchial epithelial cell layer; L, bronchial lumen. Paraffin-embedded sections from WT and CF^{MHH} lungs were stained with Cy3-coupled anti-β1-integrin antibodies and analyzed by confocal microscopy. The topology of the epithelial cell layer is illustrated on one cell (luminal surface in yellow). Representative results from six mice are shown.

(B) Intrapulmonary injection of anti-β1-integrin antibodies into $Cftr^{-/-}$ mice confirms luminal expression of β1-integrin in CF bronchial epithelial cells in vivo, whereas luminal β1-integrin is not expressed in WT bronchi. Representative results from six mice are shown.

(C) Immunoprecipitation of β1-integrin from the intact surface of the trachea reveals ectopic luminal expression of β1-integrin in the trachea of CF^{MHH} and $Cftr^{-/-}$ mice, but not in WT trachea. Up: unspecific immunoprecipitation with an irrelevant isotype control antibody. Results are representative of four similar experiments.

Please see also Figure S1.

pharmacological inhibitors of the acid sphingomyelinase and thereby reduce ceramide in CF tissues (Teichgräber et al., 2008; Becker et al., 2010; Hurwitz et al., 1994; Kornhuber et al., 2010), or by genetic heterozygosity of Asm normalizes ceramide levels (Figures 2A and S1H) as well as the subcellular distribution pattern of β1-integrins (Figures 2B and S1I) in murine CF bronchial epithelial cells. Ceramide has been previously shown to cluster cell-surface receptors, contributing to the amplification of signal transduction (Grassmé et al., 2001). Therefore, we investigated whether ceramide and β1-integrins co-cluster in CF mice. Confocal microscopy of bronchi from $Cftr^{-/-}$ mice (Figure 2C) or CF^{MHH} mice (not shown) demonstrated clustering of β1-integrin in luminally expressed ceramide-enriched membrane platforms. Collectively, these findings demonstrate that ceramide plays a crucial role in the aberrant luminal presentation of integrins on bronchial epithelial cells in CF mice.

To further investigate the role of ceramide in the ectopic luminal upregulation of β1-integrins by an experimental approach independent of *Cftr* deficiency, we used overexpressing Asm-transgenic and heterozygous Ac-deficient mice. These mice accumulate ceramide in the lungs, in particular in bronchial epithelial cells (Figure 2D), and show a distinctive increase in the expression of luminal β1-integrin in the bronchial epithelial cells of each genetic variant (Figure 2E), a finding indicating a direct and causal link between ceramide accumulation and the ectopic localization of luminal β1-integrin in the mouse lung. As is true in CF mice, the pharmacologic reduction of ceramide levels in Asm-transgenic and Ac-heterozygous mice upon treatment with amitriptyline normalized the expression of β1-integrin. The fluorescence studies were also quantified (Figures S1J and S2A).

Normalization of Luminal β1-Integrin Expression Also Normalizes Ceramide Levels in Cystic Fibrosis Airways

It has been previously shown that binding β1-integrins with antibodies or arginine-glycine-aspartate (RGD) peptides results in internalization of the integrin molecule (Tran Van Nhieu and Isberg, 1993; Ye et al., 2006). Thus, to achieve internalization of the ectopically expressed surface β1-integrins on CF airways,

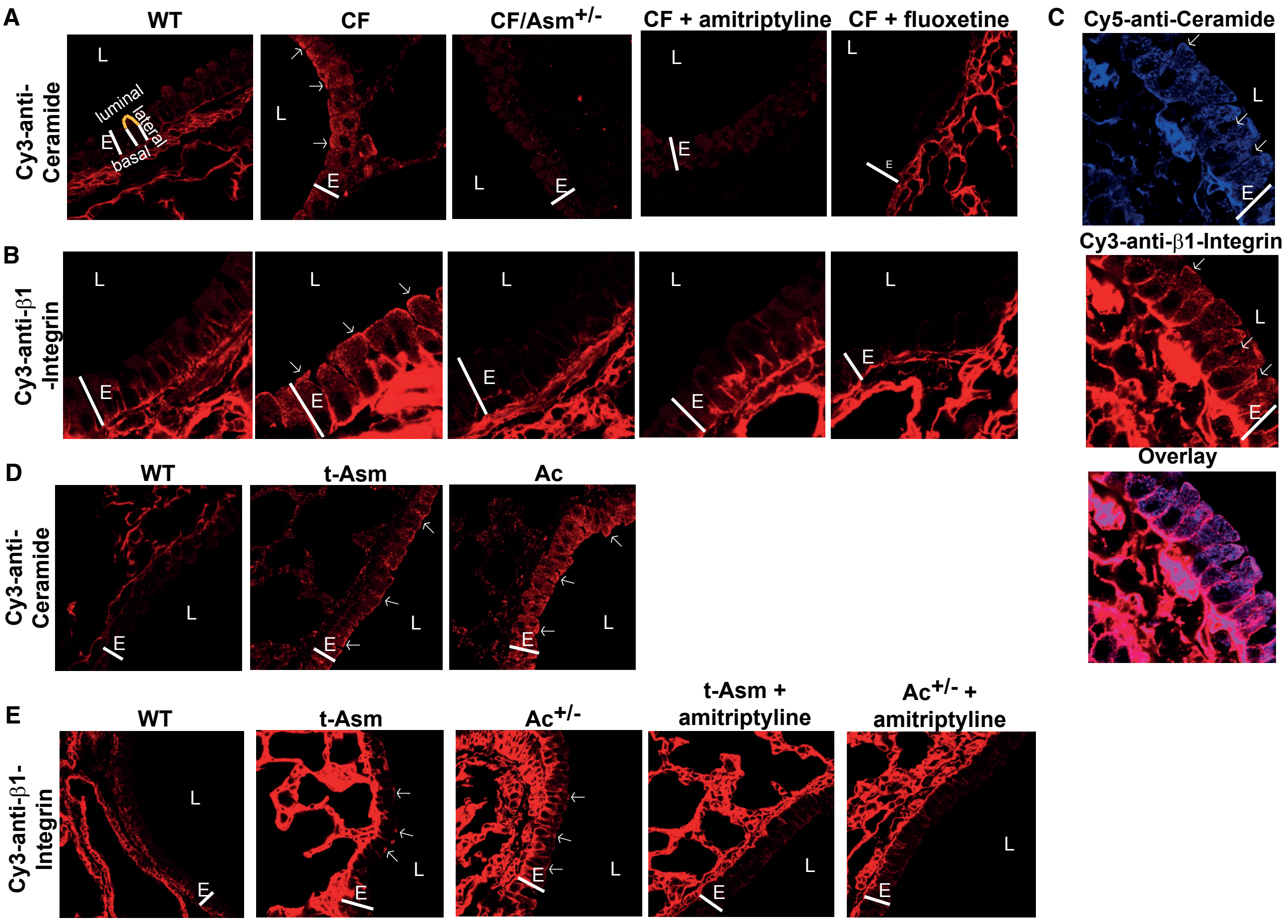

Figure 2. Luminal β1-Integrin Expression Is Caused by Accumulation of Cell-Membrane Ceramide

(A and B) Epithelial cells in bronchi of CF mouse (*Cftr*$^{-/-}$) lungs accumulate higher levels of ceramide than healthy human or WT mouse cells. Heterozygosity or pharmacological inhibition of acid sphingomyelinase (Asm) corrects ceramide accumulation in *Cftr*$^{-/-}$ bronchial epithelial cells (A) and ectopic expression of β1-integrin on the cell lumen (B). Lung sections were stained with Cy3-coupled anti-ceramide or anti-β1-integrin antibodies and analyzed by confocal microscopy. The topology of a representative cell is indicated (luminal surface in yellow).

(C) Lung sections from *Cftr*$^{-/-}$ mice stained with Cy5-coupled anti-ceramide and Cy3-labeled anti-β1-integrin antibodies demonstrate co-localization of ceramide and β1-integrin.

(D) Ceramide also accumulates in the lungs of acid sphingomyelinase (Asm)-transgenic and heterozygous acid ceramidase (Ac)-deficient mice. This accumulation of ceramide is associated with luminal expression of β1-integrin in both genetic variants. Pharmacologic reduction of Asm attenuates surface expression of β1-integrin in Asm-transgenic or Ac-heterozygous mice. Please see also Figures S1 and S2.

Results are representative of experiments using at least six mice per group in (A)–(C) or four mice per group in (D) and (E). E, epithelial cell layer; L, lumen. Arrows indicate ceramide-enriched membrane platforms in (A), (C), and (D) and indicate β1-integrin cluster in (B), (C), and (E). Please note that ceramide staining in the alveoli did not differ between WT and CF mice, consistent with the expression of CF. Not all samples show alveoli around the bronchi. In D (WT), the epithelial cell layer and a large blood vessel are shown, while the other photos show epithelial cells and alveoli.

we subjected *Cftr*$^{-/-}$ mice (Figures 3A–3C) or CF^{MHH} mice (Figure 3B) either to inhalation of nebulized anti-β1-integrin antibody clone 9EG7, which binds the partly or fully active conformation of β1-integrin molecules (Lenter et al., 1993), or to inhalation of RGD peptides, which bind to the fibronectin binding site of the receptor (D'Souza et al., 1988). Inhalation of 0.9% NaCl, isotype control antibodies or scrambled peptides served as controls.

Inhalation of RGD peptides and anti-β1-integrin antibodies (clone 9EG7) normalized tracheal and bronchial surface expression of β1-integrin, whereas inhalation of control reagents had no effect (Figures 3A, 3B, and S2B; Table S1). Inhalation of fluorescein isothiocyanate (FITC)-coupled RGD peptides confirmed the rapid uptake of β1-integrin into the cells upon ligand binding (Figure 3C). Normalization of luminal β1-integrin levels also normalized surface ceramide levels in the trachea and the bronchi of CF mice (Figures 3D, 3E, and S2B). These findings suggest a vicious cycle between surface ceramide and β1-integrin.

Ectopically Expressed β1-Integrin Downregulates Ac Expression

To test whether ectopic expression of β1-integrin regulates the lipid phenotype in CF airways, we measured the expression

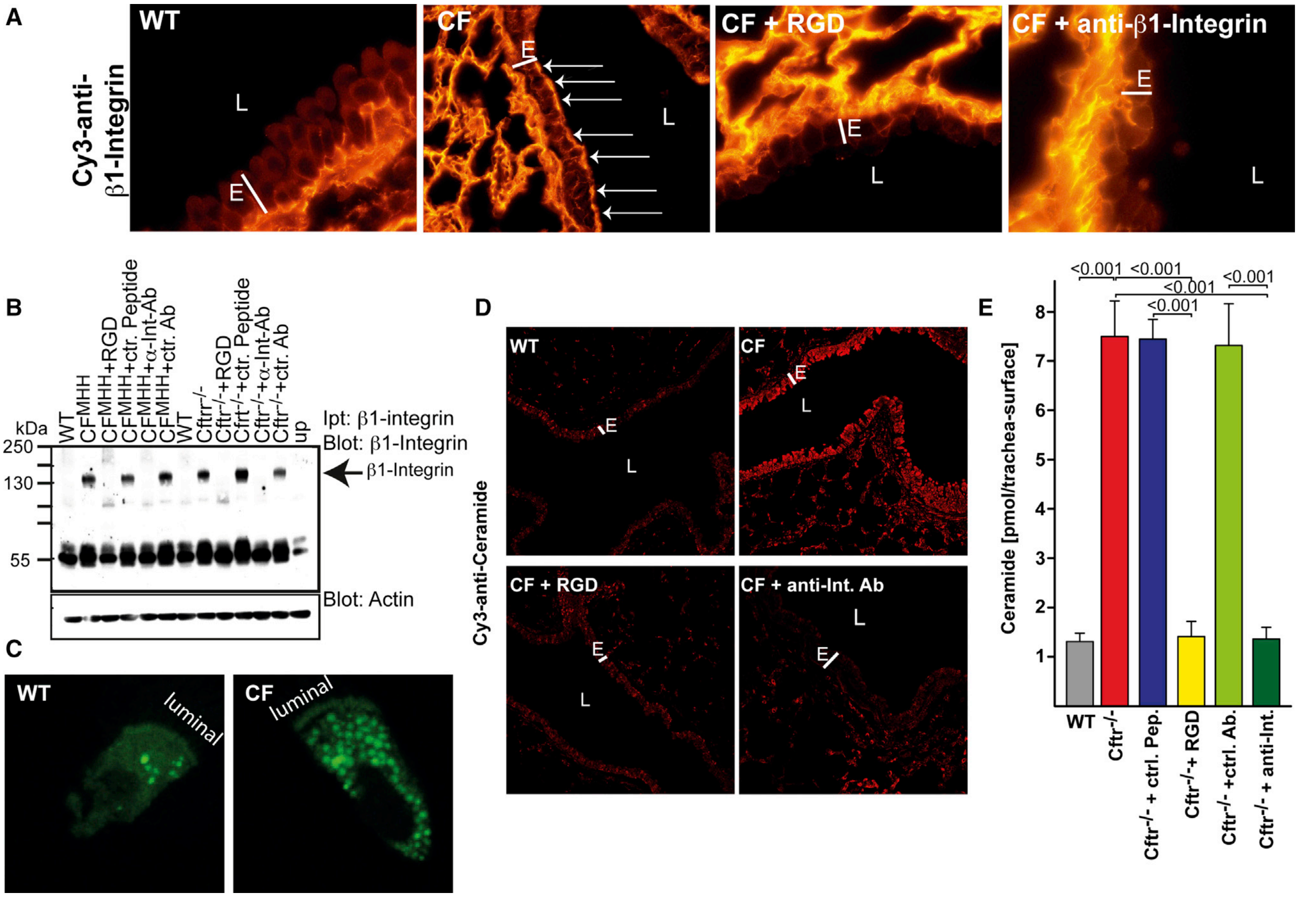

Figure 3. Normalization of β1-Integrin Expression and Ceramide Levels by Inhalation of Anti-β1-Integrin Antibodies or Arginine-Glycine-Aspartate Peptides

(A) Subjecting cystic fibrosis (*Cftr*$^{-/-}$) mice to inhalation of nebulized anti-β1-integrin 9EG7 antibodies or arginine-glycine-aspartate (RGD) peptides normalizes the expression of ectopic integrins in the trachea and bronchi of CF mice. Surface β1-integrin is indicated by arrows.

(B) Ectopic surface expression of β1-integrin and its downregulation upon inhalation of RGD peptides or anti-β1-integrin 9EG7 antibodies (α-Int-Ab), but not by the appropriate control antibodies (ctr. Ab.) or control peptides (ctr. peptide), was confirmed by immunoprecipitation of surface β1-integrin, as above.

(C) Inhaled fluorescein isothiocyanate (FITC)-conjugated RGD is rapidly internalized. WT or *Cftr*$^{-/-}$ (CF) mice were subjected to inhalation with FITC-conjugated RGD, and tracheal epithelial cells were isolated and analyzed by confocal microscopy.

(D and E) The expression levels of ceramide are normalized by inhalation of RGD peptides or anti-β1-integrin 9EG7 antibodies (anti-Int. Ab), as measured by immunostaining and confocal microscopy (D) or by in situ kinase assays measuring surface ceramide levels, while control (ctr.) peptides or antibodies were without effect (E).

E, bronchial epithelial cell layer; L, bronchial lumen. Shown are typical results from one of six independent studies (A–D). (E) shows the mean ± SD, n = 6, ANOVA, overall $p < 0.001$. Please see also Figures S2 and S3 and Table S1.

and activity of Ac in CF airways before or after treatment with either RGD peptides or anti-β1-integrin antibodies.

Fluorescence microscopy, western blot studies, and in situ activity measurements of Ac in the trachea showed largely reduced expression of Ac in trachea and large bronchi of *Cftr*$^{-/-}$ mice, in particular in the cilia and the luminal membrane of epithelial cells (Figures 4A–4C and S2C; Table S2). Inhalation of RGD peptides or anti-β1-integrin antibodies normalized the expression of Ac (Figures 4A–4C and S2C; Table S2). In accordance with these findings, the activity of Ac, as measured in vivo on the surface of the trachea, was greatly reduced in *Cftr*$^{-/-}$ mice and was only slightly further reduced by incubating the trachea with Ac inhibitors, which reduced the activity of Ac in WT trachea to the level of that observed in *Cftr*$^{-/-}$ mice (Figure 4D). Finally, inhalation of RGD peptides or anti-β1-integrin antibodies normalized the levels of sphingosine in CF airways (Figures 4E, 4F, and S2D). These findings suggest that ectopically expressed β1-integrin regulates ceramide and sphingosine by downregulating Ac levels in CF cells.

Further studies (Figures S2E–S2G, S3A–S3H, S4A, and S4B) addressed mechanisms of ceramide-mediated trapping of β1-integrin and show (1) that internalization of a GPI-linked protein, Art1, is not sufficient to trigger internalization of β1-integrin and does not alter ceramide and sphingosine concentrations in CF cells (Figures S2E–S2G); (2) that ceramide itself prevents rapid degradation and internalization of β1-integrin (Figures S3A and S3B; Tables S3 and S4); (3) that infection with *P. aeruginosa* activates the acid sphingomyelinase but has almost no impact on

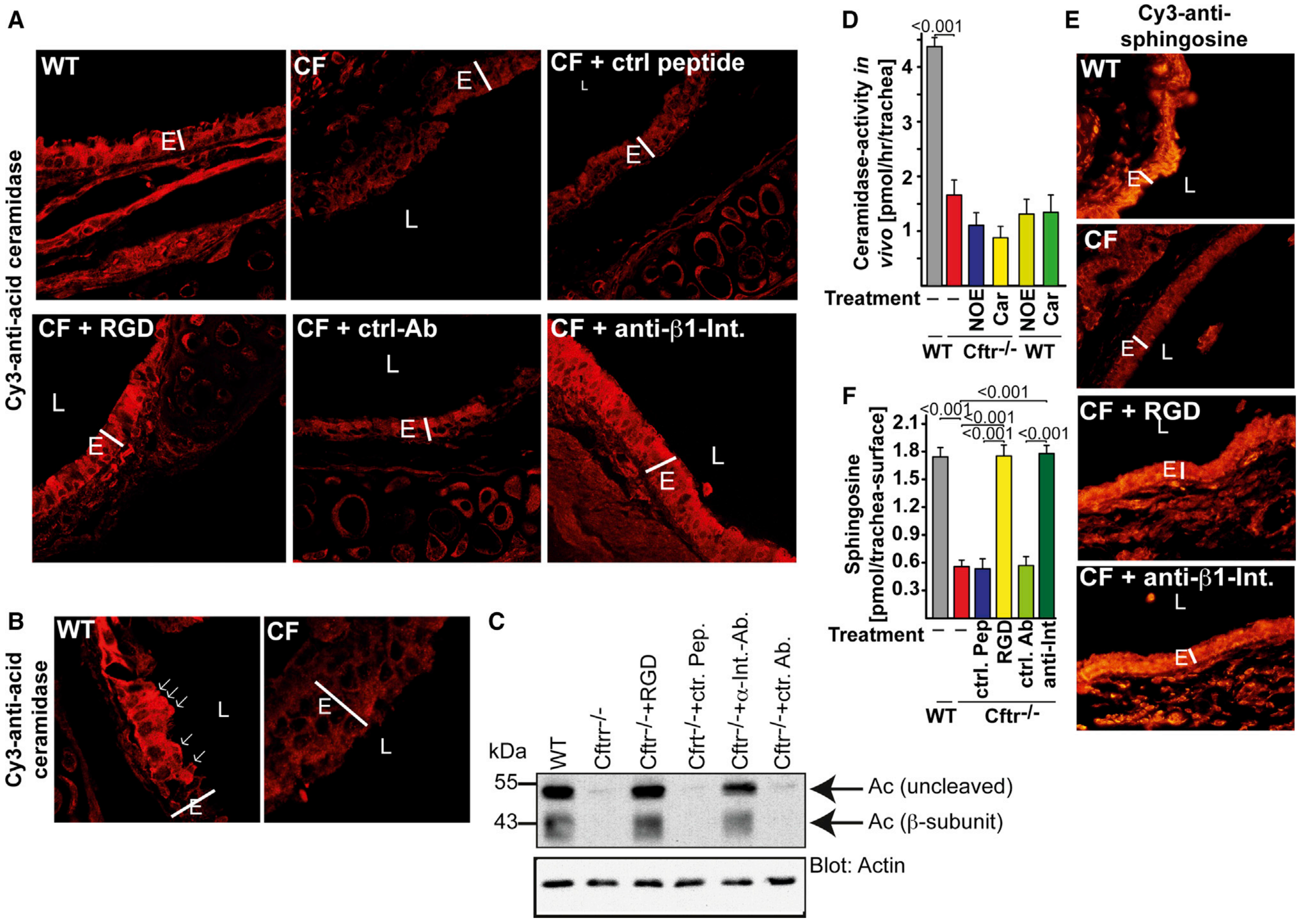

Figure 4. Expression of Acid Ceramidase Is Reduced in Cystic Fibrosis Mice and Is Corrected by Normalization of β1-Integrin Expression

(A) Mice were subjected to inhalation procedure as indicated. The trachea was removed after 2 hr, fixed, embedded in paraffin, sectioned, dewaxed, and stained with Cy3-coupled anti-acid ceramidase (Ac) antibodies. Expression of Ac is much lower in cystic fibrosis (CF) cells (*Cftr*$^{-/-}$) than in WT cells and is corrected by inhalation of either arginine-glycine-aspartate (RGD) peptides or anti-β1-integrin (Int) antibody 9EG7 (anti-Int), but is left unaltered by inhalation of control (ctr) peptides (pep) or antibodies (Ab). Figures are representative of six independent studies with similar results.

(B) Greater magnification shows luminal expression of Ac in epithelial cells of WT mice and absence of Ac in *Cftr*$^{-/-}$ cells. Arrows indicate areas with high expression of Ac.

(C) WT or *Cftr*$^{-/-}$ mice were left untreated or subjected to inhalation of RGD peptides, control (ctr) peptides, anti-β1-integrin, or isotype control antibodies. Tracheal epithelial cells were isolated and subjected to western blot analysis. These studies demonstrate a marked downregulation of Ac in CF cells; this is corrected after binding and normalization of β1-integrin surface expression. Figures are representative of four independent studies with similar results.

(D) Surface activity of Ac in the trachea was measured by injection of 4 μL [^{14}C16]-ceramide into the lumen of the trachea of anesthetized mice in the presence or absence of an Ac inhibitor—either N-oleoylethanolamine (NOE) or carmofur (Car). Mean ± SD, n = 4, *$p < 0.05$; t test.

(E) Sphingosine levels are markedly reduced in CF airways. Inhalation of nebulized anti-β1-integrin antibodies or RGD peptides normalizes the expression of surface sphingosine in the trachea and bronchi of CF mice. Sphingosine was visualized by staining with Cy3-coupled anti-sphingosine antibodies. The specificity of the anti-sphingosine antibodies had been previously confirmed (Pewzner-Jung et al., 2014). Shown are representative results from six independent experiments.

(F) Surface sphingosine levels were quantified on the luminal membrane of the trachea by in situ kinase assays. Means ± SD, n = 6, ANOVA, overall $p < 0.001$. E, bronchial epithelial cell layer; L, bronchial lumen. Please see also Figures S3 and S4 and Tables S3 and S4.

surface β1-integrin (Figures S3C and S3D); (4) that binding of a ligand to β1-integrin is sufficient to mediate its internalization, and is followed by re-expression of acid ceramidase and normalization of surface ceramide levels (Figures S3E–S3H); (5) that internalization of β1-integrin is required to trigger these events (Figures S3E–S3H); and (6) that β1-integrin physically interacts with ceramide-enriched membrane platforms (Figures S4A and S4B).

Binding and Internalization of Luminal β1-Integrins Prevent *P. aeruginosa* Infection in Cystic Fibrosis Mice

CF mice (CF^{MHH} and *Cftr*$^{-/-}$), overexpressing Asm-transgenic mice, and heterozygous Ac-deficient mice were all very suceptible to acute or chronic infection with non-mucoid and mucoid *P. aeruginosa* (Figures 5A–5D and Figures S4C–S4E). Inhalation of anti-β1-integrin antibodies or RGD peptides rescued each mouse strain from infection with *P. aeruginosa* (Figures 5A–5D

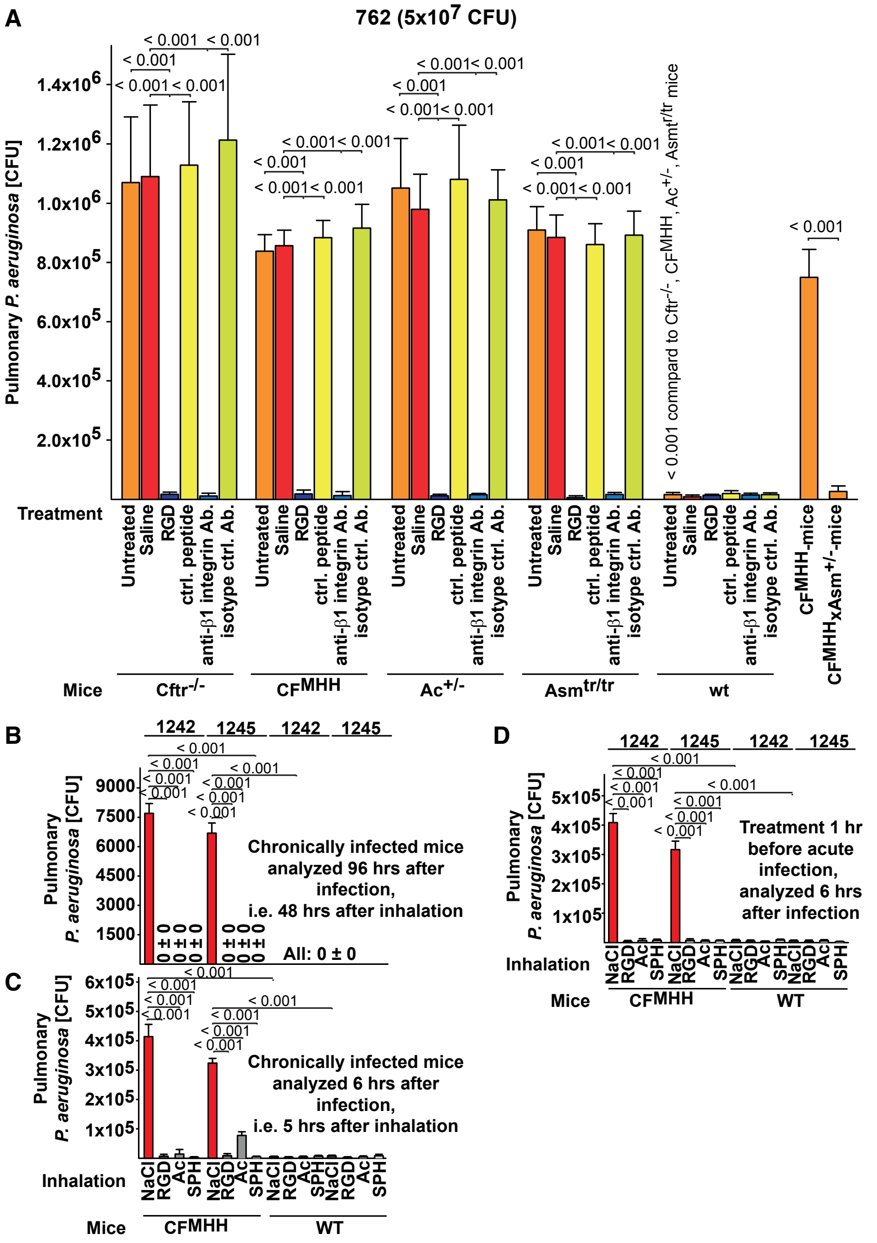

Figure 5. Inhalation of Anti-β1-Integrin Antibodies or RGD Peptides Prevents *Pseudomonas aeruginosa* Infection in Cystic Fibrosis Mice In Vivo

(A–D) Inhalation of either nebulized anti-β1-integrin antibodies (Ab) or RGD peptides or Asm-heterozygosity prevents pulmonary infection in cystic fibrosis (CF), acid sphingomyelinase (Asm)-transgenic, or heterozygous acid ceramidase (Ac)-deficient mice (A). (Sub-)chronic or acute infection of CF mice with mucoid *P. aeruginosa* strains 1242 or 1245 is eliminated by inhalation of Ac, sphingosine (SPH) or RGD-peptides. Mice were intranasally infected with 5×10^7 CFU of *P. aeruginosa* strain 762 (A), five times every 24 hr with 1×10^7 CFU of *P. aeruginosa* strains 1242 or 1245 (B and C) or once with 5×10^7 CFU of *P. aeruginosa* strains 1242 or 1245 (D). Anti-β1-integrin antibodies, Ac, SPH, or RGD peptides were inhaled 1 hr prior to infection with *P. aeruginosa* strain 762 (A) or either 1 hr or 48 hr after the last or 1 hr prior to infection with 1242 or 1245 (B–D). Pulmonary CFUs were determined 4 hr after infection (A) or either 6 hr or 96 hr after infection (B–D). Inhalation of 0.9% NaCl, control peptides, or isotype-matched antibodies (Ab) did not alter the infection with *P. aeruginosa* compared to untreated mice. Mean ± SD, n = 6, ANOVA, overall p < 0.001. Please see also Figures S4, S5, and S6.

and S4C–S4E). Heterozygosity of the acid sphingomyelinase in CF mice normalized the infection susceptibility of these mice, confirming previous findings (Figures 5A and S4C; Teichgräber et al., 2008). Control peptides or isotype-matched irrelevant antibodies did not alter the massive infection of these mouse strains.

Incubation of *P. aeruginosa* with anti-β1-integrin antibodies or RGD peptides prior to intranasal application did not alter the infection (data not shown); this finding excludes the possibility that the protective effect of these treatments is due to direct binding of the agents to bacterial proteins. Inhalation of sphingosine, Ac, or RGD also cured or prevented, respectively, (sub-)chronic or acute infections with two mucoid *P. aeruginosa* strains, 1242 and 1245 (Figures 5B–5D). Inhalation of sphingosine, Ac, or RGD peptides 1 hr after the infection also prevented mortality of CF mice pulmonarily infected with *P. aeruginosa* strains 762, ATCC 27853, 1242, or 1245 (Figure S4F). Conversely, the inhalation of anti-sphingosine antibodies by WT mice greatly sensitized them to *P. aeruginosa* infection (Figure S4G).

Histology studies confirm killing of *P. aeruginosa* strains 762, ATCC 27853, 1242, and 1245 upon inhalation of RGD-peptides, anti-β1-integrin antibodies, sphingosine, or acid ceramidase; they also confirmed rapid resolution of the inflammation in these animals. While infected, untreated CF mice suffer from severe pneumonia (Figures S5A–S5D; data not shown). In vitro and in vivo studies with micellar sphingosine or isolated tracheal surfaces from WT or CF mice incubated with Ac, sphingosine, or controls revealed that surface sphingosine kills planctonic and mucoid *P. aeruginosa* strains (Figures S6A–S6F).

Ectopic Expression of β1-Integrins Causes Ac Downregulation, Sphingosine Downregulation, and Infection Susceptibility in Human Airway CF Cells

β1-integrin was also ectopically expressed on the luminal surface of bronchi, nasal polyps, and in isolated nasal epithelial cells from CF patients, while β1-integrin was absent from the surface of healthy controls (Figures 6A–6C). Incubation with Ac-degrading ceramide normalized expression of β1-integrin (Figure 6C). Ac and sphingosine were downregulated in CF bronchial and

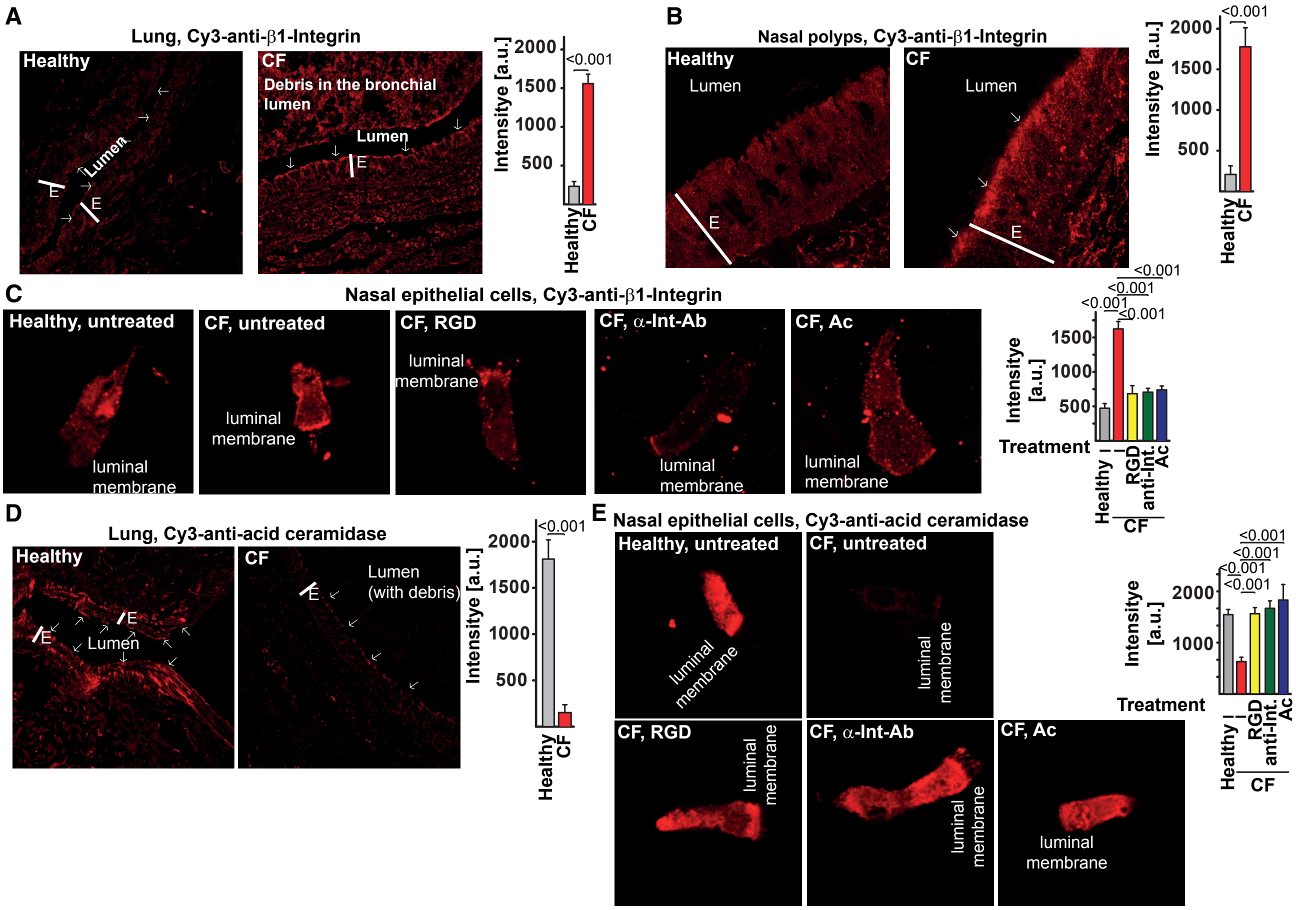

Figure 6. Ectopic Expression of β1-Integrins Causes Acid Ceramidase Downregulation in Human Airway CF Cells

(A–C) Ectopic surface expression of β1-integrin is found on the luminal pole of human CF bronchial epithelial cells (A), epithelial cells of nasal polyps (B), and freshly isolated nasal epithelial cells (C) but is absent from the luminal side of epithelial cells from healthy persons. Incubation with RGD peptides, anti-β1-integrin (Int) antibodies (HUTS-4), or acid ceramidase (Ac) normalized expression of β1-integrin in CF nasal epithelial cells (C). Sections from human CF or donor lungs or nasal polyps or freshly isolated, intact nasal epithelial cells were stained with Cy3-coupled anti-β1-integrin antibodies (Abcam) and analyzed by confocal microscopy. Shown are representative results from each of five CF or four healthy individuals (A and C) or each of four CF or healthy persons (B).

Ac expression is downregulated in CF bronchial and nasal epithelial cells compared to healthy controls, (D) and (E). Normalization of ectopic β1-integrin expression resulted in re-expression of Ac (E). Fluorescence intensities in the luminal membrane were quantified using ImageJ and are given in arbitrary units (a.u.). Mean ± SD; n = 4 or 5; p values as given; t test for comparison of two values and ANOVA and post hoc Student's t tests for all pairwise comparisons, applying Bonferroni correction for multiple comparisons. Overall p value for ANOVA is < 0.001 in all panels. The luminal membrane is indicated by arrows; E = epithelial cell layer. Please see also Figure S7.

nasal epithelial cells compared to healthy controls (Figures 6D, 6E, and 7A–7C), while ceramide was severely upregulated (Figures S7A–S7C). Normalization of ectopic expression of β1-integrin in CF nasal epithelial cells by forced internalization of β1-integrin upon incubation of isolated nasal epithelial cells with anti-β1-integrin antibodies or RGD-peptides mediated re-expression of Ac and normalized both sphingosine and ceramide levels in CF cells (Figures 6E, 7A, 7C, and S7B). We used the HUTS-4 anti-β1-integrin antibody, which binds to the active form of β1-integrin, confirming the previous notion that ectopic surface β1-integrin is active. Conversely, application of Ac normalized the ectopic expression of β1-integrin in CF cells (Figure 6C) as well as the sphingosine and ceramide levels (Figures 7A, 7C, and S7B). Inhalation of sphingosine restored surface sphingosine levels in CF airway cells (Figure 7A).

CF nasal epithelial cells had a reduced capability to kill *P. aeruginosa*, which were readily killed by nasal epithelial cells from healthy controls (Figure S7D). Pre-incubation of CF nasal epithelial cells with anti-β1-integrin antibodies (HUTS-4), RGD-peptides, acid ceramidase, or sphingosine restored killing of *P. aeruginosa* by these cells (Figure S7D). Finally, we confirmed the reduction of Ac activity in cultured primary bronchial epithelial cells from people with CF compared to cells from non-CF individuals (Figure S7E).

DISCUSSION

Our studies have identified a mechanism that explains the high susceptibility of CF patients and mice to acute infections with *P. aeruginosa*. We assume the following scenario: ceramide

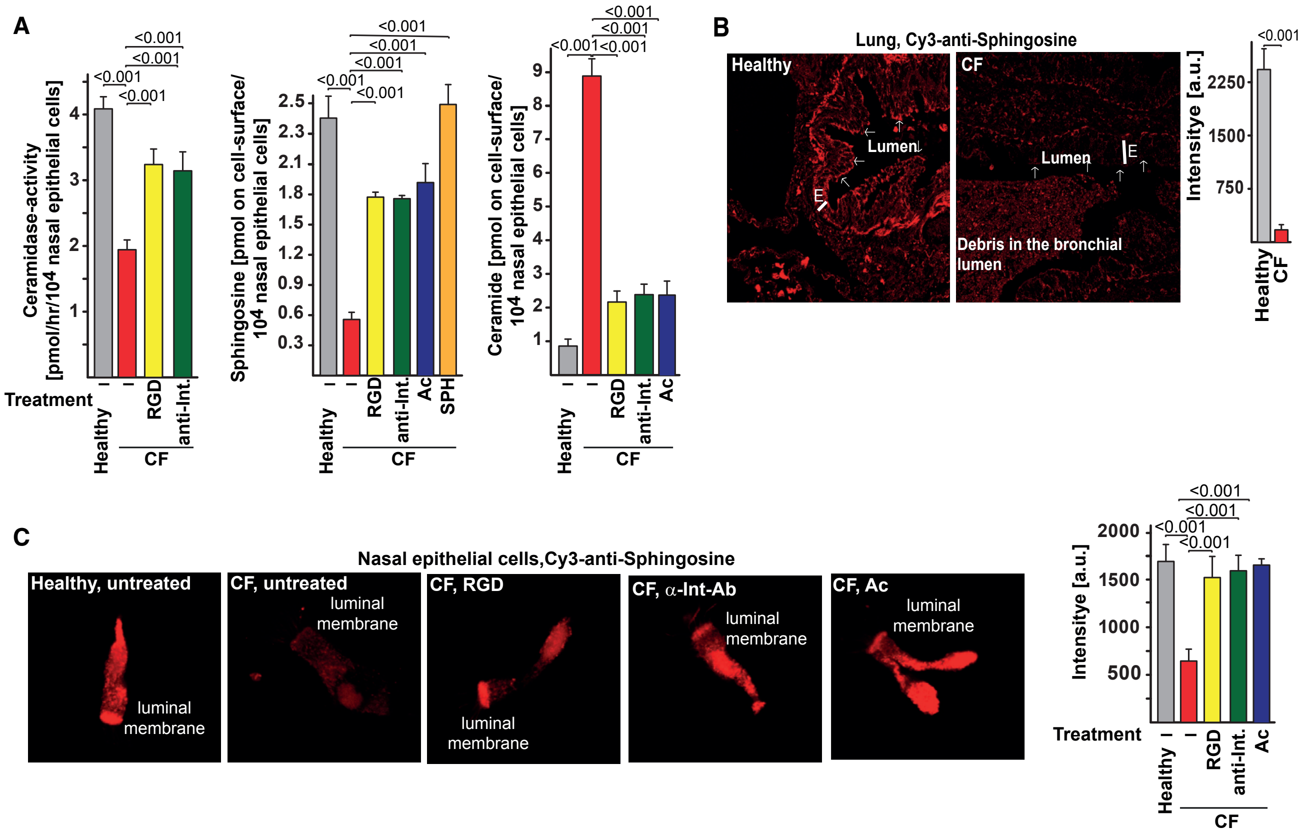

Figure 7. Ectopic Expression of β1-Integrins Reduces Acid Ceramidase Activity and Sphingosine and Increases Ceramide Levels in Human Airway CF Cells

(A–C) Ac activity (A) and sphingosine (A–C) are downregulated in CF bronchial (B) and nasal epithelial cells (A and C) compared to healthy controls, while ceramide (A) is severely upregulated. Normalization of ectopic β1-integrin expression by treatment with RGD peptides or anti-β1-integrin (antibodies (HUTS-4) resulted in normalization of Ac activity (A), re-expression of sphingosine (A–C), and reduction of ceramide (A) levels in CF cells. Incubation of freshly isolated epithelial cells with Ac normalized sphingosine (A and C) and ceramide (A) levels. Treatment with sphingosine restored surface sphingosine (SPH) levels (A). Sections (B) or cells (C) were stained as indicated with Cy3-labeled anti-sphingosine antibodies. Pre-incubations were performed with RGD peptides, HUTS-4 anti-β1-integrin antibodies (α-Int-Ab), Ac, or sphingosine as indicated. (A) Mean ± SD; n = 4 or 5; p-values are indicated; t-test for comparison of two values; ANOVA and post-hoc Student's t-tests for all pairwise comparisons, applying Bonferroni correction for multiple comparisons. Overall p-value for ANOVA is <0.001 in all panels. (B) The luminal membrane is indicated by arrows; E = epithelial cell layer. Please see also Figure S7.

accumulation in CF cells results in trapping and ectopic surface expression of β1-integrins and thereby in downregulation of Ac protein expression via still-unknown mechanisms. The downregulation of Ac expression results in a feed-forward cycle of additional ceramide formation and β1-integrin clustering but also in a marked reduction of sphingosine levels. Increased ceramide levels also trigger the activation of CD95 and thereby mediate increased death of epithelial cells, as previously shown (Teichgräber et al., 2008; Becker et al., 2012). Finally, the combination of low sphingosine concentrations and increased release of dead cells or DNA from the epithelial cell layer into the lumen causes the high susceptibility to infection. In this scenario, sphingosine is the first line of defense, whereas DNA greatly facilitates infection with bacteria, if the bacteria reach the lung, because of the low sphingosine levels in the upper airways. Thus, normalization of the levels of β1-integrin, ceramide, or sphingosine prevents infection.

Sphingosine is also downregulated in the airways of mice after severe burn injury and prevents pulmonary *P. aeruginosa* infections of these mice (Rice et al., 2016). Alterations in sphingolipid metabolism have also been implicated in chronic obstructive pulmonary disease (COPD) (Petrache et al., 2005), a disease that is very often associated with chronic infections with *P. aeruginosa* (Lieberman and Lieberman, 2003). A role of surface β1-integrin expression in these diseases remains to be determined.

The important role of the upper airways in bacterial defense is highlighted by the clinical finding that successful management of the sinuses prior to and after lung transplantation of CF patients (i.e., with lungs with normal expression of Cftr) greatly reduces the incidence of acute pneumonia compared to CF patients without sinus management (Holzmann et al., 2004; Vital et al., 2013).

A recent study found that CF mice lack the ATP12A channel; this deficiency results in a higher resistance to chronic bacterial infections than is found in pigs or in patients with CF that express ATP12A (Shah et al., 2016). Thus, although the lungs of CF mice contain approximately 100 CFU 3 days after

exposure to bacteria, they contain 10,000 CFU at the same time point if ATP12A is expressed in the lung by adenoviral infection (Shah et al., 2016). Our studies with repeated application of mucoid *P. aeruginosa* strains show higher bacterial numbers with 6,000 and 8,000 CFU in the lung of CF mice, and the mucoid strains might be less affected by the pH regulation in the airways. It will be very interesting to study the relation between ATP12A and β1-integrin-acid-ceramidase and sphingosine.

β1-integrin exists in three conformations: the inactive bent state, the extended closed state, and the extended open state (Su et al., 2016). The 9EG7 antibody binds to the integrin-epidermal growth factor domain 2 (I-EGF2), which is exposed in the extended closed or open state, within the β1-integrin molecule, while ligands such as RGD bind to the head of the integrin molecule (Lenter et al., 1993; Su et al., 2016). Thus, ceramide or ceramide-enriched membrane domains trap inactive β1-integrin and seem to induce a conformational change to the active extended closed domain. This is insufficient to induce internalization, but allows binding of the 9EG7 antibody. Subsequent binding of RGD peptides or 9EG7 antibodies to β1-integrin induces and stabilizes the extended open conformation of the molecule, which is fully active and internalized. A similar stabilization of the active conformation might be achieved by binding of the anti-human-β1-integrin antibody HUTS4, thereby triggering internalisation.

In contrast to the studies by Teichgräber et al. (2008); Zhang et al. (2010); Becker et al. (2010); Ulrich et al. (2010); Brodlie et al. (2010a); Bodas et al. (2011); Caretti et al. (2014); Itokazu et al. (2014), and Quinn et al. (2016); Guilbault et al. (2009) described a a decrease of ceramide in CF and an increase of dihydro-ceramide. This might be explained by technical differences, since Guilbault et al. (2009) used an ELISA method employing the 15B4 anti-ceramide antibody to detect ceramide. This antibody also detects dihydro-ceramide. All other studies employed mass spectrometry, kinase assays, or staining with an antibody against ceramide (from Glycobiotech), which does not detect dihydroceramide or an unbiased metabolomic approach. The mouse models between the studies also differ, since Guilbault et al. (2009) used mice completely deficient for *Cftr*, which had to be fed with a Peptamen (Nestle) diet to allow survival. However this diet is inappropriate for use in rodents, since mice consume as much as 75% of their body weight per day of Peptamen, resulting in liver steatosis and dramatic changes of the lipid metabolism (Borowitz et al., 2005) and even a Niemann-Pick type-C phenotype, with a concomitant change of acid sphingomyelinase activity (Bhuvaneswaran et al., 1985). It might be possible that the accumulation of ceramide is observed in mice that are not severely sick, because expression of Cftr is specifically restored in the intestine, while a very severe phenotype with complete deficiency may alter the accumulation of ceramide and switch to an accumulation of dihydroceramide. Such an explanation of a further shift of the ceramide metabolism in CF mice is also suggested by recent studies from Duchesneau et al. (2017), who demonstrated a decrease of ceramide in 8-month-old *Cftr*-deficient mice. Thus, underlying exposure to bacteria, the diet, and the severity of the disease may have an influence on the distribution of ceramide and dihydroceramide in CF lungs.

Our findings also suggest several potential approaches to reducing the incidence and severity of *P. aeruginosa* infections, which are the leading cause of death for patients with CF, or even preventing these infections. Inhalation treatment with anti-β1-integrin antibodies or RGD may be an effective therapy for the prevention of pulmonary *P. aeruginosa* infections. Furthermore, we found that normalizing ceramide levels with pharmacological inhibitors of Asm activity corrects aberrant expression of luminal β1-integrin and thereby prevents infection. Clinical studies with systemic application of amitriptyline have already been initiated and have yielded promising results (Riethmüller et al., 2009; Nährlich et al., 2013; Adams et al., 2016). Inhalation of sphingosine prevents and cures *P. aeruginosa* infections in CF mice but does not seem to influence inflammation in CF lungs (Pewzner-Jung et al., 2014). Finally, inhalation of recombinant Ac by Cf patients might simultaneously correct both the ceramide accumulation and the sphingosine deficiency, thereby correcting the ectopic β1-integrin expression. This is even more attractive given our data showing that CF patients have a severe deficiency of the Ac protein in their airways. These methods suggest realistic treatment options for patients with CF.

STAR★METHODS

Detailed methods are provided in the online version of this paper and include the following:

- KEY RESOURCES TABLE
- CONTACT FOR REAGENT AND RESOURCE SHARING
- EXPERIMENTAL MODEL AND SUBJECT DETAILS
  - Human Materials
  - Mice
- METHOD DETAILS
  - Antibodies and Reagents
  - Bacteria
  - Inhalation Experiments
  - In Vivo Infection Experiments
  - Immunohistochemistry of Mouse Lungs
  - Immunostainings of Human Samples
  - Human Nasal Epithelial Cells
  - Human Bronchial Epithelial Cells
  - Confocal Microscopy
  - β1-Integrin Immunoprecipitations
  - Quantitative Surface Assays
  - Surface Activity of Acid Ceramidase
  - Acid Ceramidase Activity
  - Acid Ceramidase Western Blots
  - Internalization of FITC-Coupled RGD
  - Flow Cytometry Studies
  - Acid Sphingomyelinase Activity
  - Degradation and Uptake Kinetics
  - In Vitro Infection of Trachea Surfaces
  - Ceramide-β1-Integrin Association
  - Inhalation of Anti-sphingosine Antibodies
  - Gr1, *P. aeruginosa*, and Hemalaun Stainings
  - In Vitro Incubation with Sphingosine
  - Sphingosine in Tracheal Lavage
- QUANTIFICATION AND STATISTICAL ANALYSIS
- DATA AND SOFTWARE AVAILABILITY

SUPPLEMENTAL INFORMATION

Supplemental Information includes seven figures and four tables and can be found with this article online at http://dx.doi.org/10.1016/j.chom.2017.05.001.

AUTHOR CONTRIBUTIONS

E.G. initiated the studies, designed the experiments, supervised the research, and performed the infection studies, immunoprecipitation experiments, and enzyme activity measurements. H.G., B.H., R.Z., K.A.B., and A.P.S. performed the confocal and histological studies. S.L., M.K., and J.R. contributed the human samples. J.S. contributed bacterial strains. E.H.S., C.C.C., C.W., and M.J.E. contributed to the planning of the experiments and the design of the studies. M.B. and A.G. contributed to the planning of the experiments and performed enzyme activity measurements on human cells. All authors discussed the results and commented on the manuscript.

ACKNOWLEDGMENTS

We thank S. Harde for excellent technical support. The study was supported by DFG grant GU 335/35-1, DFG grant GRK 2098, and NIH grant 2 R01 HL075 316-09 (to E.G.) and by MRC Clinician Scientist Fellowship MR/M008797/1 (to M.B.).

Received: September 8, 2016
Revised: March 10, 2017
Accepted: May 2, 2017
Published: May 25, 2017

REFERENCES

Adams, C., Icheva, V., Deppisch, C., Lauer, J., Herrmann, G., Graepler-Mainka, U., Heyder, S., Gulbins, E., and Riethmueller, J. (2016). Long-term pulmonal therapy of cystic fibrosis-patients with amitriptyline. Cell. Physiol. Biochem. *39*, 565–572.

Baconnais, S., Tirouvanziam, R., Zahm, J.M., de Bentzmann, S., Péault, B., Balossier, G., and Puchelle, E. (1999). Ion composition and rheology of airway liquid from cystic fibrosis fetal tracheal xenografts. Am. J. Respir. Cell Mol. Biol. *20*, 605–611.

Becker, K.A., Riethmüller, J., Lüth, A., Döring, G., Kleuser, B., and Gulbins, E. (2010). Acid sphingomyelinase inhibitors normalize pulmonary ceramide and inflammation in cystic fibrosis. Am. J. Respir. Cell Mol. Biol. *42*, 716–724.

Becker, K.A., Henry, B., Ziobro, R., Tümmler, B., Gulbins, E., and Grassmé, H. (2012). Role of CD95 in pulmonary inflammation and infection in cystic fibrosis. J. Mol. Med. *90*, 1011–1023.

Bhuvaneswaran, C., Venkatesan, S., and Mitropoulos, K.A. (1985). Lysosomal accumulation of cholesterol and sphingomyelin: evidence for inhibition of acid sphingomyelinase. Eur. J. Cell Biol. *37*, 98–106.

Bodas, M., Min, T., Mazur, S., and Vij, N. (2011). Critical modifier role of membrane-cystic fibrosis transmembrane conductance regulator-dependent ceramide signaling in lung injury and emphysema. J. Immunol. *186*, 602–613.

Borowitz, D., Durie, P.R., Clarke, L.L., Werlin, S.L., Taylor, C.J., Semler, J., De Lisle, R.C., Lewindon, P., Lichtman, S.M., Sinaasappel, M., et al. (2005). Gastrointestinal outcomes and confounders in cystic fibrosis. J. Pediatr. Gastroenterol. Nutr. *41*, 273–285.

Brodlie, M., McKean, M.C., Johnson, G.E., Gray, J., Fisher, A.J., Corris, P.A., Lordan, J.L., and Ward, C. (2010a). Ceramide is increased in the lower airway epithelium of people with advanced cystic fibrosis lung disease. Am. J. Respir. Crit. Care Med. *182*, 369–375.

Brodlie, M., McKean, M.C., Johnson, G.E., Perry, J.D., Nicholson, A., Verdon, B., Gray, M.A., Dark, J.H., Pearson, J.P., Fisher, A.J., et al. (2010b). Primary bronchial epithelial cell culture from explanted cystic fibrosis lungs. Exp. Lung Res. *36*, 101–110.

Caretti, A., Bragonzi, A., Facchini, M., De Fino, I., Riva, C., Gasco, P., Musicanti, C., Casas, J., Fabriàs, G., Ghidoni, R., and Signorelli, P. (2014). Anti-inflammatory action of lipid nanocarrier-delivered myriocin: therapeutic potential in cystic fibrosis. Biochim. Biophys. Acta *1840*, 586–594.

Carpinteiro, A., Becker, K.A., Japtok, L., Hessler, G., Keitsch, S., Požgajovà, M., Schmid, K.W., Adams, C., Müller, S., Kleuser, B., et al. (2015). Regulation of hematogenous tumor metastasis by acid sphingomyelinase. EMBO Mol. Med. *7*, 714–734.

Charizopoulou, N., Jansen, S., Dorsch, M., Stanke, F., Dorin, J.R., Hedrich, H.J., and Tümmler, B. (2004). Instability of the insertional mutation in CftrTgH(neoim)Hgu cystic fibrosis mouse model. BMC Genet. *5*, 6.

Charizopoulou, N., Wilke, M., Dorsch, M., Bot, A., Jorna, H., Jansen, S., Stanke, F., Hedrich, H.J., de Jonge, H.R., and Tümmler, B. (2006). Spontaneous rescue from cystic fibrosis in a mouse model. BMC Genet. *7*, 18.

CF foundation (2010). Patient registry annual report. http://www.cff.org/livingwithcf/carecenternetwork/patientregistry/.

D'Souza, S.E., Ginsberg, M.H., Burke, T.A., Lam, S.C., and Plow, E.F. (1988). Localization of an Arg-Gly-Asp recognition site within an integrin adhesion receptor. Science *242*, 91–93.

Duchesneau, P., Besla, R., Derouet, M.F., Guo, L., Karoubi, G., Silberberg, A., Wong, A.P., and Waddell, T.K. (2017). Partial restoration of CFTR function in cftr-null mice following targeted cell replacement therapy. Mol. Ther. *25*, 654–665.

Elborn, J.S. (2016). Cystic fibrosis. Lancet *388*, 2519–2531.

Forbes, A.R., and Horrigan, R.W. (1977). Mucociliary flow in the trachea during anesthesia with enflurane, ether, nitrous oxide, and morphine. Anesthesiology *46*, 319–321.

Fulcher, M.L., Gabriel, S., Burns, K.A., Yankaskas, J.R., and Randell, S.H. (2005). Well-differentiated human airway epithelial cell cultures. Methods Mol. Med. *107*, 183–206.

Grassmé, H., Kirschnek, S., Riethmueller, J., Riehle, A., von Kürthy, G., Lang, F., Weller, M., and Gulbins, E. (2000). CD95/CD95 ligand interactions on epithelial cells in host defense to *Pseudomonas aeruginosa*. Science *290*, 527–530.

Grassmé, H., Jekle, A., Riehle, A., Schwarz, H., Berger, J., Sandhoff, K., Kolesnick, R., and Gulbins, E. (2001). CD95 signaling via ceramide-rich membrane rafts. J. Biol. Chem. *276*, 20589–20596.

Grassmé, H., Jendrossek, V., Bock, J., Riehle, A., and Gulbins, E. (2002). Ceramide-rich membrane rafts mediate CD40 clustering. J. Immunol. *168*, 298–307.

Grassmé, H., Jendrossek, V., Riehle, A., von Kürthy, G., Berger, J., Schwarz, H., Weller, M., Kolesnick, R., and Gulbins, E. (2003). Host defense against *Pseudomonas aeruginosa* requires ceramide-rich membrane rafts. Nat. Med. *9*, 322–330.

Guilbault, C., Wojewodka, G., Saeed, Z., Hajduch, M., Matouk, E., De Sanctis, J.B., and Radzioch, D. (2009). Cystic fibrosis fatty acid imbalance is linked to ceramide deficiency and corrected by fenretinide. Am. J. Respir. Cell Mol. Biol. *41*, 100–106.

Gulbins, E., Palmada, M., Reichel, M., Lüth, A., Böhmer, C., Amato, D., Müller, C.P., Tischbirek, C.H., Groemer, T.W., Tabatabai, G., et al. (2013). Acid sphingomyelinase-ceramide system mediates effects of antidepressant drugs. Nat Med. *19*, 934–938.

Gut, A., Balda, M.S., and Matter, K. (1998). The cytoplasmic domains of a beta1 integrin mediate polarization in Madin-Darby canine kidney cells by selective basolateral stabilization. J. Biol. Chem. *273*, 29381–29388.

Holzmann, D., Speich, R., Kaufmann, T., Laube, I., Russi, E.W., Simmen, D., Weder, W., and Boehler, A. (2004). Effects of sinus surgery in patients with cystic fibrosis after lung transplantation: a 10-year experience. Transplantation *77*, 134–136.

Horinouchi, K., Erlich, S., Perl, D.P., Ferlinz, K., Bisgaier, C.L., Sandhoff, K., Desnick, R.J., Stewart, C.L., and Schuchman, E.H. (1995). Acid sphingomyelinase deficient mice: a model of types A and B Niemann-Pick disease. Nat. Genet. *10*, 288–293.

Hurwitz, R., Ferlinz, K., and Sandhoff, K. (1994). The tricyclic antidepressant desipramine causes proteolytic degradation of lysosomal sphingomyelinase in human fibroblasts. Biol. Chem. Hoppe Seyler *375*, 447–450.

Itokazu, Y., Pagano, R.E., Schroeder, A.S., O'Grady, S.M., Limper, A.H., and Marks, D.L. (2014). Reduced GM1 ganglioside in CFTR-deficient human airway cells results in decreased β1-integrin signaling and delayed wound repair. Am. J. Physiol. Cell Physiol. *306*, C819–C830.

Kornhuber, J., Tripal, P., Reichel, M., Mühle, C., Rhein, C., Muehlbacher, M., Groemer, T.W., and Gulbins, E. (2010). Functional Inhibitors of Acid Sphingomyelinase (FIASMAs): a novel pharmacological group of drugs with broad clinical applications. Cell. Physiol. Biochem. *26*, 9–20.

Lenter, M., Uhlig, H., Hamann, A., Jenö, P., Imhof, B., and Vestweber, D. (1993). A monoclonal antibody against an activation epitope on mouse integrin chain beta 1 blocks adhesion of lymphocytes to the endothelial integrin alpha 6 beta 1. Proc. Natl. Acad. Sci. USA *90*, 9051–9055.

Lieberman, D., and Lieberman, D. (2003). Pseudomonal infections in patients with COPD: epidemiology and management. Am. J. Respir. Med. *2*, 459–468.

Locke, L.W., Myerburg, M.M., Weiner, D.J., Markovetz, M.R., Parker, R.S., Muthukrishnan, A., Weber, L., Czachowski, M.R., Lacy, R.T., Pilewski, J.M., and Corcoran, T.E. (2016). Pseudomonas infection and mucociliary and absorptive clearance in the cystic fibrosis lung. Eur. Respir. J. *47*, 1392–1401.

Matsui, H., Grubb, B.R., Tarran, R., Randell, S.H., Gatzy, J.T., Davis, C.W., and Boucher, R.C. (1998). Evidence for periciliary liquid layer depletion, not abnormal ion composition, in the pathogenesis of cystic fibrosis airways disease. Cell *95*, 1005–1015.

Matsui, H., Verghese, M.W., Kesimer, M., Schwab, U.E., Randell, S.H., Sheehan, J.K., Grubb, B.R., and Boucher, R.C. (2005). Reduced three-dimensional motility in dehydrated airway mucus prevents neutrophil capture and killing bacteria on airway epithelial surfaces. J. Immunol. *175*, 1090–1099.

Nährlich, L., Mainz, J.G., Adams, C., Engel, C., Herrmann, G., Icheva, V., Lauer, J., Deppisch, C., Wirth, A., Unger, K., et al. (2013). Therapy of CF-patients with amitriptyline and placebo–a randomised, double-blind, placebo-controlled phase IIb multicenter, cohort-study. Cell. Physiol. Biochem. *31*, 505–512.

Nurminen, T.A., Holopainen, J.M., Zhao, H., and Kinnunen, P.K. (2002). Observation of topical catalysis by sphingomyelinase coupled to microspheres. J. Am. Chem. Soc. *124*, 12129–12134.

Petrache, I., Natarajan, V., Zhen, L., Medler, T.R., Richter, A.T., Cho, C., Hubbard, W.C., Berdyshev, E.V., and Tuder, R.M. (2005). Ceramide upregulation causes pulmonary cell apoptosis and emphysema-like disease in mice. Nat. Med. *11*, 491–498.

Pewzner-Jung, Y., Tavakoli Tabazavareh, S., Grassmé, H., Becker, K.A., Japtok, L., Steinmann, J., Joseph, T., Lang, S., Tuemmler, B., Schuchman, E.H., et al. (2014). Sphingoid long chain bases prevent lung infection by *Pseudomonas aeruginosa*. EMBO Mol. Med. *6*, 1205–1214.

Quinn, R.A., Lim, Y.W., Mak, T.D., Whiteson, K., Furlan, M., Conrad, D., Rohwer, F., and Dorrestein, P. (2016). Metabolomics of pulmonary exacerbations reveals the personalized nature of cystic fibrosis disease. PeerJ *4*, e2174.

Rice, T.C., Seitz, A.P., Edwards, M.J., Gulbins, E., and Caldwell, C.C. (2016). Frontline science: sphingosine rescues burn-injured mice from pulmonary Pseudomonas aeruginosa infection. J. Leukoc. Biol *100*, 1233–1237.

Riethmüller, J., Anthonysamy, J., Serra, E., Schwab, M., Döring, G., and Gulbins, E. (2009). Therapeutic efficacy and safety of amitriptyline in patients with cystic fibrosis. Cell. Physiol. Biochem. *24*, 65–72.

Shah, V.S., Meyerholz, D.K., Tang, X.X., Reznikov, L., Abou Alaiwa, M., Ernst, S.E., Karp, P.H., Wohlford-Lenane, C.L., Heilmann, K.P., Leidinger, M.R., et al. (2016). Airway acidification initiates host defense abnormalities in cystic fibrosis mice. Science *351*, 503–507.

Su, Y., Xia, W., Li, J., Walz, T., Humphries, M.J., Vestweber, D., Cabañas, C., Lu, C., and Springer, T.A. (2016). Relating conformation to function in integrin α5β1. Proc. Natl. Acad. Sci. USA *113*, E3872–E3881.

Tarran, R., Grubb, B.R., Gatzy, J.T., Davis, C.W., and Boucher, R.C. (2001). The relative roles of passive surface forces and active ion transport in the modulation of airway surface liquid volume and composition. J. Gen. Physiol. *118*, 223–236.

Tavakoli Tabazavareh, S., Seitz, A., Jernigan, P., Sehl, C., Keitsch, S., Lang, S., Kahl, B.C., Edwards, M., Grassmé, H., Gulbins, E., and Becker, K.A. (2016). Lack of sphingosine causes susceptibility to pulmonary *Staphylococcus aureus* infections in cystic fibrosis. Cell. Physiol. Biochem. *38*, 2094–2102.

Teichgräber, V., Ulrich, M., Endlich, N., Riethmüller, J., Wilker, B., De Oliveira-Munding, C.C., van Heeckeren, A.M., Barr, M.L., von Kürthy, G., Schmid, K.W., et al. (2008). Ceramide accumulation mediates inflammation, cell death and infection susceptibility in cystic fibrosis. Nat. Med. *14*, 382–391.

Tran Van Nhieu, G., and Isberg, R.R. (1993). Bacterial internalization mediated by beta 1 chain integrins is determined by ligand affinity and receptor density. EMBO J. *12*, 1887–1895.

Ulrich, M., Worlitzsch, D., Viglio, S., Siegmann, N., Iadarola, P., Shute, J.K., Geiser, M., Pier, G.B., Friedel, G., Barr, M.L., et al. (2010). Alveolar inflammation in cystic fibrosis. J. Cyst. Fibros. *9*, 217–227.

Vital, D., Hofer, M., Benden, C., Holzmann, D., and Boehler, A. (2013). Impact of sinus surgery on pseudomonal airway colonization, bronchiolitis obliterans syndrome and survival in cystic fibrosis lung transplant recipients. Respiration *86*, 25–31.

Ye, Y., Bloch, S., Xu, B., and Achilefu, S. (2006). Design, synthesis, and evaluation of near infrared fluorescent multimeric RGD peptides for targeting tumors. J. Med. Chem. *49*, 2268–2275.

Zhang, Y., Li, X., Grassmé, H., Döring, G., and Gulbins, E. (2010). Alterations in ceramide concentration and pH determine the release of reactive oxygen species by Cftr-deficient macrophages on infection. J. Immunol. *184*, 5104–5111.

STAR★METHODS

KEY RESOURCES TABLE

REAGENT or RESOURCE	SOURCE	IDENTIFIER
Antibodies		
rabbit anti-human β1-integrin antibodies	Abcam	#Ab52971 RRID: AB_870695
secondary Cy3-coupled anti-rabbit $F(ab)_2$ fragments	Jackson ImmunoResearch	#711-166-152 RRID: AB_2313568
rat anti-mouse β1-integrin antibodies	Merck Millipore	clone MB1.2, #MAB1997 RRID: AB_2128202
Cy3-coupled anti-rat $F(ab)_2$ fragments	Jackson ImmunoResearch	#712-166-153 RRID: AB_2340669
anti-acid ceramidase antibodies	ProSci	#4741 RRID: AB_10909506
anti-β1-integrin antibodies clone 9EG7	BD Biosciences	#550531 RRID: AB_393729
monoclonal mouse anti-ceramide antibodies	Glycobiotech	clone S58-9, #MAB_0011
monoclonal mouse anti-sphingosine antibodies	Alfresa Pharma Corporation	clone NHSPH, #ALF-274042010 RRID: AB_1962849
Cy5-coupled donkey anti-mouse IgM antibodies	Jackson ImmunoResearch	#715-176-020
anti-Art1-antibodies	Santa Cruz Inc.	#sc-20255 RRID: AB_2290018
anti-Ly-6G/Ly-6C	BD	#553122 RRID: AB_394638
anti-*P. aeruginosa* antibodies	Biotrend	#AP086
Bacterial and Virus Strains		
ATCC 27853	ATCC	Boston 41501
762	University of Duisburg-Essen	Grassmé et al., 2000, 2003
1242	University of Duisburg-Essen	this study
1245	University of Duisburg-Essen	this study
Biological Samples		
Freshly isolated human airway epithelial cells	Newcastle University	this study and Brodlie et al., 2010b
Human lung tissue	University of Duisburg-Essen	this study
Human nasal epithelial cells	University of Tuebingen	this study
Human nasal polyps	University of Duisburg-Essen	this study
Chemicals, Peptides, and Recombinant Proteins		
Amitriptyline	Sigma	#8404-25G
Fluoxetine	Ratiopharm	#PZN-3971342
GRGDNP peptide	Enzo Life Sciences	#P-700
GRADSP peptides	Bachem	#H-7630
Paraformaldehyde	Roth	#0335.3
Protein A/G agarose	Santa Cruz Inc.	#sc-2003
Sphingosine kinase 1	R&D	#6068-SK-010
Diacylglycerol (DAG) kinase	Enzo	#BML-SE100
$[^{14}C_{16}]$Ceramide	ARC	#ARC-0831
N-oleoylethanolamine	Sigma	#00383
Carmofur	Cayman Chemicals	#14243
$[^{14}C]$-Sphingomyelin	Perkin Elmer	#NEC 663010UC
Proteinase K	QIAGEN	#03115828001
FITC-Annexin	Roche	#11 828 681 001
Experimental Models: Organisms/Strains		
Congenic B6.129P2(CF/3)-*Cftr*$^{TgH(neoim)Hgu}$	Medizinische Hochschule Hannover and University of Duisburg-Essen	Charizopoulou et. al., 2004, 2006; Teichgräber et al., 2008
Cftr^{tm1Unc}-*Tg*$^{(FABPCFTR)}$	Jackson Laboratory	Stock No: #002364
B6-*Cftr*^{tm1Unc}-*Tg*$^{(FABPCFTR)}$*Smpd1*$^{-/-}$	University of Duisburg-Essen	Teichgräber et al., 2008

(*Continued on next page*)

Continued

REAGENT or RESOURCE	SOURCE	IDENTIFIER
B6-Smpd1$^{fl\text{-}tr/fl\text{-}tr}$	University of Duisburg-Essen	Gulbins et al., 2013
B6-Asah1$^{fl\text{-}del/fl\text{-}del}$	University of Duisburg-Essen	Gulbins et al., 2013
Software and Algorithms		
ImageJ	NIH	https://imagej.nih.gov/ij/
G Power	University of Duesseldorf	http://www.gpower.hhu.de

CONTACT FOR REAGENT AND RESOURCE SHARING

Further information and requests for resources should be directed to and will be fulfilled by the lead contact (erich.gulbins@uni-due.de).

EXPERIMENTAL MODEL AND SUBJECT DETAILS

Human Materials

Polyps from healthy and CF persons were surgically removed, fixed in 4% phosphate-buffered saline (PBS)-buffered paraformaldehyde (PFA), embedded in paraffin, dewaxed, sectioned, and stained (see section below on Immunohistochemistry of Human Samples). Biopsies from explanted CF or remaining donor lungs were also fixed in 4% PFA. Nasal epithelial cells were obtained from CF-patients and healthy volunteers by nasal brushing and freshly used as described below. Primary bronchial epithelial cells were obtained from explanted CF lungs and were isolated as previously described (Brodlie et al., 2010b) or from bronchial brushings of non-CF donors. We used samples from female and male patients. The identity of the patients and donors, respectively, was blinded to the investigators. The studies were approved by the local ethics committees Essen, Tuebingen and Newcastle and North Tyneside, all patients gave informed consent. The permission numbers were 17-7326-BO, 08-3710, 214-003581-25, 01/179 and 11/NE/0291.

Mice

B6.129P2(CF/3)-*Cftr*$^{\mathrm{TgH(neoim)Hgu}}$ (CF^{MHH}) congenic mice were produced by inbreeding the original *Cftr*TgH$^{\mathrm{(neoim)}}$Hgu mutant mouse, which was generated by insertional mutagenesis in exon 10 of the Cftr gene (Charizopoulou et al., 2004, 2006). This congenic *Cftr^{MHH}* strain was then backcrossed for more than 10 generations into the B6 background. Because these mice still express low levels of Cftr, they can be fed a standard mouse diet. They exhibit normal development but also display pulmonary pathology typical of CF (Teichgräber et al., 2008; Zhang et al., 2010; Becker et al., 2010, 2012; Ulrich et al., 2010). Syngenic B6 mice were used as controls. Further, we used *Cftr^{tm1Unc}-Tg$^{(FABPCFTR)}$* mice (*Cftr$^{-/-}$*; Jackson Laboratory, Bar Harbor, ME, USA) backcrossed for more than 10 generations onto the C57BL/6 background. These mice are deficient in *Cftr* in all organs except the intestine, where they express human CFTR under the control of a fatty acid binding protein (FABP) promoter. The transgene prevents intestinal obstruction and allows the mice to eat a normal diet. B6 mice were again used as controls. No important differences were observed in the present experiments, which used both the *Cftr$^{-/-}$* and the *Cftr^{MHH}* strains.

To create mice deficient in *Cftr* and heterozygous for *Asm*, we crossed *Cftr$^{-/-}$* mice with C57BL/6 mice lacking Asm (gene symbol *Smpd1*) (Horinouchi et al., 1995) to obtain B6-*Cftr^{tm1Unc}-Tg$^{(FABPCFTR)}$Smpd1$^{-/-}$* mice (Teichgräber et al., 2008). Syngenic B6 and *Cftr$^{-/-}$* mice were used as controls. For downregulation of ceramide production via Asm inhibition, mice were treated with two functional inhibitors of Asm: amitriptyline hydrochloride (Sigma, #8404-25G) and fluoxetine (Ratiopharm GmbH, #PZN-3971342) (Teichgräber et al., 2008). Amitriptyline (10 mg/kg; dissolved in 0.9% NaCl) was administered via intraperitoneal injection every 12 hr for a total of five doses, and mice were sacrificed within 6 hr after the final injection. Fluoxetine was dissolved in drinking water (120 mg/L), which was changed every 2 days for more than 21 days.

To create overexpressing Asm-transgenic mice (Gulbins et al., 2013), we expressed murine *Smpd1* cDNA under control of the ubiquitous CAG promoter (cytomegalovirus [CMV] immediate-early enhancer/chicken β-actin promoter fusion). A loxP-flanked STOP cassette was included between the promoter and the transgene. The conditional transgene was introduced into the deleted Hprt gene locus of E14 embryonic stem cells. The transgenic mice were backcrossed to C57BL/6 mice for at least 10 generations. The strain name is *B6-Smpd1$^{fl\text{-}tr/fl\text{-}tr}$*. In the present study we expressed the transgene constitutively by crossing the mice with E2A-Cre mice expressing Cre recombinase under the control of an E2A promoter (kindly provided by Dr. R. Waldschütz). To obtain heterozygous acid ceramidase 1 (*Asah1*)-deficient mice (Gulbins et al., 2013), we constructed a targeting vector containing the following: homology regions of genomic *Asah1* sequences in the C57BL/6J genetic background, a long homology region of 5.8 kb and a short homology region of 1.7 kb, two loxP sites flanking *Asah1* exon 1, a neomycin gene flanked by flippase recognition target (FRT) sites for positive selection (the FRT-flanked selection cassette was removed in vivo with recombinase), and a diphtheria toxin A negative-selection marker for reducing the isolation of non-homologous recombined embryonic stem (ES) cell clones and enhancing the isolation of ES cell clones harboring the distal loxP site. The targeting vector was transfected into C57BL/6 embryonic stem cells by homologous recombination to obtain *B6-Asah1$^{fl\text{-}del/fl\text{-}del}$*. Mice heterozygous for Ac gene knockout allele were generated by crossing with E2A-Cre mice.

We used female and male mice with an age of at least 16 weeks and a weight between 25 and 35 g. Mice were divided into cages of equal size (usually 3-4 mice) by animal unit technical staff with no involvement in study design. Cages were randomly assigned to an experimental group. The investigators were blinded to the group allocation during the experiment and/or when assessing the outcome.

Mice were housed and bred within isolated cages in the mouse facility of the University Hospital, University of Duisburg-Essen, Germany and of the University of Cincinnati. They were repeatedly tested for a panel of common murine pathogens according to the 2002 recommendations of the Federation of European Laboratory Animal Science Associations. The mice were free of all pathogens. Procedures performed on the animals were approved by the Bezirksregierung Duesseldorf, Duesseldorf, Germany and the IACUC committeee Cincinnati. Permission numbers are: G1380-13, G1376-13, G1121-10, AZ 84.02.04.2015.A064 and 10-05-10-01

METHOD DETAILS

Antibodies and Reagents

Human β1-integrin was stained with rabbit anti-human β1-integrin antibodies (#Ab52971; Abcam) and labeled with secondary Cy3-coupled anti-rabbit $F(ab)_2$ fragments (#711-166-152; Jackson ImmunoResearch). Paraffin-embedded mouse tissues were stained with rat anti-mouse β1-integrin antibodies (clone MB1.2, #MAB1997; Merck Millipore) followed by incubation with Cy3-coupled anti-rat $F(ab)_2$ fragments (#712-166-153; Jackson ImmunoResearch). Acid ceramidase was stained with Cy3-coupled anti-acid ceramidase antibodies (#4741, ProSci). In vivo binding of β1-integrin was achieved by application, injection or inhalation of the anti-β1-integrin antibody clone 9EG7 (1 μg/ml; #550531; BD Biosciences). Isotype-matched antibodies were used as controls. These were mouse IgM for the antibodies against sphingosine and ceramide, rat IgG2 for the staining with anti-integrin antibodies and purified rabbit IgG for the stainings with anti-acid ceramidase (mouse IgM and purified rabbit IgG from Dako, rat IgG2 from R&D). All ceramide or sphingosine stainings were performed with the monoclonal mouse anti-ceramide antibody (clone S58-9, #MAB_0011, Glycobiotech), or monoclonal mouse anti-sphingosine antibody (clone NHSPH, #ALF-274042010, Alfresa Pharma Corporation), which were visualized with Cy3 donkey anti-mouse IgM $F(ab)_2$ fragments (#715-166-020; Jackson ImmunoResearch) or Cy5-coupled donkey anti-mouse IgM antibody (#715-176-020; Jackson ImmunoResearch). GRGDNP peptide was obtained from Enzo Life Sciences (#P-700). GRADSP peptides (Bachem, #H-7630) served as controls.

Bacteria

We used the previously described laboratory strain American Type Culture Collection (ATCC) 27853 *P. aeruginosa*, a clinical *P. aeruginosa* isolate 762 (Grassmé et al., 2000, 2003) and two mucoid isolates from individuals with CF, *P. aeruginosa* strains 1242 and 1245. Bacteria were plated from frozen stocks on fresh tryptic soy agar plates (TSA; Becton Dickinson) and grown at 37°C for 14 to 16 hr. The strains 762 and ATCC 27853 were resuspended in 40 mL tryptic soy broth (Becton Dickinson), warmed to 37°C, to an optical density of 0.225 at 550 nm. The bacterial suspension was then incubated at 37°C for 1 hr with shaking at 125 rpm to obtain bacteria in the early logarithmic growth phase. Bacteria were then washed twice and resuspended in warmed RPMI 1640 medium (Invitrogen) supplemented with 10 mM HEPES. The final concentration of bacteria was quantified by photospectrometry. The two mucoid *P. aeruginosa* strains 1242 and 1245 were directly taken from the plate, resuspended and immediately used for infection to ensure the mucoid form.

Inhalation Experiments

Mice inhaled 0.8 mL of 325 μM RGD peptides (GRGDNP) or 20 μg/ml anti-β1-integrin antibody 9EG7 diluted in 0.9% saline via a modified PARI BOY nebulizer device (PARI GmbH, Starnberg, Germany), as previously described (Teichgräber et al., 2008; Pewzner-Jung et al., 2014). Control animals received 0.8 mL nebulized 0.9% saline. Although the nebulizer was loaded with 0.8 mL NaCl ± peptides/antibodies, the mice only inhaled approximately 0.08 mL (i.e., also 10% of the drugs) over the 10 min inhalation period, while the majority of the fluid got lost in the device or around the nose of the mouse.

Mice were sacrificed 15 min after inhalation to detect surface β1-integrin in situ. For determination of the effects of GRGDNP peptides or anti-β1-integrin antibody 9EG7 on the internalization of ceramide, sphingosine, and β1-integrin, mice were sacrificed 60 min after inhalation.

In Vivo Infection Experiments

Mice were infected with 762 or ATCC 27853 *P. aeruginosa* strains 60 min after inhalation. Mice were lightly anesthetized with diethyl ether, an agent that does not affect ciliary function (Forbes and Horrigan, 1977; Teichgräber et al., 2008). *P. aeruginosa* were prepared as described above and resuspended in RPMI 1640 plus 10 mM HEPES to a final concentration of 5×10^7 or 5×10^6 CFU in 20 μL medium. The mice were then inoculated with 5×10^7 or 5×10^6 CFU *P. aeruginosa* 762 or ATCC 27853 via a plastic-coated 30-gauge needle, which was inserted 2 mm into the nose. Bacteria in mouse lungs were counted 4 hr after infection. Mice were sacrificed; the lungs were removed, homogenized, and lysed in 5 mg/mL saponin to release intracellular bacteria. Samples were then centrifuged at 3200 rpm to pellet all bacteria, washed in sterile PBS, diluted, and plated in duplicate onto TSA plates for 12 hr. Bacterial numbers were counted; the counts represented the number of bacteria in whole-lung samples. This method of infection evaluates mucociliary clearance more accurately than do other pulmonary infection models, such as intratracheal infection.

Infection with the mucoid *P. aeruginosa* strains 1242 and 1245 was performed with 1×10^7 CFU in 20 μL medium and repeated 5 times on consecutive days. Mice were then inhaled 1 hr after the last infection with acid ceramidase, sphingosine or RGD-peptides and the number of the CFU in the lung was determined 5 hr later (6 hr after infection). Alternatively, the mice were treated with acid ceramidase, sphingosine or RGD-peptides 2 days after the last of 5 infections with *P. aeruginosa* strains 1242 or 1245 and the number of the bacteria in the lungs were determined after an additional 48 hr. Finally, we inhaled mice first with acid ceramidase, sphingosine or RGD and infected them 1 hr later with 5×10^7 CFU *P. aeruginosa* strains 1242 or 1245. Bacterial CFU in the lung were determined 6 hr later.

In addition, we determined the number of bacteria in the lower third of the trachea 15, 30, 45, and 60 min after infection with 10^5 CFU; the sum of these counts represented the cumulative number of bacteria reaching the lower third of the trachea within 1 hr after initiation of infection. This number reflects the clearance of the bacteria in the upper airways.

Immunohistochemistry of Mouse Lungs

Stainings were performed as previously reported (Grassmé et al., 2001, 2003; Teichgräber et al., 2008; Pewzner-Jung et al., 2014). For immunohistochemical evaluation of murine lungs, mice were sacrificed by cervical dislocation and were immediately perfused via the right heart with ice-cold normal saline for 2 min followed by cardiac perfusion with 4% PBS-buffered paraformaldehyde (PFA, #0335.3, Roth) for 10 to 15 min. After being cleared of blood and fixed, the lungs were removed and further fixed in 4% PFA for 24 to 36 hr. The tissue was serially dehydrated with an Ethanol to Xylol gradient and then embedded in paraffin. Samples were sectioned at 7 μm, dewaxed, rehydrated, and treated with pepsin (Digest All; #003009, Invitrogen) for 15 min (β1-Integrin) or 30 min (acid ceramidase, ceramide and sphingosine) at 37°C. They were then washed with water and PBS and blocked for 10 min at room temperature with PBS, 0.05% Tween 20 (Sigma), and 5% fetal calf serum (FCS). The samples were stained with a rat anti-mouse β1-integrin (1:100 dilution, clone MB1.2, Merck Millipore), anti-acid ceramidase (1:100 dilution), anti-ceramide (1:100 dilution), anti-sphingosine (1:1000 dilution), anti-mouse β2-integrin antibodies (1:100, clone M1812, 1:100, #557437, BD) or FITC-Annexin (1:200, #11 828 681 001, Roche) in H/S (132 mM NaCl, 20 mM HEPES [pH 7.4], 5 mM KCl, 1 mM $CaCl_2$, 0.7 mM $MgCl_2$, 0.8 mM $MgSO_4$) plus 1% FCS at room temperature for 45 min. Samples were washed three times with PBS plus 0.05% Tween 20 and once with PBS. The tissue was secondarily labeled with Cy3-coupled anti-rat/rabbit/mouse $F(ab)_2$ fragments (Jackson Immunoresearch) in H/S plus 1% FCS for 30 min. Tissue was again washed three times with PBS plus 0.05% Tween 20 and once with PBS; finally, they were embedded in Mowiol. Samples were evaluated by confocal microscopy as described below.

In experiments involving the intraluminal administration of rat anti-mouse β1-integrin antibody 9EG7 to mice, mice were anesthetized, 1 μg of the antibody in 10 μL H/S was slowly injected into the lung via the trachea and allowed to bind for 15 min, the mice were sacrificed by cervical dislocation, the lungs were washed 10-times with PBS via bronchioalveolar lavage, and 1 mL of 2% PBS-buffered PFA (pH 7.4) was instilled into the lung. After 15 min of fixation, the lungs were removed and further fixed in 4% PBS-buffered PFA for 24 to 36 hr, dehydrated, embedded, and sectioned as above. Stainings were carried out by rehydrating the samples and treating them with Proteinase K (dilution 1:1000 in 30 mM Tris HCL; pH 8.0, #03115828001, QIAGEN) for 10 min at 37°C. They were washed with water and PBS and then directly stained with Cy3-coupled anti-rat $F(ab)_2$ fragments for 30 min. Slides were again washed three times in PBS plus 0.05% Tween 20 and once with PBS. Samples were embedded in Mowiol and evaluated with confocal microscopy as described below.

All immunostainings were controlled with isotype control antibodies that showed no or very weak staining. These were mouse IgM for the antibodies against sphingosine and ceramide, rat IgG2 for the staining with anti-integrin antibodies and purified rabbit IgG for the stainings with anti-acid ceramidase (mouse IgM and purified rabbit IgG from Dako, rat IgG2 from R&D). We also included controls with secondary Cy3-coupled antibodies only. Further controls were stainings with FITC-Annexin and stainings with anti-β2-integrin antibodies as above.

Immunostainings of Human Samples

Human nasal polyps or lung tissues were fixed in 4% PFA/PBS for 48 hr. Samples were dehydrated and embedded in paraffin, sectioned at 7 μm, dewaxed, rehydrated, and treated with pepsin solution for 7 min for anti-human β1-integrin antibodies (Abcam), for 40 min for anti-ceramide antibodies and 30 min for anti-sphingosine antibodies. Isolated nasal epithelial cells were first treated as described below, washed and then fixed in 1% PFA/PBS for 10 min and washed in PBS. All samples were blocked with PBS plus 0.05% Tween 20 and 5% FCS at room temperature for 10 min. The samples were washed and stained with either rabbit anti-human β1-integrin antibody (Abcam) (1:75 dilution), anti-ceramide (1:100), anti-acid ceramidase (1:100) or anti-sphingosine antibodies (1:1000) in H/S buffer plus 1% FCS for 45 min at room temperature. Histologies were washed 3-washed in PBS plus 0.05% Tween 20 and again in PBS. Cells were washed 3-times in PBS. The antibodies were secondarily labeled with Cy3-coupled anti-rabbit IgG, or anti-mouse IgM $F(ab)_2$ fragments. They were diluted in H/S plus 1% FCS and incubated in the dark for 30 min. The tissue was again washed as above before being embedded in Mowiol.

Human Nasal Epithelial Cells

Cells were isolated from CF patients and healthy controls (age and sex matched) using a nasal brush. The cells were incubated in RPMI-1640 containing 1% FCS, 1 mM HEPES (pH 7.4), 0.2 mM L-glutamine, 0.1 mM sodium pyruvate, 10 μM nonessential amino acids for 4 hr at 4°C with RGD-peptides, anti-human active β1-integrin antibody (1:100, clone HUTS-4, #MAB2097Z, Millipore), acid ceramidase (25 units), 1 μM micellar sphingosine (#271048, Avanti Polar Lipids) or left untreated. Cells were incubated for an

additional 90 min at 37°C and washed twice in H/S by centrifugation for 10 min at 2500 rpm. Cells were then fixed for 10 min in 1% PFA/PBS for immunostainings. The cells were then washed twice in H/S, blocked by 15 min incubation in H/S + 5% FCS, washed once and stained with anti-sphingosine, anti-ceramide or anti-acid ceramidase antibodies, rabbit anti-human anti-β1-integrin antibody (1:100, #Ab52971; Abcam) or isotype controls. The samples were washed twice. All samples were incubated with the appropriate Cy3-labeled secondary antibody as above. Samples were washed again twice and the cells were embedded in Mowiol for confocal microscopy analysis. An aliquot of all samples treated with RGD-peptides, anti-integrin-antibodies, acid ceramidase or sphingosine or left untreated was washed in H/S and infected with *P. aeruginosa* strains ATCC 27853 or 762 for 60 min at an multiplicity of infection of 1 cell: 0.5 bacteria in H/S. Samples were plated on LB-agar and colonies were counted after overnight growth.

Human Bronchial Epithelial Cells

Primary bronchial epithelial cells were cultured at an air liquid interface (Brodlie et al., 2010b). 1 × 10^5 cells/mL in 0.5 mL of bronchial epithelial growth medium were seeded into the apical chamber of the 12 mm Transwell inserts coated with type IV collagen with 1.5 mL media in the basal chamber. Following cell attachment apical medium was removed and the basal medium switched to air liquid interface specific media, essentially a 50:50 mixture of bronchial epithelial growth medium with DMEM-H (Fulcher et al., 2005). Cells were cultured for 24 days to allow proper differentiation and polarization, which was determined by the presence of ciliated and mucus-secreting cells.

Confocal Microscopy

Samples were examined with a Leica TCS-SP5 confocal microscope equipped with a 100 × lens, and images were analyzed with Leica LCS software version 2.61 (Leica Microsystems, Mannheim, Germany). All comparative samples were measured at identical settings.

β1-Integrin Immunoprecipitations

To demonstrate ectopic expression of β1-integrin on the luminal surface of the trachea, we removed the trachea, opened it, and incubated the intact luminal surface on ice for 20 min with 4 μL of anti-β1-integrin antibodies, clone MB1.2. The samples were washed extensively; lysed in 125 mM NaCl, 25 mM Tris HCl (pH 7.4), 10 mM EDTA, 10 mM sodium pyrophosphate, 3% Nonidet P40, and 10 μg/ml aprotinin and leupeptin for 5 min at 4°C. They were then centrifuged at 14,000 rpm. The immunocomplexes were immobilized with protein A/G agarose (#sc-2003, Santa Cruz Inc.) for 45 min, washed 6 times in lysis buffer, resuspended in 1x SDS-Laemmli Sample buffer, and boiled for 5 min at 95°C. Proteins were separated on 8.5% sodium dodecyl sulfate polyacrylamide gel electrophoresis (SDS-PAGE) gels, blotted with β1-integrin antibody clone MB1.2, and developed with an AP-coupled secondary antibody and a chemoluminescence system.

Quantitative Surface Assays

Sphingosine

Mice were sacrificed, and the trachea was removed, opened, and placed on a 30°C prewarmed plastic plate. The luminal surface of the trachea was incubated with 0.001 units of sphingosine kinase 1 (#6068-SK-010, R&D) in 4 μL of 150 mM sodium acetate (pH 7.4), 1 μM ATP, and 10 μCi [^{32}P]γATP. Controls were incubated with the same buffer without sphingosine kinase or were left untreated. We ensured that the kinase buffer was added only to the luminal surface of the trachea. Nasal epithelial cells were washed, resuspended in 150 mM sodium acetate (pH 7.4), 1 μM ATP, 10 μCi [^{32}P]γATP and 0.001 units of sphingosine kinase and incubated for 30 min at 37°C. The sphingosine kinase reaction was terminated by adding 100 μL H_2O, followed by the addition of 20 μL 1N HCl, 800 μL $CHCl_3$:CH_3OH:1N HCl (100:200:1, v:v:v), and 240 μL each of $CHCl_3$ and 2 M KCl. The lower phase was collected, dried, dissolved in 20 μL $CHCl_3$:CH_3OH (1:1, v/v), and separated on Silica G60 TLC plates with $CHCl_3$:CH_3OH:acetic acid:H_2O (90:90:15:5, v:v:v:v) as a developing solvent. The TLC plates were analyzed with a phosphoimager. Surface sphingosine levels were determined with a standard curve of C18-sphingosine.

Ceramide

The intact epithelial surface of the trachea was incubated with 0.01 units diacylglycerol (DAG) kinase (#BML-SE100, Enzo) in 4 μL of 150 mM sodium acetate (pH 7.4), 1 mM adenosine triphosphate (ATP), and 10 μCi [^{32}P]γATP for 15 min at 30°C. Controls were incubated with the same buffer without kinase or were left untreated. Nasal epithelial cells were washed, resuspended in 150 mM sodium acetate (pH 7.4), 1 μM ATP, 10 μCi [^{32}P]γATP and 0.01 units of diacylglycerol kinase and incubated for 30 min at 37°C. The kinase reaction was terminated by transferring the trachea into $CHCl_3$:CH_3OH:1N HCl (100:100:1, v/v/v), followed by the addition of 170 μL buffered saline solution (135 mM NaCl, 1.5 mM $CaCl_2$, 0.5 mM $MgCl_2$, 5.6 mM glucose, 10 mM HEPES; pH 7.2) and 30 μL of 100 mM EDTA. Lipids were extracted, separated by Silica G60 thin-layer chromatography (TLC), and analyzed with liquid scintillation counting and a standard curve of C16- to C24-ceramides, as previously described (Grassmé et al., 2003; Teichgräber et al., 2008; Pewzner-Jung et al., 2014).

Surface Activity of Acid Ceramidase

We injected 4 μL [$^{14}C_{16}$]ceramide (55 mCi/mmol, #ARC-0831, ARC) into the lumen of the trachea of anesthetized mice. Before injection, [$^{14}C_{16}$]ceramide was dried, resuspended in 0.05% octylglucopyranoside in 150 mM sodium acetate (pH 7.4 or 5.0), and bath sonicated for 10 min. After 20 min, mice were sacrificed, the trachea was carefully removed and extracted in H_2O and

$CHCl_3$:CH_3OH:HCl (100:100:1, v/v/v). The lower phase was dried, and samples were resuspended in $CHCl_3$:CH_3OH (1:1, v/v), separated by TLC with $CHCl_3$:CH_3OH:NH_4OH (90:20:0.5, v/v/v), and analyzed with a Fuji Imager. Injection of 4 μL of the acid ceramidase inhibitors N-oleoylethanolamine (0.5 mM, #00383, Sigma) or carmofur (1 μM, #14243, Cayman Chemicals) onto the trachea was performed 15 min before the injection of [$^{14}C_{16}$]-ceramide and served to demonstrate the contribution of acid ceramidase to the observed ceramide consumption.

Acid Ceramidase Activity

Human nasal epithelial cells

Cells were lysed in 1% Nonidet P40 (NP40) in 150 mM sodium acetate (pH 4.5) for 5 min on ice, diluted to 0.1% NP40 in 150 mM sodium acetate (pH 4.5) and 0.3 μCi/sample [$^{14}C_{16}$]ceramide (ARC0831, 55 mCi/mmol) was added. To this end, the [$^{14}C_{16}$]ceramide was dried for 10 min, resuspended in 0.1% OGP in 150 mM sodium acetate (pH 4.5) and bath sonicated for 10 min. The samples were incubated at 37°C for 60 min. The reaction was terminated by extraction in H_2O and $CHCl_3$:CH_3OH:HCl (100:100:1, v/v/v). The lower phase was dried, and samples were resuspended in $CHCl_3$:CH_3OH (1:1, v/v), separated by TLC with $CHCl_3$:CH_3OH:NH_4OH (90:20:0.5, v/v/v), and analyzed with a Fuji Imager.

Human bronchial epithelial cells

Air-liquid interface cultures were mechanically scraped from the Transwell surface and were lysed in 1% Nonidet P40 (NP40) in 150 mM sodium acetate (pH 4.5), kept for 5 min on ice and diluted to 0.1% NP40 in 150 mM sodium acetate (pH 4.5). Lysates were incubated with BODIPY®TR ceramide at 37°C for 30 min, extracted, samples were separated by thin layer chromatography as above and analyzed on a Typhoon fluorescence plate reader.

Acid Ceramidase Western Blots

Mice were sacrificed, and the trachea was removed. Epithelial cells were carefully scraped from the inner surface of the trachea. These cells were pelleted and lysed in 125 mM NaCl, 25 mM Tris HCl (pH 7.4), 10 mM EDTA, 10 mM sodium pyrophosphate, 3% Nonidet P40, and 10 μg/ml aprotinin and leupeptin for 5 min at 4°C. They were then centrifuged at 14,000 rpm. Lysates were added to 5x SDS sample buffer, and proteins were separated by 12.5% SDS-PAGE, blotted, and developed with anti-acid ceramidase antibodies and ECL.

Internalization of FITC-Coupled RGD

WT or CF mice were inhaled with FITC-coupled RGD as above. Tracheal epithelial cells were isolated 30 min later and analyzed by confocal microscopy.

Flow Cytometry Studies

To induce internalization of β1-Integrin or Art1, the intact surface of the trachea was incubated with anti-β1-integrin-antibodies (9EG7), anti-β1-integrin-antibodies (9EG7) that were already immobilized to protein A/G agarose, anti-Art1-antibodies (#sc-20255, Santa Cruz Inc.), anti-ceramide or isotype control antibodies for 30, 60 or 90 min at 37°C. The samples were washed and the surface was then incubated with 1 μg/mL anti-β1-Integrin-(clone MB1.2) or anti-Art1-antibodies (1:100,) for 30 min at 4°C, washed extensively in H/S and the surface was incubated for 30 min with a FITC-coupled secondary antibody. Controls were immediately incubated with 1 μg/mL of each anti-β1-Integrin-(clone MB1.2) or anti-Art1-antibodies for 30 min at 4°C. Acid ceramidase (100 units/mL), N-oleoylethanolamine (0.5 mM) or carmofur (1 μM) were added 15 min prior to the antibodies. C16 ceramide (10 μM) was added to the surface of WT trachea for 30 min at 37°C prior to staining with anti-β1-Integrin-antibodies clone MB1.2. The trachea was finally washed again and epithelial cells were scraped from the surface. To obtain a single cell suspension, the samples were incubated for 5 min with trypsin at 37°C, washed in cold H/S and analyzed on a FASC-Calibur. The mean fluorescence (MFI) intensity of 10 000 signals was determined. In addition, the isolated tracheal surface was infected with 10^4 CFU *P. aeruginosa* strains ATCC 27853 or 762 for 15 or 30 min, washed, incubated with anti-β1-Integrin-antibodies clone MB1.2 and analyzed as above for surface expression of β1-Integrin.

Acid Sphingomyelinase Activity

WT or CF tracheae were infected with 10^4 CFU *P. aeruginosa* for 15 min, washed and tracheal epithelial cells were carefully scraped from the trachea surface. Cells were pelleted, lysed in in 250 μL of a buffer consisting of 250 mM sodium acetate (pH 5.0) and 0.1% NP-40 and sonicated 3-times for 10 s each with a tip sonicator at 4°C. Samples were incubated with 25 nCi per sample [^{14}C]-sphingomyelin (52 mCi/mmol, #NEC 663010UC, Perkin Elmer) for 60 min at 37°C. The substrate was dried prior to use, resuspended in 250 mM sodium acetate (pH 5.0) and 0.1% NP-40, bath sonicated for 10 min and an aliquot of 30 μL was added to the lysates. Samples were extracted in 800 μL chloroform/methanol (2:1, v/v), phases were separated by centrifugation and radioactivity of the aqueous phase was measured by liquid scintillation counting to determine the release of [^{14}C]phosphorylcholine from [^{14}C]sphingomyelin.

Degradation and Uptake Kinetics

Tracheal surface proteins were biotinylated for 30 min using a labeling kit (Pierce) following the instructions of the vendor. Samples were kept in a humidified chamber at 37°C. Samples were then incubated with 100 units/mL acid ceramidase or 1 μg/mL RGD-peptides for 30, 60 or 90 min or left untreated.

To determine degradation of β1-Integrin, the samples were washed after the indicated times, lysed in 125 mM NaCl, 25 mM Tris HCl (pH 7.4), 10 mM EDTA, 10 mM sodium pyrophosphate, 3% Nonidet P40, and 10 μg/ml aprotinin and leupeptin for 5 min at 4°C, insoluble material was pelleted and β1-Integrin was precipitated using Streptavidin-coupled agarose. Blots were developed with anti-β1-Integrin antibodies (clone MB1.2) and HRP-coupled secondary antibodies (Santa Cruz Inc.).

To determine internalization, samples were washed in H/S after biotinylation and furher incubations as indicated and the intact trachea surface was incubated with anti-β1-Integrin antibodies clone MB1.2 for 30 min at 4°C, washed in H/S, lysed as above, immunocomplexes were immobilized using protein A/G agarose, eluted into 1x SDS-sample buffer, separated on SDS-PAGE, blotted and developed with HRP-coupled streptavidin (BioRad). Actin blots were performed from aliquots of the total lysates prior to immunoprecipitation and served as loading controls.

In Vitro Infection of Trachea Surfaces

Trachea were carefully excised and incubated with 200 CFU of *P. aeruginosa* 762, ATCC27853 or 1242 for 60 min. The bacteria were applied in a volume of 3 μL H/S to the surface of the trachea, the trachea was placed in a humidified chamber and incubated for 60 min at 37°C. The samples were then transferred into 100 μL TSB medium, the tissue was homogenized and the samples were plated on LB agar plates. Colonies were counted after overnight growth.

Sphingosine (10 μM), acid ceramidase (100 units/mL), octylglucopyranoside (OGP, 0.01%), anti-sphingosine antibodies (1 μg/mL) or sphingosine kinase (0.001 units) were added 15 min prior to the bacteria and washed off before adding the bacteria. Sphingosine was suspended in 10% OGP. Cells were permeabilized with 0.1% Triton X-100 in H/S for 5 min, washed in H/S, treated with 10 μM sphingosine, 100 units/mL acid ceramidase, 0.01% OGP or left untreated and then infected. PFA (0.25%) was added for 10 min, the samples were then extensively washed, treated with sphingosine, acid ceramidase, OGP as above or left untreated and infected.

Ceramide-β1-Integrin Association

Trachea were exposed, incubated in vitro as indicated, washed in H/S, anti-β1-Integrin antibodies clone MB1.2 (1 μg/mL) were added and incubated at 4°C for 30 min. The samples were washed and disrupted by 3 rounds of sonication with a tip sonicator, 10 s each. Nuclei were pelleted by centrifugation at 800xg for 5 min. The supernatants were collected and anti-β1-Integrin antibodies or isotype control antibodies (rat IgG) were immobilised on protein A/G agarose for 30 min at 4°C, washed 5 times in H/S and subjected to a ceramide kinase assay. To degrade ceramide within the samples, we added 100 units/mL acid ceramidase and incubated at 37°C for 15 min.

Inhalation of Anti-sphingosine Antibodies

Mice inhaled 0.8 mL of 2 μg/ml monoclonal anti-sphingosine antibodies diluted in 0.9% saline via a modified PARI BOY nebulizer device (PARI GmbH). Mice were infected 45 min after inhalation of the anti-sphingosine antibodies with $5x10^7$ CFU of *P. aeruginosa* strain 762 or $1x10^8$ CFU of strains ATCC 27853 or 1242. Bacterial numbers in the lung were determined 6 hr after the infection.

Gr1, *P. aeruginosa*, and Hemalaun Stainings

Lung paraffin sections were dewaxed, blocked as above, immunostained for 45 min with anti-GR1-antibodies (anti-Ly-6G/Ly-6C, #553122, BD) diluted 1:200 in H/S plus 1% FCS. Samples were washed 3-times in PBS plus 0.05% Tween 20, stained for 45 min with anti-*P. aeruginosa* antibodies (1:1000, #AP086, Biotrend) subsequently incubated for 45 min with FITC-coupled anti-rat IgG and Cy3-coupled anti-guinea pig antibodies. Finally, the samples were stained for 5 min with hemalaun. Samples were embedded in mowiol and analyzed on a Leica DMIRE2.

In Vitro Incubation with Sphingosine

A 2 mM stock solution of D-erythro-sphingosine (d18:1, Avanti Polar Lipids) in 10% octylglucopyranoside was sonicated in a bath sonicator for 10 min immediately prior to addition to the bacteria to achieve micelle formation. All *P. aeruginosa* strains were cultured overnight on TSA plates, the *P. aeruginosa* strains 762 and ATCC 27853 were then cultured at an OD of 0.2 for 1 hr in TSB to obtain an early logarithmic growth phase. The mucoid *P. aeruginosa* strains 1242 and 1245 were directly taken from the agar plate. All bacteria were diluted in airway surface liquid (ASL)-buffer consisting of 10 mM HEPES, 63 mM NaCl, 2.4 mM KCl, 1.2 mM $CaCl_2$, 1.9 mM $MgSO_4$, pH 7.2. This buffer mimics the ion composition of the airway surface liquid (Baconnais et al., 1999). Sphingosine at the indicated concentration was then immediately added to 1000 CFU of the *P. aeruginosa* strains 762, ATCC 27853, 1242 or 1245. Samples were incubated for 60 min at 37°C. An aliquot was then plated on LB plates, cultured o/n and the CFUs were counted.

Sphingosine in Tracheal Lavage

Trachea were isolated from WT or CF mice and the tracheal surface washed with 200 μL ice-cold H/S. Samples were then extracted in $CHCl_3/CH_3OH$/1N HCl (100:200:1, v/v/v), the lower phase was dried and resuspended in a detergent solution (7.5% [w/v] n-octyl glucopyranoside, 5 mM cardiolipin in 1 mM diethylenetriamine-pentaacetic acid). The kinase reaction was initiated by addition of 0.001 units sphingosine kinase in 50 mM HEPES (pH 7.4), 250 mM NaCl, 30 mM $MgCl_2$ 1 mM ATP and 10 μCi [^{32}P]γATP. Samples were incubated for 30 min at 37°C with shaking (350 rpm) and processed as above. The volume was calculated from the area of the trachea investigated and the published height of the airway surface liquid film (Tarran et al., 2001).

QUANTIFICATION AND STATISTICAL ANALYSIS

Data are expressed as arithmetic means ± SD. For the comparison of continuous variables from independent groups we used Student's t test for two groups and one-way ANOVA for more than two groups followed by post hoc Student's t tests for all pairwise comparisons applying Bonferroni correction for multiple testing. The p values for the pairwise comparisons were calculated after Bonferroni correction. All values were normally distributed. The statistical details (n-numbers, mean ± SD and tests) are given in the figure legends. The sample size planning for the continuous variables in vivo infection experiments was based on two-sided Wilcoxon-Mann-Whitney tests (software: G*Power Version 3.1.7 of the University of Duesseldorf, Germany). Investigators were blinded for histology experiments and animal identity as described above. All data were quantified using ImageJ and are expressed as arbitrary units (a.u.). In each photo 20 randomly chosen areas corresponding to the luminal membrane of 20 different cells were quantified and averaged with the values obtained in the other photos of the fluorescence microscopy studies. Since the control stainings with Cy3-coupled isotype control antibodies are very weak, we also show the light transmission pictures of hemalaun stainings.

DATA AND SOFTWARE AVAILABILITY

G*Power Version 3.1.7 of the University of Duesseldorf, Germany and ImageJ are public programs available online without charge.

Cell Host & Microbe, Volume *21*

Supplemental Information

β1-Integrin Accumulates in Cystic Fibrosis Luminal Airway Epithelial Membranes and Decreases Sphingosine, Promoting Bacterial Infections

Heike Grassmé, Brian Henry, Regan Ziobro, Katrin Anne Becker, Joachim Riethmüller, Aaron Gardner, Aaron P. Seitz, Joerg Steinmann, Stephan Lang, Christopher Ward, Edward H. Schuchman, Charles C. Caldwell, Markus Kamler, Michael J. Edwards, Malcolm Brodlie, and Erich Gulbins

Supplementary legends

Supplementary Table 1, related to Figure 3B: Quantification of western blot studies in Figure 3B

The western blot studies shown in Fig. 3B were quantified and normalized to actin. The ratio of the signal intensity of β1-integrin to actin is given. Shown is the mean ± SD, n=6, ANOVA, overall p-value <0.001.

Supplementary Table 2, related to 4C: Quantification of western blot studies of Figure 4C

The western blot studies shown in Fig. 4C were quantified and the ratio of the signal intensity of acid ceramidase to actin was calculated. Shown is the mean ± SD, n=4, ANOVA, overall p-value <0.001.

Supplementary Table 3, related to Figures 3 and 4 and Figure S3A: Quantification of the degradation kinetics of β1-integrin

The β1-integrin signals obtained in the western blot studies shown in Fig. S3 were quantified and normalized to actin. Given are the mean values ± SD, n=4, ANOVA, overall p-value <0.001.

Supplementary Table 4, related to Figures 3 and 4 and Fig. S3B: Quantification of the internalization kinetics of β1-integrin

Shown is the quantification of the β1-integrin signal in Fig. S3B normalized to actin. Given are the mean values ± SD, n=4, ANOVA, overall p-value <0.001.

Figure S1, related to Figure 1 and 2: Quantification and control staining

(**A**, **B**) Quantification of the specific and isotype antibody control staining of Fig. 1A is shown in Fig. S1A, of Fig. 1B in Fig. S1B. The fluorescence intensity of 20 random areas of the luminal membrane corresponding to 20 cells per sample was quantified using Image J. We analyzed lung sections from 6 mice, thus, the fluorescence intensities of a total of 120 areas (corresponding to 120 cells) were averaged in panel **A** and **B**. Samples were stained with Cy3-labelled anti-β1-integrin or isotype control rat IgG. An example for the background staining with rat IgG (isotype control for anti-β1-integrin antibodies) followed by Cy3-coupled anti-rat antibodies is displayed.

Shown is the mean ± SD, n=6, t-test. (**C**) The quantification of western blots displayed in Fig. 1C is given. Western blots were analyzed using Image J. Shown is the mean ± SD from 4 independent experiments. Actin blots served to normalize the β1-integrin signal. Significant differences were calculated using ANOVA, overall p-value<0.001. (**D, E**) To exclude a general alteration of membranes in CF samples, we stained lung sections with Cy3-Annexin V, which binds to phosphatidylserine. Shown is a typical result from 6 independent experiments. No differences between wildtype (WT) and CF samples were detected. The intensity of the Annexin V staining was quantified as above. The ratio between the fluorescence intensities of Annexin V and β1-integrin staining confirms the accumulation of β1-integrin in the luminal membrane of CF cells. Shown are typical examples and the mean ± SD, n=6, ANOVA, overall p<0.001. (**F**) Lung sections from WT and CF mice were stained with anti-β2-integrin antibodies followed by Cy3-labelled secondary antibodies. The staining is mainly in the alveoli and does not differ between WT and CF samples. Shown is a typical result from 6 independent experiments. (**G**) The figure illustrates the anatomical structure of the epithelial cell layer with the lateral membranes in white color and the luminal membranes in yellow. The panel is from Figure 1A.
(**H-J**) The quantification of the specific and isotype antibody control staining displayed in Fig. 2A is shown in Fig. S1H, of Fig. 2B in Fig. S1I, of Fig. 2D in Fig. S1J. An example for the background staining with mouse IgM followed by Cy3-coupled anti-mouse IgM antibodies (isotype antibody control for anti-ceramide and anti-sphingosine antibodies) is displayed in Fig. S1H. Shown is the mean ± SD, n=6 (H-I), n=4 (J), ANOVA, overall p-value <0.001.

Figure S2, related to Figures 2 and 3: Quantifications and the effect of internalization of the gp-linked protein Art1 on surface β1-integrin, ceramide and sphingosine
(**A-D**) The quantification of the specific and isotype antibody control staining displayed in Fig. 2E is given in Fig. S2A, of Fig. 3A and D in Fig. S2B, of Fig. 4A in Fig. S2C, of Fig. 4E in Fig. S2D. The fluorescence intensities were determined as in Fig. S1A. An example for the background staining with mouse IgG (isotype antibody control for anti-acid ceramidase antibodies) is demonstrated in Fig. S2C. Shown is the mean ± SD, n=4 (A) or n=6 (B-D), ANOVA, overall p-value <0.001.
(**E**) The trachea surface was incubated with anti-Art1 antibodies for 30 min at 37°C or

left untreated, washed and incubated with anti-Art1 antibodies or isotype controls for an additional 30 min at 4°C. The epithelial cells were then scraped from the surface of the trachea, incubated with FITC-coupled secondary antibodies and surface staining of Art1 was determined by flow cytometry. The results show that incubation with anti-Art-1 antibodies did not alter surface β1-integrin. Shown are the mean ± SD of the mean fluorescence values of 3 independent experiments. Statistical differences were analyzed by ANOVA. The decreased mean fluorescence corresponds very likely to an internalization of Art1. (**E-G**) Internalization of Art1 does not change surface expression of β1-integrin in CF cells as determined by flow cytometry, surface ceramide and surface sphingosine as determined by in situ kinase assays. Given are the mean ± SD from 3 independent experiments, ANOVA, overall p-value <0.001.

Figure S3, related to Figures 3 and 4: Kinetics of β1-integrin degradation, internalization and effects on acid ceramidase activity, surface ceramide and sphingosine

(**A and B**) Degradation kinetics of surface β1-integrin: The surface of the trachea of wildtype (WT) and CF mice was biotinylated, washed and cells were lysed after the indicated time of incubation with RGD-peptides. The samples were lysed, precipitated using Streptavidin-coupled agarose and blotted with anti-β1-Integrin antibodies (clone MB1.2) and HRP-coupled secondary antibodies (Santa Cruz Inc.). β1-integrin is rapidly degraded in WT epithelial cells, but remains stable in CF epithelial cells. Displayed is a typical result from 4 independent studies. The quantification is given in supplementary Table 3.

(**B**) Uptake kinetics of surface β1-integrin: The surface of tracheae was biotinylated, washed and the samples were incubated for 0, 30, 60 and 90 min at 37°C in the presence or absence of acid ceramidase (Ac) or binding of RGD-peptides. Surface integrin was then bound to anti-β1-Integrin antibodies clone MB1.2 for 30 min at 4°C, the samples were lysed, precipitated using protein A/G agarose and developed with HRP-coupled streptavidin. Consumption of ceramide by Ac or binding of RGD-peptides allowed rapid internalization of biotinylated surface β1-Integrin in CF cells. Shown is a typical result from 4 independent studies.

(**C, D**) Infection of tracheal epithelial cells from WT and CF mice with *P. aeruginosa* strains 762, ATCC 27853 or 1242 for 15 min results in an activation of the acid

sphingomyelinase (Asm) as determined by consumption of [^{14}C]sphingomyelin (**C**), but has almost no effect on the surface expression of β1-Integrin in CF cells determined by flow cytometry analysis after binding of FITC-coupled anti-β1-Integrin antibodies to the cells (**D**). Shown are the mean values ± SD, n=5, ANOVA, overall p-value <0.001.

(E-H) Addition of anti-β1-integrin antibodies 9EG7 as β1-integrin ligand to the intact surface of the trachea isolated from WT and CF mice results in internalization of β1-integrin (**E**), increased activity of acid ceramidase (**F**), reduction of surface ceramide (**G**) and an increase of surface sphingosine (**H**). Immobilization of the anti-β1-integrin 9EG7 antibodies to agarose beads blocking its internalization prevented reduction of ceramide and the increase of sphingosine in CF cells. Inhibition of the acid ceramidase by N-oleoylethanolamine or carmofur did not affect internalization of β1-integrin upon antibody binding (**E**), but prevented the consumption of ceramide and the generation of sphingosine after incubation with anti-β1-integrin 9EG7 antibodies. Internalization was determined by flow cytometry after staining the still intact surface with FITC-coupled anti-β1-integrin antibodies clone MB1.2 followed by isolation of single epithelial cells. Surface acid ceramidase activity was determined by measuring the consumption of [^{14}C]ceramide added to the tracheal surface, surface ceramide and sphingosine were measured by in situ kinase assays. Shown are the mean ± SD of the mean fluorescence of the flow cytometry studies (background with isotype controls antibodies was subtracted), the enzyme activities or the amounts of ceramide and sphingosine from 4 independent experiments. Statistical differences were determined by ANOVA, overall p-value <0.001.

Figure S4, related to Figure 4 and 5: Surface β1-integrin in CF epithelial cells physically interacts with ceramide and determines pulmonary *P. aeruginosa* infections

(**A**) Immunoprecipitation of β1-integrin from CF cells co-precipitated ceramide, while no ceramide was detected in the immunoprecipitates from wildtype (WT) cells or in control immunoprecipitates from CF cells. Treatment of the surface of CF trachea with acid ceramidase for 15 min at 37°C after binding of the anti-β1-integrin antibodies complexed to protein A/G agarose but prior to lysis reduced the amount of ceramide bound to β1-integrin-immunoprecipitates. In addition, incubation with anti-ceramide antibodies that have been previously shown to displace receptors such as

CD95 from ceramide-enriched membrane domains (Grassmé et al., 2000, Rotolo et al., 2012) either by simple steric competition or by a higher affinity of the antibody to ceramide, abrogated the association of ceramide with β1-integrin. Isotype control IgM antibodies (Ab) were without effect on the co-precipitation of ceramide with β1-integrin. Incubation of the surface of the trachea from WT animals with 10 μM C_{16}-ceramide resulted in co-precipitation of ceramide with β1-integrin. Ceramide was determined by ceramide kinase assays. (**B**) Incubation with anti-ceramide antibodies resulted in rapid internalization of β1-integrin as measured by flow cytometry after staining intact tracheal surfaces with FITC-coupled anti-β1-integrin antibodies. Conversely, incubation of WT trachea with 10 μM C16 ceramide resulted in surface trapping and expression of β1-integrin.

Shown are the mean ± SD of ceramide or the mean fluorescence of the stained cells (background with isotype controls antibodies was subtracted) from 4 independent experiments, ANOVA, overall p-value <0.001.

(**C**) We intranasally infected CF mice with 5×10^6 CFU of *P. aeruginosa* strain 762. Please note that the scale bars in Figure S4C and Figure 5A are 10-fold different. These studies showed that the number of bacteria in the lung correlates linearly with the intranasally applied CFU. Inhalation of anti-β1-integrin antibodies or RGD peptides rescued each mouse strain from infection with *P. aeruginosa*. Control peptides or isotype-matched irrelevant antibodies did not alter the massive infection of these mouse strains. Mean ± SD, n=6, ANOVA, overall p<0.001.

(**D, E**) Inhalation of nebulized anti-β1-integrin antibodies (Ab) or RGD peptides 1 hr prior infection intranasal application with 10^5 (**D**) or 10^8 (**E**) CFU *P. aeruginosa* strain ATCC 27853 greatly reduced the number of bacteria that reach the lower part of the trachea (**D**) and thereby prevents pulmonary infection after (**E**). Shown are the mean ± SD of pulmonary *P. aeruginosa* CFUs, n=6. Statistical differences were determined by t-test or ANOVA, overall p-value <0.001 in all subpanels.

(**F**) Survival studies after *P. aeruginosa* infection revealed that acute infection with *P. aeruginosa* strains 762 or ATCC 27853 resulted in death of CF mice within 96 hrs, which was abrogated by inhalation of acid ceramidase, sphingosine or RGD peptides. The dose of the mucoid strains used above to obtain a (sub-)chronic infection was not high enough to induce a lethal infection. We therefore used a higher infection dose (1×10^8 CFU of *P. aeruginosa* 1242 and 1245). This infection resulted in death of CF mice, which was prevented by inhalation of Ac or sphingosine. The

panel shows Kaplan Meyer-survival curves of CF^{MHH} or WT mice infected with 5×10^7 CFU of *P. aeruginosa* strain 762 or 10^8 CFU of ATCC strains 27853, 1242 and 1245. $p<0.001$ for all treated or WT curves compared to corresponding untreated curves, log-rank test.

(**G**) WT mice were inhaled with 2 μg/ml monoclonal anti-sphingosine antibodies diluted in 0.9% saline or as control with saline only and infected 45 min later with $5x10^7$ CFU of *P. aeruginosa* strain 762 or $1x10^8$ CFU of strains ATCC27853 or 1242, the lungs were removed 6 hrs later, homogenized, aliquots were plated on TSA plates, grown o/n and colonies were counted. Given are the mean ± SD of pulmonary *P. aeruginosa* CFUs, n=5. Statistical differences were determined by t-test or ANOVA, overall p-value <0.001 in all subpanels.

Figure S5*, related to Figure 5: Inhalation of sphingosine or acid ceramidase rapidly kills pulmonary mucoid and non-mucoid *P. aeruginosa*

Mice were infected with $5x10^7$ CFU *P. aeruginosa* 762 and inhaled with sphingosine (SPH) or acid ceramidase (Ac) 1 hr after infection. Lungs were removed 6 hrs (**A**) or 48 hrs (**C**) after infection. Infection with *P. aeruginosa* 1242 was performed once daily at $1x10^7$ CFUs for 5 days, mice were inhaled with sphingosine or acid ceramidase 1 hr (**B**) or 48 hrs (**C**) after the last infection and sacrificed 5 hrs (**B**) or 48 hrs after inhalation. Lungs were removed, fixed in 4% paraformaldehyde, embedded in paraffin, sections were performed and stained with FITC-labelled anti-Gr1 (Ly6C/G) and Cy3-labelled anti-*P. aeruginosa* antibodies and analyzed by confocal microscopy. The sections obtained from mice 48 hrs after treatment were only stained with FITC-labelled anti-Gr1 (Ly6C/G), since the number of bacteria in the lung at this time point is too low to be detected by staining 5 μm sections with *P. aeruginosa* antibodies. The results show that sphingosine and acid ceramidase rapidly kill the bacteria within a few hours followed by resolution of the inflammation over the next few days, while untreated CF mice show massive amounts of bacteria and GR1-positive myeloid cells 6 hrs and even 96 hrs after infection. Panel **D** shows the control staining of uninfected mice. The first time given in the headlines of the figures refers to the time of treatment after the last infection, the 2nd time to the time of the removal of the lung after infection. Data are representative for 6 experiments with very similar results.

Figure S6, related to Figure 5: Surface sphingosine kills pulmonary mucoid and non-mucoid *P. aeruginosa* at concentrations that are present in the tracheal surface fluid

(**A**) The non-mucoid *P. aeruginosa* strains 762 and ATCC 27853 and the mucoid strains 1242 and 1245 were incubated with increasing concentrations of sphingosine in a buffer, which mimics the ion composition of the airway surface liquid (Borowitz et al, 1999). Bacteria (1000 CFU) were incubated for 60 min with the indicated concentrations of sphingosine, aliquots were plated on TSB plates and CFU were counted on the next day. Shown is the mean ± SD from 4 independent experiments, ANOVA, overall p-value <0.001. (**B**) Mice were infected with $1x10^8$ CFU *P. aeruginosa* strains ATCC 27853 or 1242 or left uninfected, in addition, mice were inhaled with acid ceramidase (Ac) or sphingosine (SPH). Tracheal surface fluid was isolated, extracted and the sphingosine concentration was determined using a sphingosine kinase assay. Shown are the mean ± SD from 4 independent measurements, ANOVA, overall p-value <0.001.

(**C-E**) To test whether sphingosine regulated by β1-integrin is sufficient to serve as first line of defense in the upper airways and the bronchi killing bacteria without an inflammatory response, we infected the intact epithelial cell layer of isolated trachea from CF or WT mice with *P. aeruginosa* and determined killing of *P. aeruginosa*. Trachea from WT or CF mice were isolated and incubated with anti-sphingosine (SPH) antibodies, sphingosine (SPH), acid ceramidase (Ac), sphingosine kinase, or octylglucopyranoside (OGP) as control. Further, the trachea was fixed for 10 min with 0.25% paraformaldehyde (PFA) or permeabilized for 5 min with 0.1% Triton X-100. The tracheae were then infected with 200 CFU of *P. aeruginosa* strains 762, ATCC 27853 or 1242 for 60 min, homogenized and the samples was spotted onto TSA plates. Bacteria were counted after 24 hrs growth. The results show that WT tracheal epithelial cells kill *P. aeruginosa*, while CF epithelial cells fail. The reconstitution of sphingosine restores killing by CF epithelial cells. Neutralization of sphingosine by anti-sphingosine antibodies or phosphorylation of sphingosine by sphingosine kinase prevents killing of the bacteria by WT cells or treated CF cells. The permeabilization studies served to exclude a signalling function of sphingosine.

(**F**) In situ sphingosine kinase assays were performed to confirm that the treatment with acid ceramidase (Ac) or sphingosine (SPH) reconstituted sphingosine on the

surface of CF trachea to physiological levels. Displayed are the mean ± SD from 5 independent measurements, ANOVA, overall p-value <0.001.

Figure S7, related to Figures 6 and 7: Ectopic expression of β1-integrins causes ceramide accumulation in human airway CF cells

Ceramide is severely upregulated on the luminal pole of human CF bronchial epithelial cells (**A**), nasal epithelial cells (**B**) and nasal polyps (**C**). Normalization of ectopic β1-integrin expression or incubation of freshly isolated epithelial cells with acid ceramidase reduced ceramide levels in CF cells (**B**). Sections or cells were stained as indicated with Cy3-labelled anti-ceramide antibodies. Pre-incubations were performed with RGD-peptides, HUTS-4 anti-β1-integrin antibodies (α-Int.-Ab) or acid ceramidase (Ac). The luminal membrane is indicated by arrows. Shown are representative results from each 5 CF or 4 healthy individuals (all lung sections and freshly isolated epithelial cells) or each 4 CF or healthy persons (nasal polyps). (**D**) CF or healthy control nasal epithelial cells were counted, aliquoted and infected with *P. aeruginosa* strains 762 or ATCC 27853 at a multiplicity of infection of 1 cell : 0.5 bacteria. The samples were incubated at 37°C for 60 min, plated on LB agar plates, cultured overnight and *P. aeruginosa* colonies were counted. CF cells have a reduced capability to kill *P. aeruginosa*, which is corrected by pre-incubation with anti-β1-integrin antibodies (HUTS-4), RGD-peptides, acid ceramidase or sphingosine. (**E**) Acid ceramidase activity is reduced in primary bronchial epithelial cells from people with CF (n=4) compared to non-CF individuals (n=5).

The fluorescence intensities in the luminal membrane of all fluorescence microscopy studies were quantified using Image J and are given in arbitrary units (a.u.). Given are the mean ± SD, n = 4 or 5, p-values are given in the figures, t-test for comparison of 2 values and ANOVA and post-hoc Student´s t-tests for all pairwise comparisons applying Bonferroni correction for multiple comparisons. Overall p-value for ANOVA is <0.001 in all panels. E = epithelial cell layer.

Supplementary Table 1

Ratio β-Integrin-signal intensity/Actin signal intensity [a.u], related to Fig. 3B

WT	0.014 ± 0.004
CF^{MHH}	1.23 ± 0.092 ($p<0.001$ compared to WT)
CF^{MHH} + RGD	0.051 ± 0.032 ($p<0.001$ compared to CF^{MHH})
CF^{MHH} + ctrl. Peptide	1.23 ± 0.092 ($p<0.001$ compared to CF^{MHH} + RGD)
CF^{MHH} + anti-β1-Integrin-antibody	0.123 ± 0.022 ($p<0.001$ compared to CF^{MHH})
CF^{MHH} + ctrl. Antibody	1.23 ± 0.095 ($p<0.001$ compared to CF^{MHH} + anti-β1-Int)
WT	0.013 ± 0.004
$Cftr^{-/-}$	1.19 ± 0.059 ($p<0.001$ compared to WT)
$Cftr^{-/-}$ + RGD	0.030 ± 0.013 ($p<0.001$ compared to $Cftr^{-/-}$)
$Cftr^{-/-}$ + ctrl. Peptide	1.17 ± 0.161 ($p<0.001$ compared to $Cftr^{-/-}$ + RGD)
$Cftr^{-/-}$ + anti-β1-Integrin-antibody	0.090 ± 0.046 ($p<0.001$ compared to $Cftr^{-/-}$)
$Cftr^{-/-}$ + ctrl. Antibody	1.26 ± 0.135 ($p<0.001$ compared to $Cftr^{-/-}$ + anti-β1 Int)
unspecific	0.005 ± 0.002

Supplementary Table 2

Ratio acid ceramidase (α-subunit)-signal intensity/Actin signal intensity [a.u.], related to Fig. 4C

WT	1.47 ± 0.078
$Cftr^{-/-}$	0.027 ± 0.011 ($p<0.001$ compared to WT)
$Cftr^{-/-}$ + RGD	1.48 ± 0.112 ($p<0.001$ compared to $Cftr^{-/-}$)
$Cftr^{-/-}$ + ctrl. Peptide	0.033 ± 0.012 ($p<0.001$ compared to $Cftr^{-/-}$ + RGD)
$Cftr^{-/-}$ + anti-β1-Integrin	1.47 ± 0.127 ($p<0.001$ compared to $Cftr^{-/-}$)
$Cftr^{-/-}$ + ctrl. Antibody	0.042 ± 0.015 ($p<0.001$ compared to $Cftr^{-/-}$ + anti-β1-Integrin)

Ratio acid ceramidase (β-subunit)-signal intensity/Actin signal intensity [a.u.], related to Fig. 4C

WT	0.935 ± 0.083
$Cftr^{-/-}$	0.021 ± 0.007 ($p<0.001$ compared to WT)
$Cftr^{-/-}$ + RGD	0.840 ± 0.062 ($p<0.001$ compared to $Cftr^{-/-}$)
$Cftr^{-/-}$ + ctrl. Peptide	0.043 ± 0.021 ($p<0.001$ compared to $Cftr^{-/-}$ + RGD)
$Cftr^{-/-}$ + anti-β1-Integrin	0.755 ± 0.037 ($p<0.001$ compared to $Cftr^{-/-}$)
$Cftr^{-/-}$ + ctrl. Antibody	0.030 ± 0.001 ($p<0.001$ compared to $Cftr^{-/-}$ + anti-β1-Integrin)

Supplementary Table 3

Ratio β-Integrin-signal intensity/Actin signal intensity [a.u], related to Figures 3 and 4 and Figure S3A

WT-0 min	0.65 ± 0.072
WT-60 min	0.09 ± 0.026 ($p<0.001$ compared to WT-0 min)
WT-90 min	0.06 ± 0.001 ($p<0.001$ compared to WT-0 min)
$Cftr^{-/-}$-0 min	1.08 ± 0.1 ($p<0.001$ compared to WT-0 min)
$Cftr^{-/-}$-60 min	0.85 ± 0.06 ($p<0.001$ compared to WT-60 min)
$Cftr^{-/-}$-90 min	0.75 ± 0.07 ($p<0.001$ compared to WT-90 min)
$Cftr^{-/-}$ + RGD-0 min	0.96 ± 0.04 ($p<0.001$ compared to WT-0 min)
$Cftr^{-/-}$ + RGD-60 min	0.39 ± 0.05 ($p<0.001$ compared to $Cftr^{-/-}$-60 min; $p<0.0064$ compared to WT-60 min)
$Cftr^{-/-}$ + RGD-90 min	0.18 ± 0.05 ($p<0.001$ compared to $Cftr^{-/-}$-60 min; $p<0.001$ compared to WT-90 min)

Supplementary Table 4

Ratio β-Integrin-signal intensity/Actin signal intensity [a.u], related to Figures 3 and 4 and Figure S3B

$Cftr^{-/-}$-0 min	0.49 ± 0.030
$Cftr^{-/-}$-30 min	0.48 ± 0.040
$Cftr^{-/-}$-60 min	0.45 ± 0.027
$Cftr^{-/-}$-90 min	0.36 ± 0.031 (p<0.001 compared to $Cftr^{-/-}$-0 min)
CF^{MHH}-0 min	0.48 ± 0.022
CF^{MHH}-90 min	0.47 ± 0.017
$Cftr^{-/-}$ + RGD-30 min	0.26 ± 0.013 (p<0.001 compared to $Cftr^{-/-}$-30 min)
$Cftr^{-/-}$ + RGD-60 min	0.20 ± 0.017 (p<0.001 compared to $Cftr^{-/-}$-60 min)
$Cftr^{-/-}$ + RGD-90 min	0.06 ± 0.018 (p<0.001 compared to $Cftr^{-/-}$-90 min)
$Cftr^{-/-}$ + Ac-30 min	0.26 ± 0.001 (p<001 compared to $Cftr^{-/-}$-30 min)
$Cftr^{-/-}$ + Ac-60 min	0.16 ± 0.016 (p<0.001 compared to $Cftr^{-/-}$-60 min)
$Cftr^{-/-}$ + Ac-90 min	0.03 ± 0.008 (p<0.001 compared to $Cftr^{-/-}$-90 min)

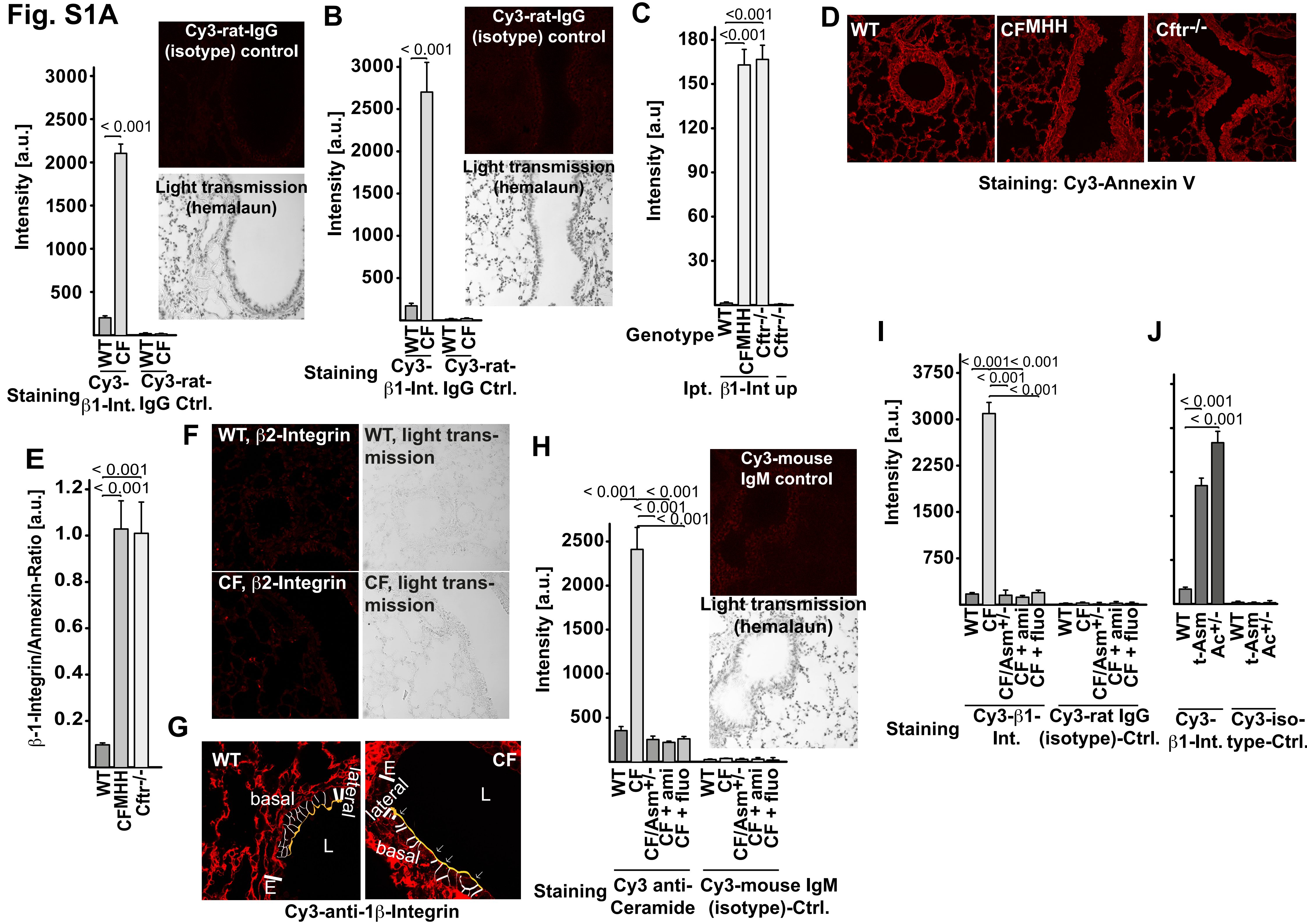
Fig. S1A
Cy3-rat-IgG (isotype) control
Light transmission (hemalaun)
Intensity [a.u.]
< 0.001
WT CF WT CF
Staining
Cy3-β1-Int.
Cy3-rat-IgG Ctrl.
B
Cy3-rat-IgG (isotype) control
Light transmission (hemalaun)
C
<0.001
Genotype
WT CFMHH Cftr-/- Cftr-/-
Ipt. β1-Int up
D
WT
CFMHH
Cftr-/-
Staining: Cy3-Annexin V
E
β-1-Integrin/Annexin-Ratio [a.u.]
F
WT, β2-Integrin
WT, light transmission
CF, β2-Integrin
CF, light transmission
G
basal
lateral
E
L
Cy3-anti-1β-Integrin
H
Cy3-mouse IgM control
Cy3 anti-Ceramide
Cy3-mouse IgM (isotype)-Ctrl.
CF/Asm+/- CF + ami CF + fluo
I
Cy3-β1-Int.
Cy3-rat IgG (isotype)-Ctrl.
J
t-Asm Ac+/-
Cy3-β1-Int.
Cy3-isotype-Ctrl.

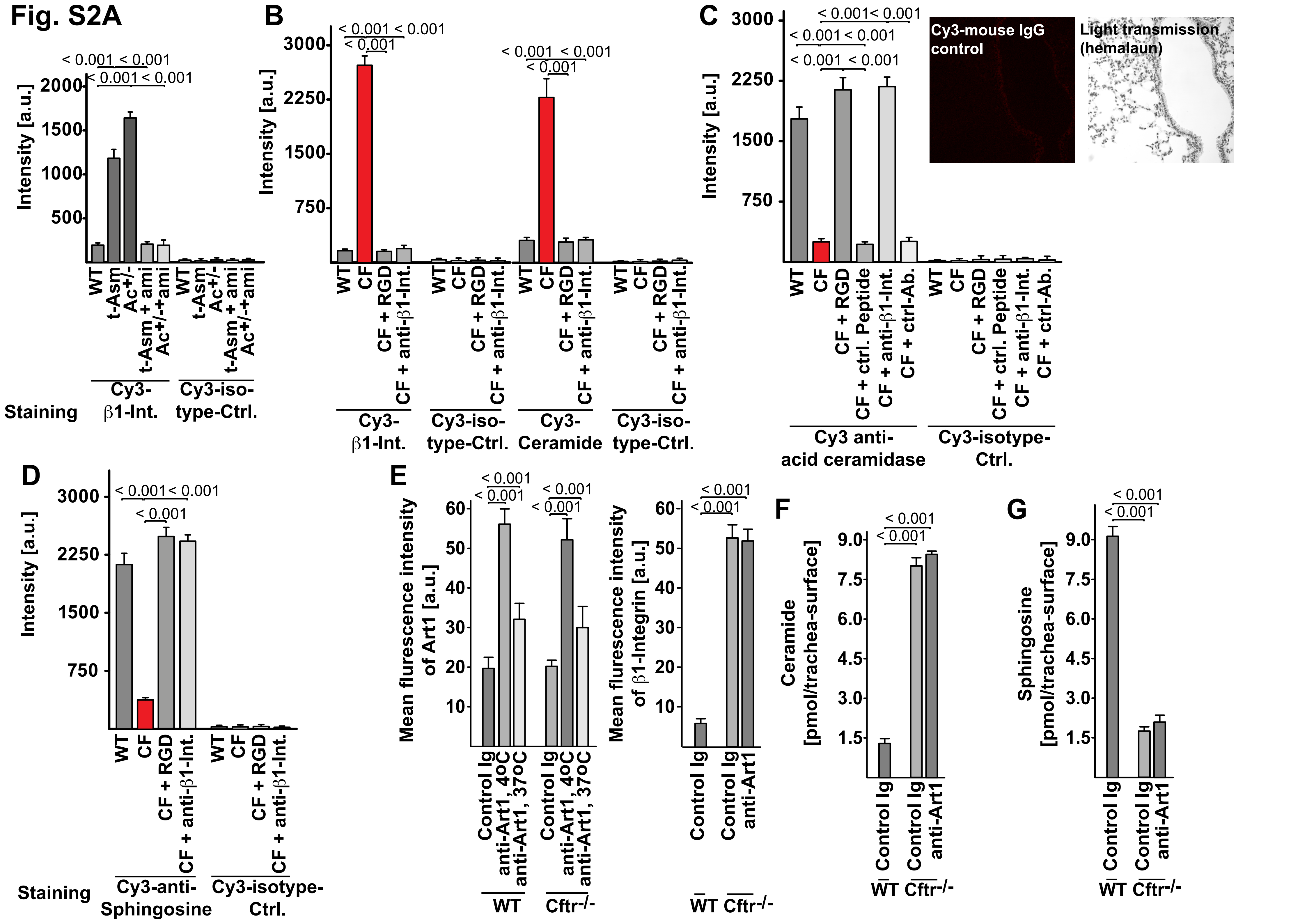
Fig. S2A
B
C
D
E
F
G
Intensity [a.u.]
Staining
Cy3-β1-Int.
Cy3-iso-type-Ctrl.
Cy3-Ceramide
Cy3 anti-acid ceramidase
Cy3-isotype-Ctrl.
Cy3-mouse IgG control
Light transmission (hemalaun)
Cy3-anti-Sphingosine
WT
CF
t-Asm
Ac+/-
t-Asm + ami
Ac+/-+ami
CF + RGD
CF + anti-β1-Int.
CF + ctrl. Peptide
CF + anti-β1-Int.
CF + ctrl-Ab.
Mean flurescence intensity of Art1 [a.u.]
Mean flurescence intensity of β1-Integrin [a.u.]
Control Ig
anti-Art1, 4oC
anti-Art1, 37oC
anti-Art1
Cftr-/-
Ceramide [pmol/trachea-surface]
Sphingosine [pmol/trachea-surface]
< 0.001

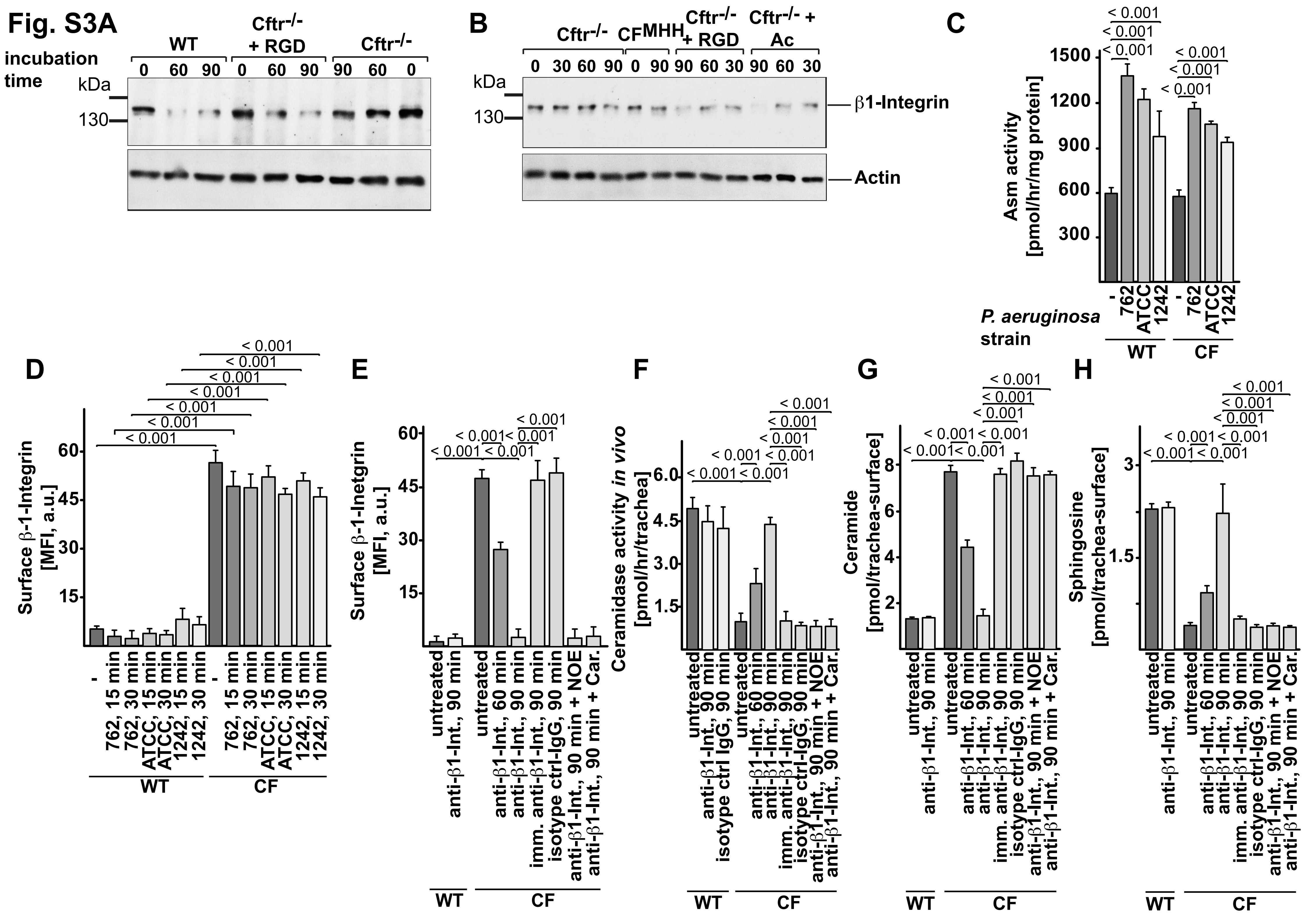
Fig. S3A
incubation time
WT
Cftr-/- + RGD
Cftr-/-
kDa
130
B
CFMHH
Cftr-/- + Ac
β1-Integrin
Actin
C
Asm activity [pmol/hr/mg protein]
P. aeruginosa strain
762
ATCC
1242
WT
CF
D
Surface β-1-Integrin [MFI, a.u.]
E
Surface β-1-Inetgrin [MFI, a.u.]
untreated
anti-β1-Int., 90 min
anti-β1-Int., 60 min
imm. anti-β1-Int., 90 min
isotype ctrl-IgG, 90 min
anti-β1-Int., 90 min + NOE
anti-β1-Int., 90 min + Car.
F
Ceramidase activity in vivo [pmol/hr/trachea]
isotype ctrl IgG, 90 min
G
Ceramide [pmol/trachea-surface]
H
Sphingosine [pmol/trachea-surface]
< 0.001

Fig. S4

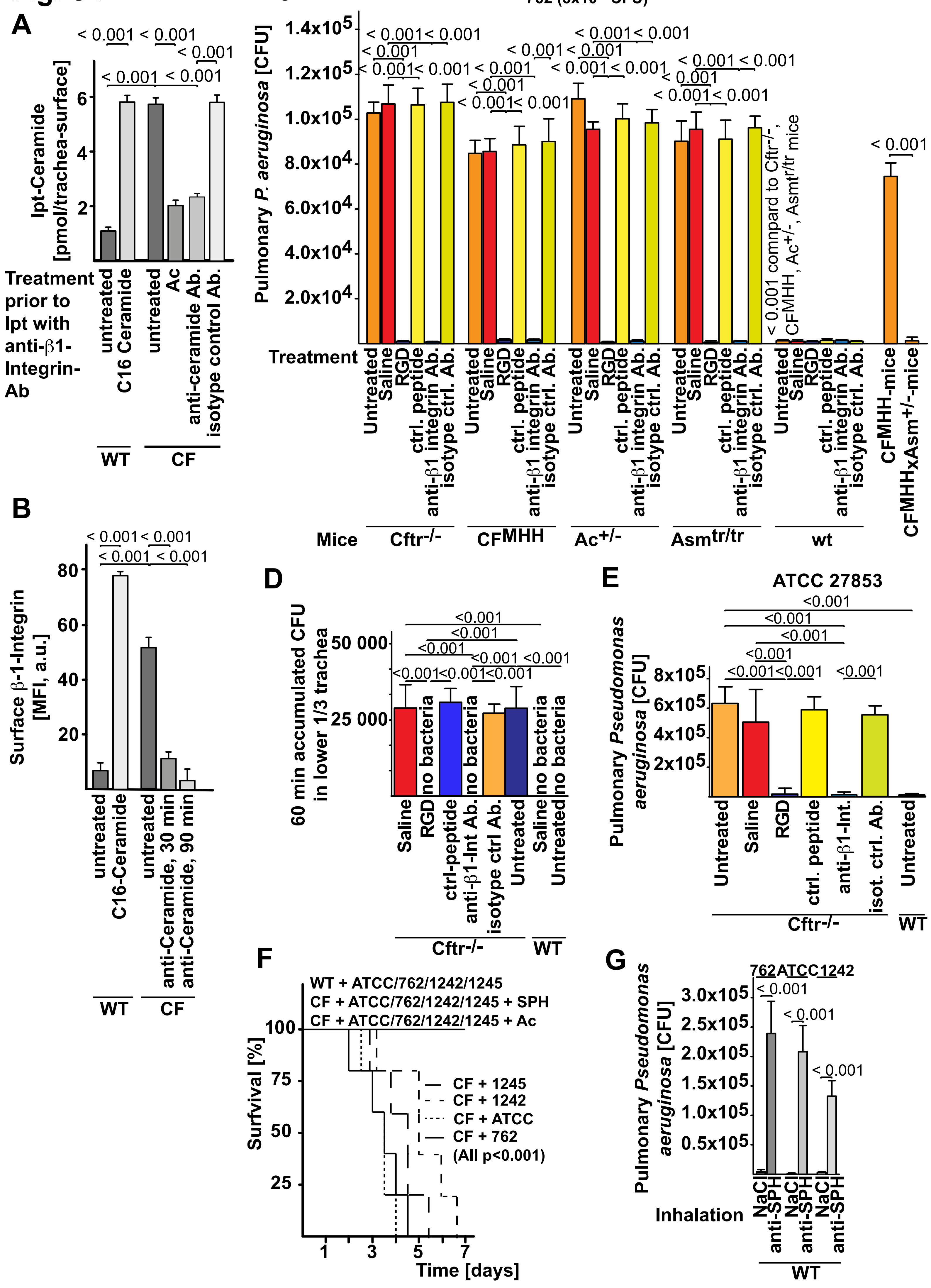
A
Ipt-Ceramide [pmol/trachea-surface]
Treatment prior to Ipt with anti-β1-Integrin-Ab
untreated
C16 Ceramide
untreated
Ac
anti-ceramide Ab.
isotype control Ab.
WT
CF
< 0.001
B
Surface β-1-Integrin [MFI, a.u.]
untreated
C16-Ceramide
untreated
anti-Ceramide, 30 min
anti-Ceramide, 90 min
WT
CF
< 0.001
C
762 (5x10^6 CFU)
Pulmonary P. aeruginosa [CFU]
Treatment
Untreated
Saline
RGD
ctrl. peptide
anti-β1 integrin Ab.
isotype ctrl. Ab.
Mice
Cftr-/-
CFMHH
Ac+/-
Asmtr/tr
wt
CFMHH-mice
CFMHHxAsm+/--mice
< 0.001 compard to Cftr-/-, CFMHH, Ac+/-, Asmtr/tr mice
< 0.001
D
60 min accumulated CFU in lower 1/3 trachea
Saline
RGD
no bacteria
ctrl-peptide
anti-β1-Int Ab.
isotype ctrl Ab.
Untreated
Cftr-/-
WT
<0.001
E
ATCC 27853
Pulmonary Pseudomonas aeruginosa [CFU]
Untreated
Saline
RGD
ctrl. peptide
anti-β1-Int.
isot. ctrl. Ab.
Cftr-/-
WT
<0.001
F
WT + ATCC/762/1242/1245
CF + ATCC/762/1242/1245 + SPH
CF + ATCC/762/1242/1245 + Ac
Survival [%]
Time [days]
CF + 1245
CF + 1242
CF + ATCC
CF + 762
(All p<0.001)
G
762 ATCC 1242
Pulmonary Pseudomonas aeruginosa [CFU]
Inhalation
NaCl
anti-SPH
WT
< 0.001

Fig. S5

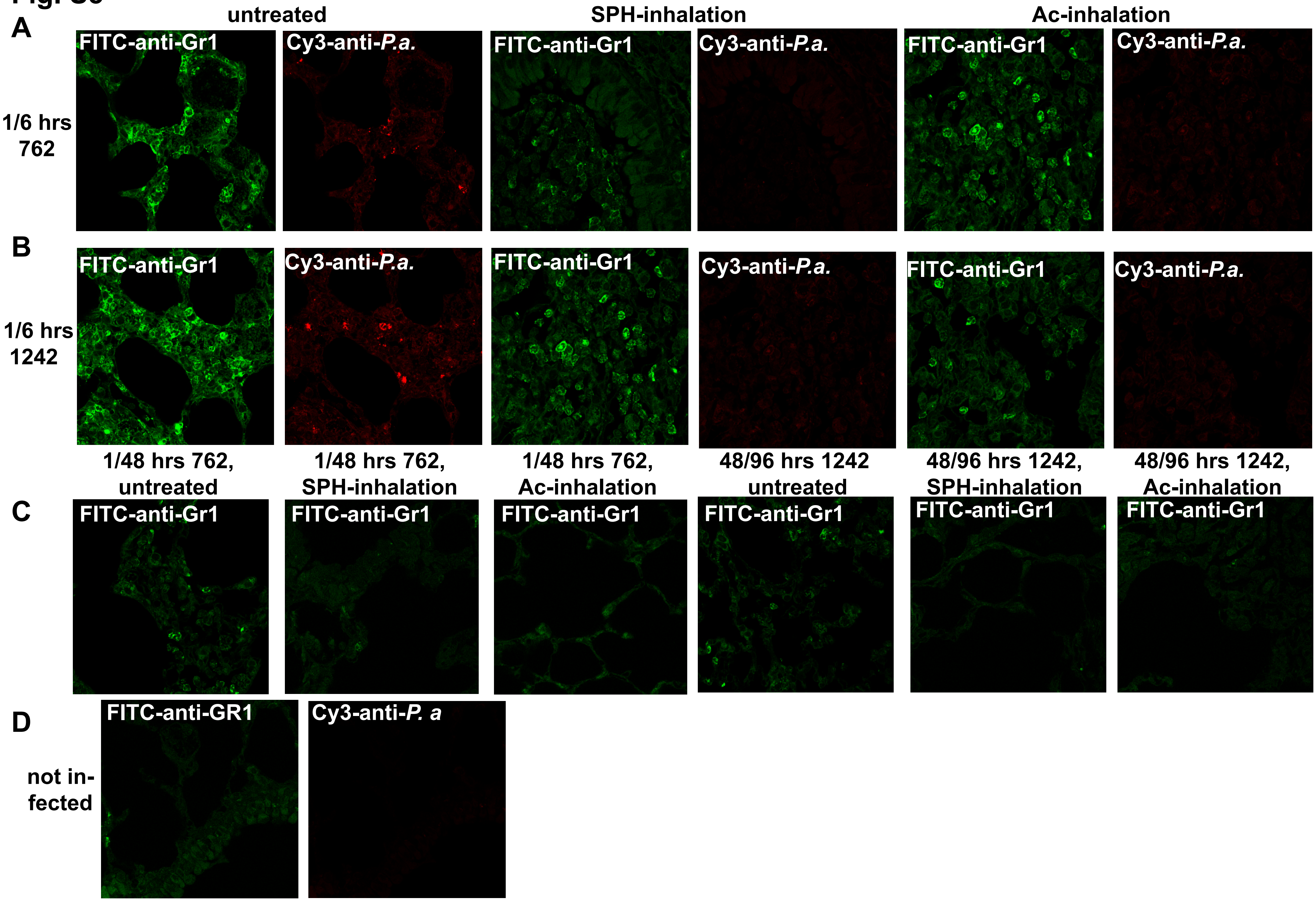

untreated
SPH-inhalation
Ac-inhalation
A
1/6 hrs
762
FITC-anti-Gr1
Cy3-anti-*P.a.*
B
1/6 hrs
1242
1/48 hrs 762, untreated
1/48 hrs 762, SPH-inhalation
1/48 hrs 762, Ac-inhalation
48/96 hrs 1242 untreated
48/96 hrs 1242, SPH-inhalation
48/96 hrs 1242, Ac-inhalation
C
D
not in-fected
FITC-anti-GR1
Cy3-anti-*P. a*

Fig. S6

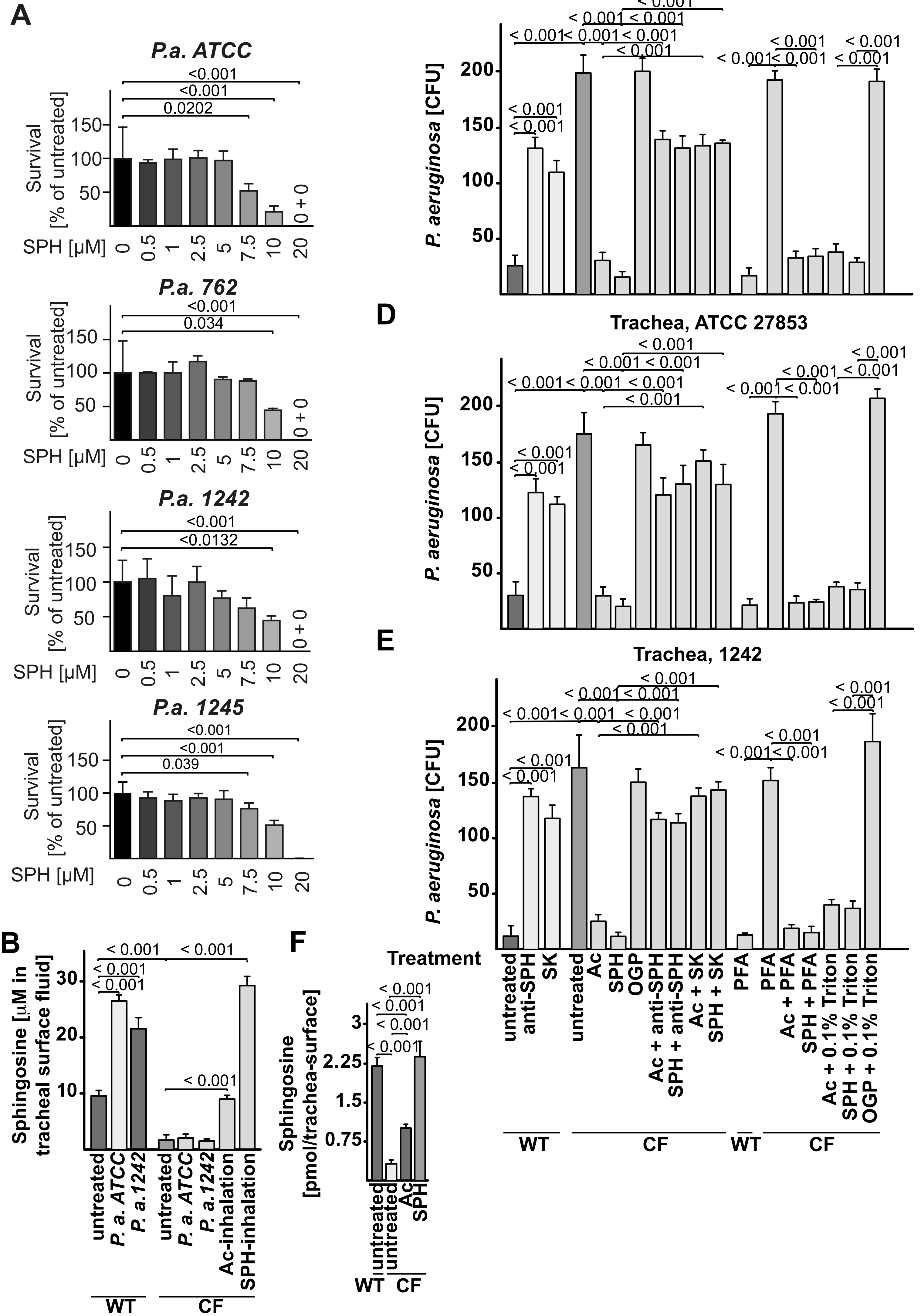

A
P.a. ATCC
P.a. 762
P.a. 1242
P.a. 1245
Survival [% of untreated]
SPH [µM]
0 0.5 1 2.5 5 7.5 10 20
0 + 0
B
Sphingosine [µM in tracheal surface fluid]
untreated
P. a. ATCC
P. a.1242
untreated
P. a. ATCC
P. a.1242
Ac-inhalation
SPH-inhalation
WT
CF
C
Trachea, 762
D
Trachea, ATCC 27853
E
Trachea, 1242
P. aeruginosa [CFU]
< 0.001
untreated
anti-SPH
SK
untreated
Ac
SPH
OGP
Ac + anti-SPH
SPH + anti-SPH
Ac + SK
SPH + SK
PFA
PFA
Ac + PFA
SPH + PFA
Ac + 0.1% Triton
SPH + 0.1% Triton
OGP + 0.1% Triton
WT
CF
WT
CF
F
Treatment
Sphingosine [pmol/trachea-surface]
untreated
untreated
Ac
SPH
WT
CF

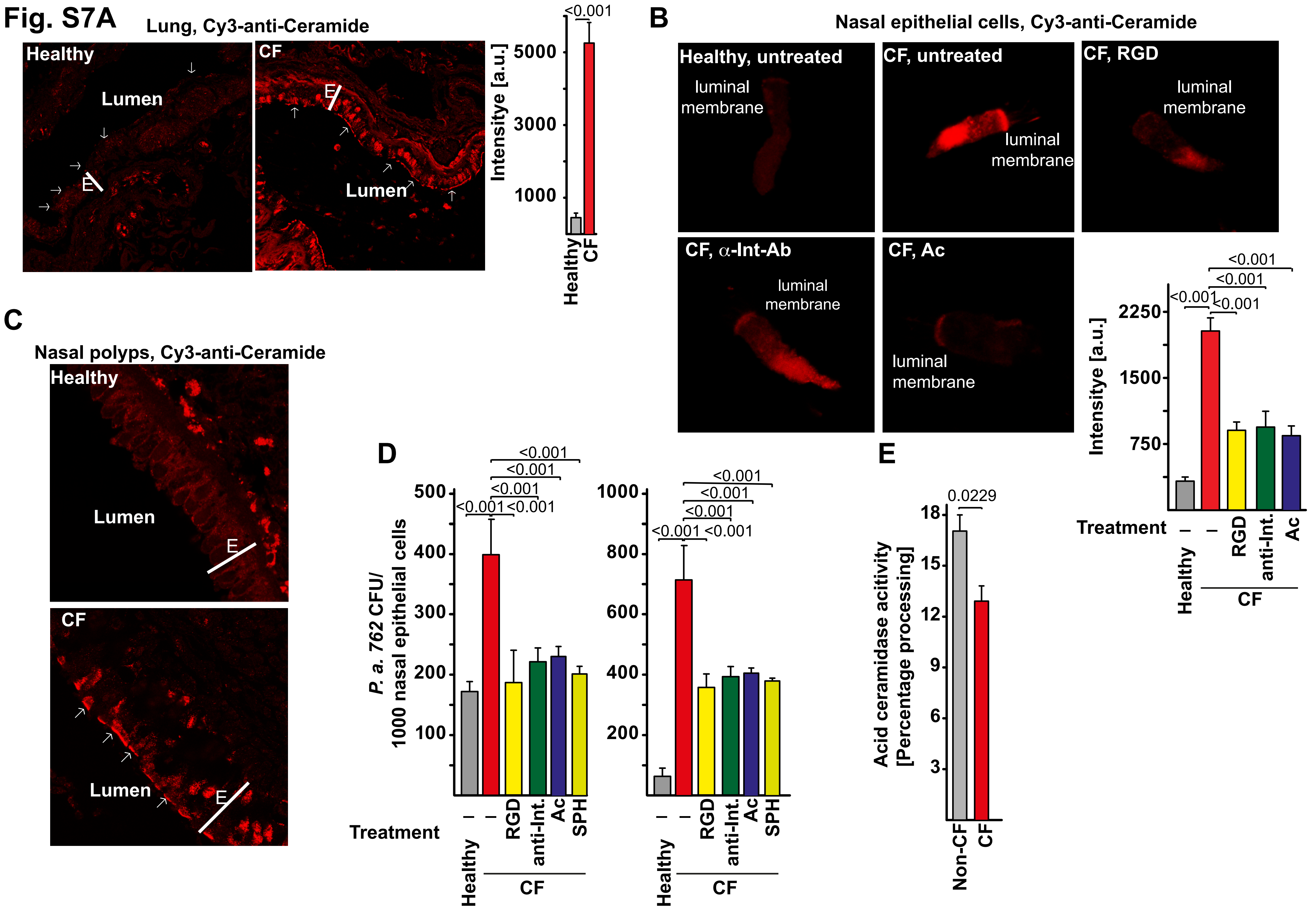
Fig. S7A
Lung, Cy3-anti-Ceramide
Healthy
Lumen
E
CF
E
Lumen
<0.001
Intensitye [a.u.]
5000
3000
1000
Healthy
CF
B
Nasal epithelial cells, Cy3-anti-Ceramide
Healthy, untreated
luminal membrane
CF, untreated
luminal membrane
CF, RGD
luminal membrane
CF, α-Int-Ab
luminal membrane
CF, Ac
luminal membrane
<0.001
<0.001
<0.001
<0.001
2250
1500
750
Intensitye [a.u.]
Treatment
–
–
RGD
anti-Int.
Ac
Healthy
CF
C
Nasal polyps, Cy3-anti-Ceramide
Healthy
Lumen
E
CF
Lumen
E
D
<0.001
<0.001
<0.001
<0.001
<0.001
500
400
300
200
100
P. a. 762 CFU/
1000 nasal epithelial cells
Treatment
–
–
RGD
anti-Int.
Ac
SPH
Healthy
CF
<0.001
<0.001
<0.001
<0.001
<0.001
1000
800
600
400
200
–
–
RGD
anti-Int.
Ac
SPH
Healthy
CF
E
0.0229
18
15
12
9
6
3
Acid ceramidase acitivity
[Percentage processing]
Non-CF
CF
